# *O*-GlcNAcylation of METTL3 drives hepatocellular carcinoma progression by upregulating MCM10 expression in an m6A-IGF2BP3-dependent manner

**DOI:** 10.1038/s41419-025-07844-1

**Published:** 2025-07-12

**Authors:** Zhen Chen, Jiaxin Yin, Zhongqi Feng, Yanlai Zhang, Li Liang, Xiaojun Wang, Kai Wang, Ni Tang

**Affiliations:** 1 Department of Infectious Diseases, Key Laboratory of Molecular Biology for Infectious Diseases (Ministry of Education), Institute for Viral Hepatitis, the Second Affiliated Hospital, Chongqing Medical University, Chongqing, China; 2 Institute of Hepatobiliary Surgery, Southwest Hospital, Third Military Medical University (Army Medical University), Chongqing, China

**Keywords:** Oncogenes, Liver cancer

## Abstract

The m6A methyltransferase METTL3 is a key regulator of RNA m6A modification, which plays a critical role in cancer development. Despite the significance of METTL3 in hepatocellular carcinoma (HCC), its post-translational modifications and their functional implications in HCC remain poorly understood. The present study reveals that METTL3 undergoes *O*-GlcNAcylation, which enhances its stability and promotes HCC progression. Specific *O*-GlcNAcylation sites (T186/S192/S193) in METTL3 are identified. *O*-GlcNAc modification reduces METTL3 ubiquitination, thereby increasing protein stability, and enhances its interaction with WTAP, thereby sustaining m6A levels in hepatoma cells. Notably, METTL3 *O*-GlcNAcylation upregulates the expression of minichromosome maintenance protein 10 (MCM10) by stabilizing its mRNA via an m6A-IGF2BP3-dependent manner. Targeting METTL3 *O*-GlcNAcylation with designed peptides effectively inhibits HCC growth both in vitro and in vivo. Collectively, our findings provide insights into the regulatory role of *O*-GlcNAcylation in modulating the m6A epitranscriptome and suggest the potential therapeutic relevance of targeting METTL3 *O*-GlcNAcylation in HCC.

## Introduction

N6-methyladenosine (m6A) is the most prevalent internal RNA modification in mammals [1]. m6A methylation primarily occurs in the consensus motif RRACH (where R = A/G and H = A/C/U), typically near the 3′-untranslated region (3′-UTR) or at the stop codon [2, 3]. This methylation is catalyzed by the m6A methyltransferase complex (MTC), which is mainly composed of methyltransferase-like 3 (METTL3), METTL14, and Wilms’ tumor 1-associating protein (WTAP) [4–6]. METTL3, the core component of the MTC, functions as an S-adenosylmethionine-binding subunit [7, 8]. Accumulating evidence indicates that METTL3 overexpression is implicated in the initiation and metastasis of various types of cancer [9–13]. Aberrant levels of METTL3 can lead to alterations in overall m6A methylation patterns, thereby impacting RNA stability [14, 15], translation efficiency [16], and RNA export [17], culminating in the disruption of cellular homeostasis and malignant transformation of cells.

The significance of METTL3 in the regulation of m6A methylation is well documented. However, its post-translational modifications (PTMs) and self-regulatory properties in cancer remain to be fully elucidated. SUMOylation of METTL3 has been shown to accelerate the progression of hepatocellular carcinoma (HCC) by repressing its m6A methyltransferase activity [18]. Acetylation of METTL3 impedes its nuclear translocation, suppressing the metastasis of breast cancer [19]. Lactylation of METTL3 is essential for target RNA recognition and maintaining the immunosuppressive capacity of tumor-infiltrating myeloid cells [20]. ERK-phosphorylated METTL3 plays a crucial role in stabilizing the m6A MTC [21]. *O*-glycosylation proteomics have indicated that METTL3 *O*-GlcNAcylation may occur [22, 23]; however, the implications of this modification in cancer remain enigmatic.

*O*-GlcNAcylation involves the enzymatic transfer of *O*-linked β-*N*-acetylglucosamine (*O*-GlcNAc) to the hydroxyl groups of serine and threonine residues in proteins [24]. It has been implicated in various biological processes in cancer by altering the stability [25], activity [26, 27], localization [28], and protein–protein interactions of its targets [29]. Increasing evidence suggests that *O*-GlcNAcylation is closely associated with HCC onset, progression, metastasis, drug resistance, and stemness [30]. Despite advances in our understanding of the role m6A modification in HCC pathogenesis, the crosstalk between protein *O*-GlcNAcylation and RNA m6A modification remains poorly understood. *O*-GlcNAcylation of YTHDF2 at Ser263 has been shown to accelerate the progression of hepatitis B virus-related HCC by increasing its protein levels and stabilizing *MCM2* and *MCM5* [31]. However, the full extent of *O*-GlcNAcylation of m6A “writer” proteins and its role in cancer development remain to be elucidated.

In this study, we observed that T186/S192/S193 *O*-GlcNAcylation of METTL3 significantly promotes the proliferation, invasion, and migration of hepatoma cells in vitro and in vivo*. O*-GlcNAcylation of METTL3 impaired its interaction with FBXW7, leading to decreased ubiquitination and enhanced protein stability. Although *O*-GlcNAcylation of METTL3 had minimal effect on its subcellular localization and methyltransferase activity, it enhanced its interaction with WTAP, thereby sustaining m6A levels in hepatoma cells and stabilizing *MCM10* mRNA via IGF2BP3. These results suggest that the METTL3-*O*-GlcNAcylation/MCM10/IGF2BP3 axis represents a novel mechanism involved in HCC progression.

## Results

### METTL3 is highly *O*-GlcNAcylated in HCC

**Fig. 1:**
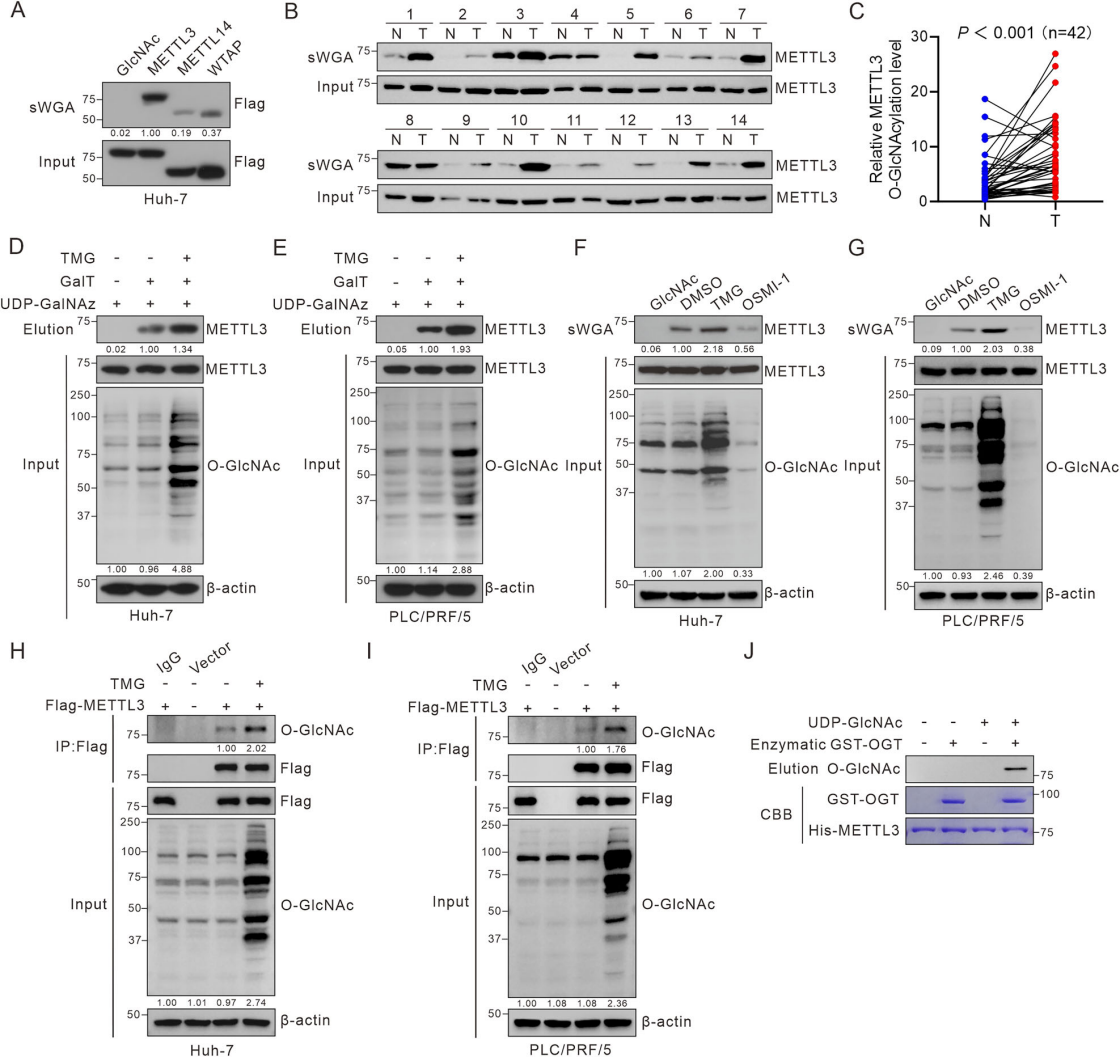
METTL3 is highly *O*-GlcNAcylated in HCC. **A** Huh-7 hepatoma cells were transfected with Flag-METTL3, Flag-METTL14, or Flag-WTAP for 48 h. Cell lysates were collected for sWGA pull-down assays to identify *O*-GlcNAcylation. METTL3 *O*-GlcNAcylation levels in HCC tumors (T) and paired adjacent non-tumor (NT) tissues were detected by sWGA pull-down assays (**B**), quantified using ImageJ and analyzed with a two-tailed paired Student’s *t*-test (**C**), *P* < 0.001. **D**, **E** Analysis of METTL3 *O*-GlcNAcylation using chemoenzymatic labeling in Huh-7 and PLC/PRF/5 hepatoma cells treated with 25 μM TMG for 12 h. **F**, **G** Huh-7 and PLC/PRF/5 cells were treated with 25 μM TMG or 20 μM OSMI-1 for 12 h. Then, cell lysates were pulled down with sWGA-conjugated agarose and immunoblotted with anti-METTL3. **H**, **I** Huh-7 and PLC/PRF/5 hepatoma cells were transfected with Flag-METTL3 or a control vector for 48 h. After treatment with 25 μM TMG for 12 h, cell lysates were immunoprecipitated using anti-Flag M2 agarose beads. **J** For the in vitro *O*-GlcNAcylation assay, recombinant His-METTL3 proteins and enzymatic GST-OGT domain (aa 313–1031) were incubated in reaction buffer for 4 h. Immunoblot analyses and Coomassie blue staining were performed.

The RNA MTC is primarily composed of METTL3, METTL14, and WTAP. To investigate the effect of *O*-GlcNAc modification on m6A writers, we employed the sWGA pull-down assay, as sWGA specifically binds to *O*-GlcNAc on proteins. METTL3 exhibited a higher level of *O*-GlcNAcylation than METTL14 and WTAP in Huh-7 cells (Fig. 1A). This finding led us to focus on the role of *O*-GlcNAcylated METTL3 in HCC. We examined METTL3 protein levels in 42 pairs of HCC and adjacent non-cancerous tissues, which revealed a significant increase in METTL3 protein expression in HCC (Fig. S1A, B). sWGA assay results confirmed that *O*-GlcNAcylation of METTL3 was significantly elevated in HCC tissues (Fig. 1B, C; Fig. S1D). In the TCGA-LIHC dataset, patients with HCC exhibiting high METTL3 expression had a poorer overall survival rate (Fig. S1C).

To investigate the *O*-GlcNAcylation status of METTL3, a chemoenzymatic labeling experiment validated the *O*-GlcNAcylation of METTL3 in both cell lines, and TMG treatment enhanced the level of modification (Fig. 1D, E). sWGA assay results, which mirrored the cellular *O*-GlcNAcylation levels in Huh-7 and PLC/PRF/5 cells, supported the *O*-GlcNAcylation of METTL3. The modification level decreased after treatment with the OGT inhibitor OSMI-1 while increased after TMG treatment (Fig. 1F, G). Moreover, Huh-7 and PLC/PRF/5 cells were transfected with Flag-METTL3. Cell lysates were immunoprecipitated with anti-Flag agarose beads and probed with anti-*O*-GlcNAc antibody. The IP results confirmed *O*-GlcNAcylation of METTL3 in both cell lines, and the level of modification increased after treatment with the *O*-GlcNAcase inhibitor TMG (Fig. 1H, I). An in vitro *O*-GlcNAcylation assay showed that METTL3 was a direct target for *O*-GlcNAcylation by the enzymatic OGT fragment (aa 313–1031), using UDP-GlcNAc (Fig. 1J). Together, these findings indicated that METTL3 is extensively *O*-GlcNAcylated in HCC.

### OGT mediates *O*-GlcNAcylation of METTL3 on Thr186/Ser192/Ser193

**Fig. 2:**
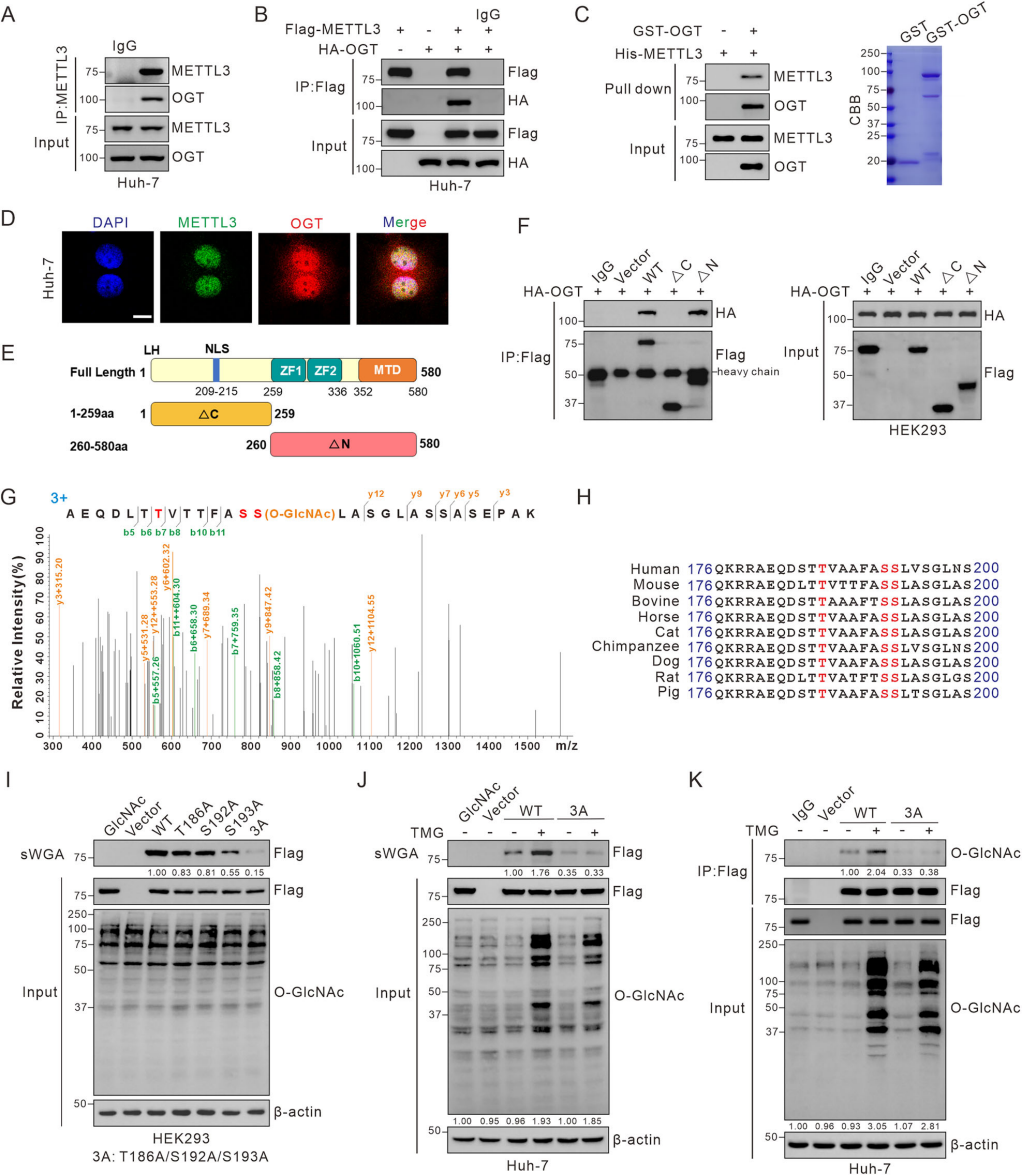
OGT mediates *O*-GlcNAcylation of METTL3 on Thr186/Ser192/Ser193. **A** Co-IP assay of the physical interaction between endogenous METTL3 and OGT in Huh-7 cells. **B** Flag-METTL3 and HA-OGT were transfected into Huh-7 cells. Cell extracts were immunoprecipitated with anti-Flag antibody, followed by immunoblotting with the indicated antibodies. **C** Pull-down assays were performed to observe the direct interaction between METTL3 and OGT in vitro. Immunoblot analysis and Coomassie blue staining are shown. **D** Immunofluorescence staining of METTL3 and OGT in Huh-7 cells. Nuclei were counterstained with DAPI. Scale bar: 10 μm. **E** Schematic representation of the domain structure of METTL3. Full-length METTL3 consists of two domains, a C-terminal region (1–259 aa, ΔC) and the methyltransferase domain (260–580 aa, ΔN). **F** The interactions between HA-OGT and Flag-METTL3 (WT, ΔC, ΔN) were verified by Co-IP in HEK293 cells. **G** LC-MS identified Ser193 as the METTL3 *O-*GlcNAcylation site, which corresponded to *O*-GlcNAcylated METTL3 peptide AEQDLTTVTTFASSLASGLASSASEPAK. **H** Cross-species METTL3 sequence alignment. **I** HEK293 cells were transfected with Flag-tagged METTL3 (WT, T186A, S192A, S193A, or T186A/S192A/S193A). An sWGA pull-down assay was used to identify METTL3 *O*-GlcNAcylation sites. **J**, **K** Huh-7 cells were transfected with Flag-METTL3 WT, 3A mutant, or vector, followed by 25 μM TMG treatment for 12 h. sWGA pull-down (**J**) and IP (**K**) assays were used to identify *O*-GlcNAcylation sites.

OGT is the principal enzyme responsible for the transfer of GlcNAc to serine or threonine residues on target proteins. We examined the interaction and colocalization of METTL3 and OGT using Co-IP assays (Fig. 2A, B; Fig. S2A) and confocal microscopy (Fig. 2D), respectively. Their direct interaction was confirmed through GST-pulldown assays in vitro (Fig. 2C). To identify the interaction domain, we created two METTL3 deletion mutants (ΔC and ΔN, Fig. 2E) and found that the C-terminal region of METTL3 (aa 260–580) is crucial for binding to OGT (Fig. 2F). Liquid chromatography-tandem mass spectrometry (LC-MS/MS) analysis identified Thr186/Ser192/Ser193 as the primary *O*-GlcNAcylation sites on METTL3 (Fig. 2G; Fig. S2B). These residues are conserved across various species (Fig. 2H). We then generated METTL3 mutants (T186A, S192A, S193A, and T186A/S192A/S193A) to evaluate alterations in *O*-GlcNAcylation levels. The 3A mutant (T186A/S192A/S193A) exhibited markedly reduced *O*-GlcNAcylation compared to wild-type (WT) METTL3 in HEK293 cells (Fig. 2I). IP and sWGA assays in Huh-7 and PLC/PRF/5 cells confirmed the reduced *O*-GlcNAcylation in the METTL3-3A mutant, with no remarkable changes after TMG treatment (Fig. 2J, K; Fig. S2C). These results demonstrated that Thr186/Ser192/Ser193 are the primary *O*-GlcNAcylation sites on METTL3.

### METTL3 *O*-GlcNAcylation promotes HCC proliferation, invasion, and migration

**Fig. 3:**
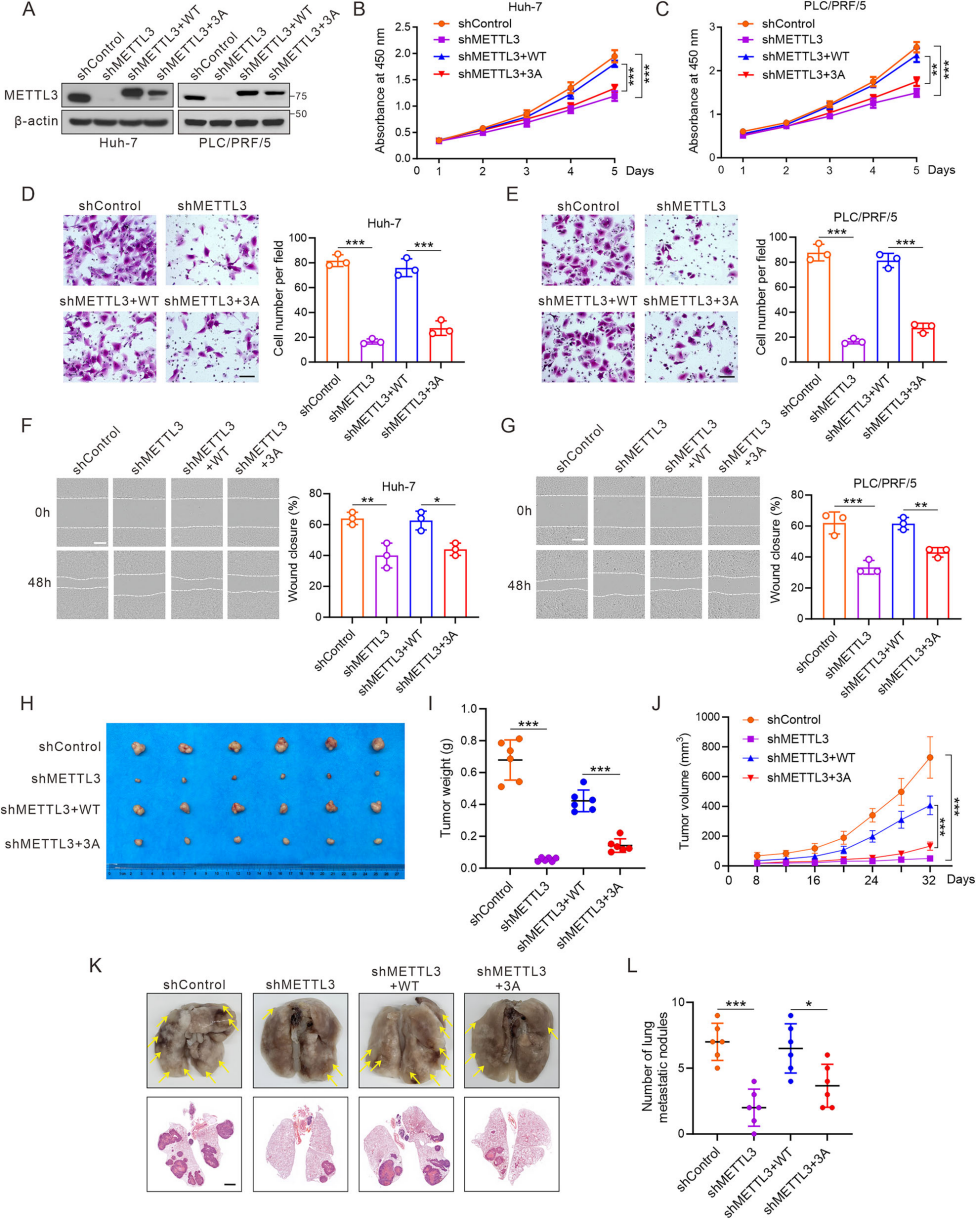
*O*-GlcNAcylation of METTL3 at Thr186/Ser192/Ser193 enhances the oncogenic capacity of hepatoma cells in vitro and in vivo. Huh-7 and PCL/RPF/5 hepatoma cells were infected with METTL3 shRNA lentivirus to decrease endogenous METTL3 levels, followed by infection with adenoviruses expressing Flag-METTL3 (WT or 3A). **A** Immunoblotting of METTL3 in treated Huh-7 and PCL/PRF/5 hepatoma cells. **B**, **C** Growth curves of treated hepatoma cells based on a CCK-8 assay (*n* = 3 independent experiments). **D**, **E** Representative images (100×) and quantitative analysis of cell migration capacity (*n* = 3, performed in triplicate). Scale bar: 100 μm. **F**, **G** Representative images and quantitative results of wound-healing assays of hepatoma cells (100×, *n* = 3 independent experiments). Scale bar: 200 μm. **H**–**J** MHCC-97H cells were processed as described above and injected subcutaneously into nude mice (*n* = 6 per group). **H** Representative images of xenograft tumors are shown. Tumor weight (**I**) and volume (**J**) were measured and calculated. **K**, **L** MHCC-97H cells were treated as described above and injected into nude mice via tail vein injection. Representative images of lung metastases (upper panel) and H&E staining (lower panel) are shown (**K**) and were quantitatively analyzed (**L**). Scale bar: 2000 μm. All data are shown as mean ± SD. One-way followed by the Tukey test, *P < 0.05, **P < 0.01, ***P < 0.001.

METTL3 regulates various cellular processes, such as cell growth, apoptosis, migration, and invasion [32]. To determine the precise effect of METTL3 *O*-GlcNAcylation on HCC development, we knocked down endogenous METTL3 in HCC cell lines using an shRNA targeting its 3′-UTR and then reintroduced either WT or the 3A mutant of METTL3 using adenoviral vectors (Fig. 3A; Fig. S3A). CCK-8 and colony formation assays revealed that METTL3 knockdown significantly inhibited hepatoma cell proliferation, and this effect was reversed by the reintroduction of METTL3-WT, but not METTL3-3A (Fig. 3B, C; Fig. S3B–E). Transwell and wound-healing assays indicated that METTL3 knockdown substantially decreased the migratory and invasive potential of hepatoma cells. Re-expression of METTL3-WT, but not of METTL3-3A, restored cell invasion and migration (Fig. 3D–G; Fig. S3F).

To evaluate the effect of METTL3 *O*-GlcNAcylation on HCC tumorigenesis in vivo, we performed xenograft experiments in nude mice, which showed that METTL3 depletion significantly hindered HCC growth, as indicated by decreased tumor volume, weight, and proliferating cell nuclear antigen IHC. This effect was rescued by METTL3-WT, but not by METTL3-3A (Fig. 3H–J; Fig. S3G). To explore the role of METTL3 in HCC metastasis, MHCC-97H cells were injected into BALB/c nude mice via the tail vein to assess lung colonization. The number of lung tumors from METTL3-knockdown MHCC-97H cells was significantly lower than in the control group, and reintroduction of METTL3-WT, but not METTL3-3A, restored lung colonization (Fig. 3K, L). Thus, *O*-GlcNAcylation of METTL3 promoted HCC progression in both cellular and animal models.

### *O*-GlcNAcylation stabilizes METTL3 by reducing ubiquitination

**Fig. 4:**
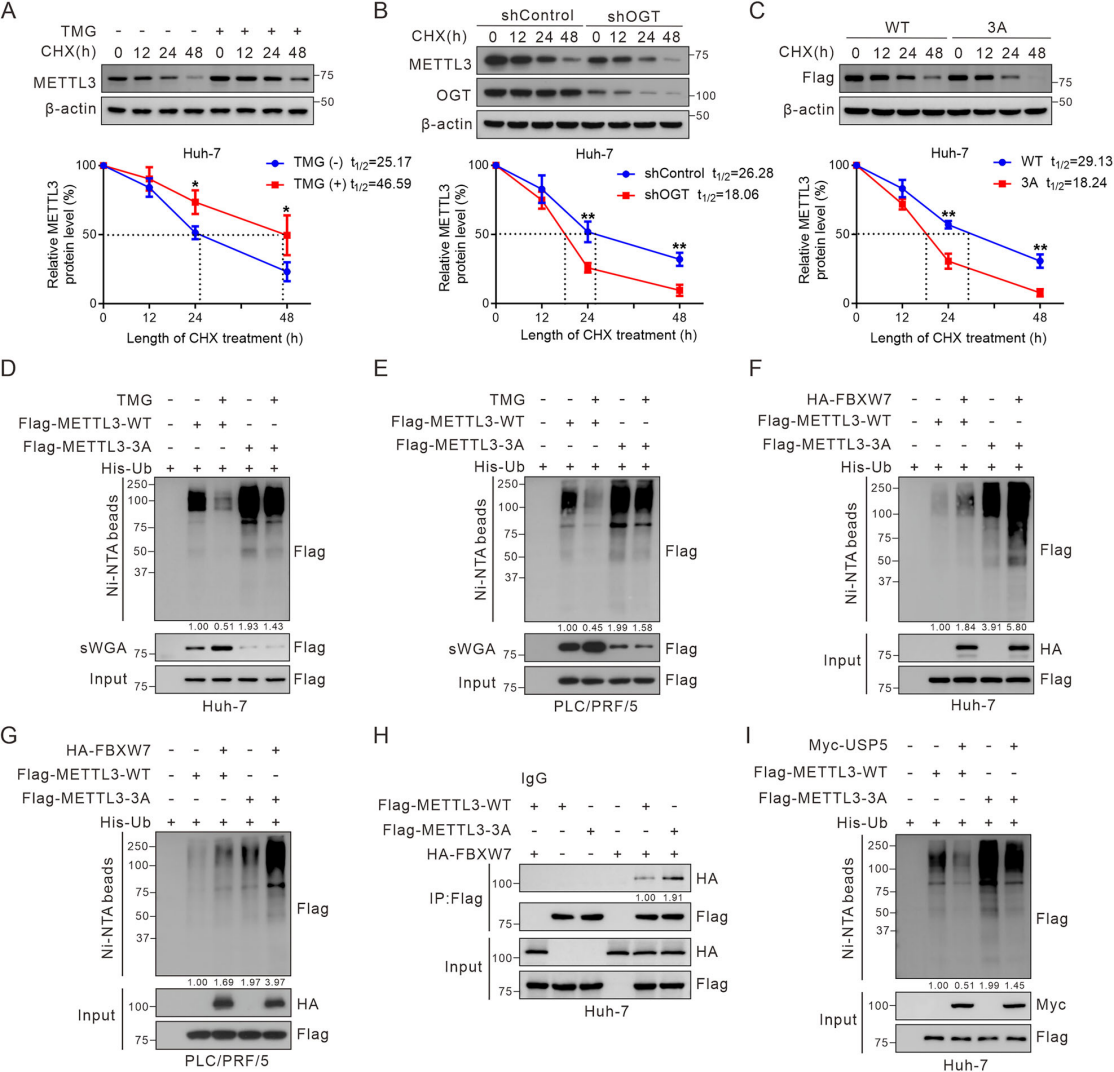
*O-*GlcNAcylation stabilizes METTL3 by decreasing its ubiquitination. **A** Immunoblotting of METTL3. Huh-7 cells were treated with 25 μM TMG for 12 h. Then, 100 μM CHX was added to block protein synthesis for the indicated times. The half-life of METTL3 was quantified in three independent immunoblotting experiments. METTL3 levels at 0 h were arbitrarily set to 100%. **B** Half-life and quantitative analysis of METTL3. Huh-7 cells were infected with OGT shRNA lentivirus and treated with 100 μM CHX for the indicated times. METTL3 levels were measured by immunoblotting (*n* = 3). **C** Half-life of Flag-METTL3 and quantitative analysis in Huh-7 cells. Huh-7 cells were transfected with Flag-METTL3-WT or -3A and treated with 100 μM CHX for the indicated times. Cell lysates were immunoblotted with anti-Flag. Data are representative of three independent experiments. Data in (**A**–**C**) were analyzed using an unpaired Student’s *t*-test. *P < 0.05, **P < 0.01. **D**, **E** Hepatoma cells were co-transfected with His-Ub and Flag-METTL3 (WT or 3A) and treated or untreated with TMG. Cells were treated with 20 μM MG132 for 8 h, and cell lysates were subjected to IP. Input and IP proteins were immunoblotted with indicated antibodies. FBXW7 increases the poly-ubiquitination level of METTL3 in Huh-7 (**F**) and PCL/PRF/5 cells (**G**). **H** Huh-7 cells were co-transfected Flag-METTL3 (WT or 3A) and HA-FBXW7, cell lysates were subjected to Co-IP to observe their interactions. **I** USP5 decreases the poly-ubiquitination level of METTL3 in Huh-7 cells.

*O*-GlcNAcylation is key in regulating protein localization, stability, and molecular interactions [33]. To explore the effects of *O*-GlcNAcylation on METTL3 protein stability, we evaluated endogenous METTL3 levels in Huh-7 and PLC/PRF/5 cells after treatment with TMG or shOGT lentivirus and CHX using immunoblotting at specified time points. TMG, which promotes *O*-GlcNAcylation of METTL3, significantly extended the half-life of METTL3, whereas shOGT had the reverse effect (Fig. 4A, B; Fig. S4A, B). The half-life of METTL3-3A was shorter than that of METTL3-WT in hepatoma cells (Fig. 4C; Fig. S4C). The addition of the proteasome inhibitor MG132 led to a significant increase in METTL3 protein levels, whereas chloroquine had no significant effect, indicating that METTL3 is degraded primarily via the ubiquitin-proteasome pathway (Fig. S4D, E).

To elucidate the mechanism of *O*-GlcNAcylation in METTL3 stabilization, we used in vivo ubiquitination assays. The ubiquitination level of the METTL3-3A mutant was markedly higher than that of the METTL3-WT. TMG treatment led to a significant reduction in METTL3-WT ubiquitination, with minimal effect on METTL3-3A (Fig. 4D, E). Given that E3 ubiquitin ligases and deubiquitinating enzymes modulate protein stability [34, 35], we transfected Huh-7 cells with candidate E3 ubiquitin ligases, including SPOP, FBXW7, and CSTA. FBXW7 significantly reduced METTL3 expression in Huh-7 cells (Fig. S4F, G). IP assays indicated that FBXW7 increased METTL3 ubiquitination, particularly in the METTL3-3A mutant (Fig. 4F, G). Co-IP assays revealed a stronger interaction between METTL3-3A and FBXW7 than between FBXW7 and METTL3-WT (Fig. 4H). Building on the work of Sun et al. [21], which highlighted the role of USP5 in ERK-mediated METTL3 stabilization, we investigated whether USP5 also regulates *O*-GlcNAcylated METTL3. USP5 significantly elevated METTL3 expression and reduced METTL3 ubiquitination in hepatoma cells (Fig. 4I; Fig. S4I, K). Moreover, MG132 reversed FBXW7-induced METTL3 downregulation and potentiated USP5-induced METTL3 upregulation (Fig. S4H, J). These results suggested that *O*-GlcNAcylation enhances METTL3 stability by suppressing its ubiquitination.

### METTL3 O-GlcNAcylation targets *MCM10* mRNA to maintain tumorigenic behavior of hepatoma cells

**Fig. 5:**
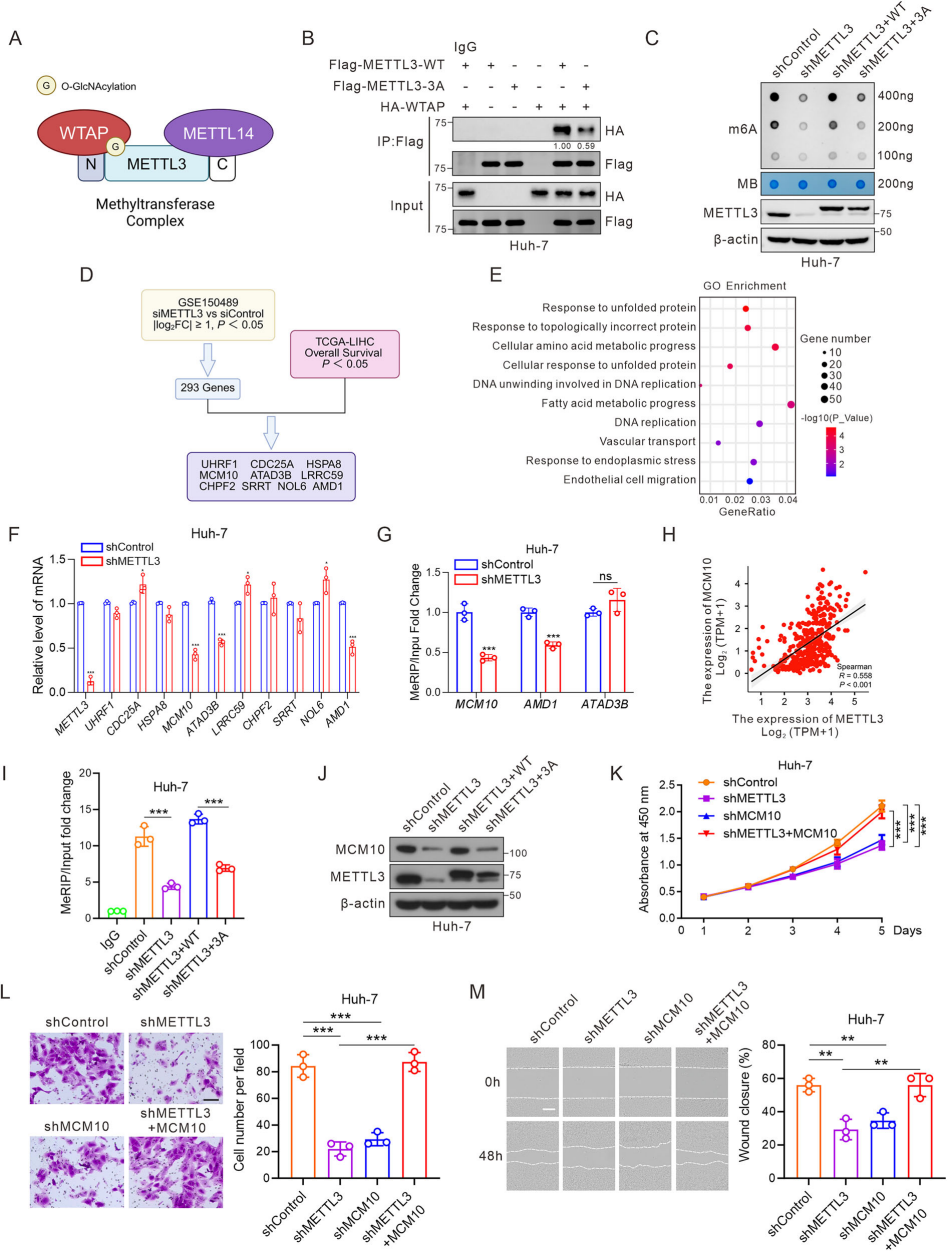
METTL3 *O*-GlcNAcylation targets *MCM10* mRNA to maintain the tumorigenic behavior of hepatoma cells. **A** Schematic representation of the METTL3-METTL14-WTAP complex. *O*-GlcNAcylation of METTL3 enhances its affinity for WTAP, without affecting its association with METTL14. **B** Flag-METTL3 (WT or 3A) was transfected with or without HA-WTAP into Huh-7 cells. Lysates were immunoprecipitated with anti-Flag antibody, followed by immunoblotting with specific antibodies. **C** Huh-7-shMETTL3 cells were transfected with Flag-METTL3-WT or -3A. m6A abundance in mRNAs from infected cells was measured using the dot-blot assay with an anti-m6A antibody, with mRNA loading confirmed by methylene blue staining (upper panels). Cell lysates were immunoblotted with the indicated antibodies (lower panels). **D** Screening for downstream targets of METTL3 by bioinformatic analysis. **E** Gene Ontology analysis of METTL3-knockdown differentially expressed genes. The mRNA levels of initially screened genes in Huh-7-shMETTL3 cells were measured using RT-qPCR (**F**), and the m6A modification levels of target mRNAs were assessed using MeRIP-qPCR (**G**) (*n* = 3 independent experiments). **H** Correlation analysis of *METTL3* and *MCM10* in the TCGA-LIHC cohort (Spearman correlation, *P* < 0.001). **I** MeRIP-qPCR assessment of the effect of METTL3 *O*-GlcNAcylation on m6A modification of *MCM10* mRNA. **J** Immunoblotting to assess the effect of METTL3 *O*-GlcNAcylation on MCM10 protein levels. **K**–**M** Huh-7 cells were primarily infected with lentiviruses carrying shControl, shMETTL3, or shMCM10, followed by infection with either Ad-GFP or Ad-MCM10. All treatment groups were subjected to CCK-8 (**K**), Transwell (**L**, scale bar: 100 μm), and wound-healing (**M**, scale bar: 200 μm) assays. Results are derived from three independent experiments and are presented as mean ± SD. Data in (**F**, **G**) were analyzed using an unpaired, two-tailed Student’s *t*-test. Data in (**I**, **K**–**M**) were analyzed using one-way ANOVA followed by Tukey tests. *P < 0.05, **P < 0.01, ***P < 0.001.

We next examined the influence of METTL3 *O*-GlcNAcylation on its nuclear localization. Immunofluorescence and nuclear/cytosolic fractionation assays showed that neither TMG nor OSMI-1 influenced the cellular distribution of METTL3, although they significantly altered METTL3 protein levels (Fig. S5A–D). To validate this finding, Huh-7 and PLC/PRF/5 cells were infected with adenoviruses expressing Flag-METTL3 (WT or 3A). Nuclear localization did not differ between the two groups (Fig. S5E–G). RNA m6A methylation is catalyzed by the MTC, which primarily comprises METTL3, METTL14, and WTAP. As *O*-GlcNAcylation primarily modulates protein–protein interactions to regulate cellular functions, we employed Co-IP assays to determine if METTL3 O-GlcNAcylation modulates its binding to METTL14 or WTAP. *O*-GlcNAcylated METTL3 exhibited increased WTAP binding, which was further enhanced by TMG treatment but significantly reduced by OSMI-1 (Fig. 5A, B; Fig. S6A). However, *O*-GlcNAcylation did not affect METTL3–METTL14 interaction (Fig. S6B). We also explored whether *O*-GlcNAcylation affects the m6A RNA methyltransferase activity of METTL3, but found no significant differences in activity between purified Flag-METTL3-WT and -3A proteins (Fig. S6C).

We next explored the effect of METTL3 *O*-GlcNAcylation on global m6A methylation levels in mRNA. Depletion of METTL3 using shRNA led to a marked decrease in m6A levels in Huh-7 and PLC/PRF/5 cells, which was rescued by reintroducing METTL3-WT, but not -3A (Fig. 5C; Fig. S6D). To identify potential targets modulated by METTL3 *O*-GlcNAcylation, we analyzed RNA-sequencing data from Huh-7 cells treated with siMETTL3 or siControl (GSE150489), identifying 293 differentially expressed genes (Fig. 5D). Gene ontology analysis showed that the differentially expressed genes were enriched in pathways associated with protein folding, amino-acid metabolism, DNA replication, and cell migration (Fig. 5E). Subsequently, we screened 10 candidate genes related to prognosis in patients with liver cancer (Fig. 5D). *MCM10*, *ATAD3B*, and *AMD1* were significantly downregulated upon METTL3 knockdown in Huh-7 cells (Fig. 5F), with *MCM10* and *AMD1* also showing reduced m6A modification (Fig. 5G). MCM10 is an essential replication factor in all eukaryotes and regulates DNA replication [36]. Aberrant MCM10 expression is involved in cancer growth and metastasis [37–39]. We identified a positive correlation between *METTL3* and *MCM10* mRNA levels in the TCGA-LIHC database (Fig. 5H). MeRIP-qPCR confirmed reduced m6A modification on *MCM10* mRNA in the METTL3-3A group compared to the METTL3-WT group (Fig. 5I). Knockdown of METTL3 results in a reduction of MCM10 protein levels, which can be restored by wild-type METTL3 but not by the 3 A mutant (Fig. 5J; Fig. S6E). To assess the role of MCM10 and its correlation with METTL3 in HCC progression, we found that knockdown of MCM10 inhibited the proliferation, invasion, and migration of hepatoma cells, while overexpression of MCM10 rescued the malignant phenotypes in METTL3-deficient cells (Fig. 5K–M; Fig. S6F–H). These findings indicated that METTL3 *O*-GlcNAcylation targets *MCM10* mRNA to regulate HCC progression.

### METTL3 *O*-GlcNAcylation enhances *MCM10* mRNA stability in an m6A-IGF2BP3-dependent manner

**Fig. 6:**
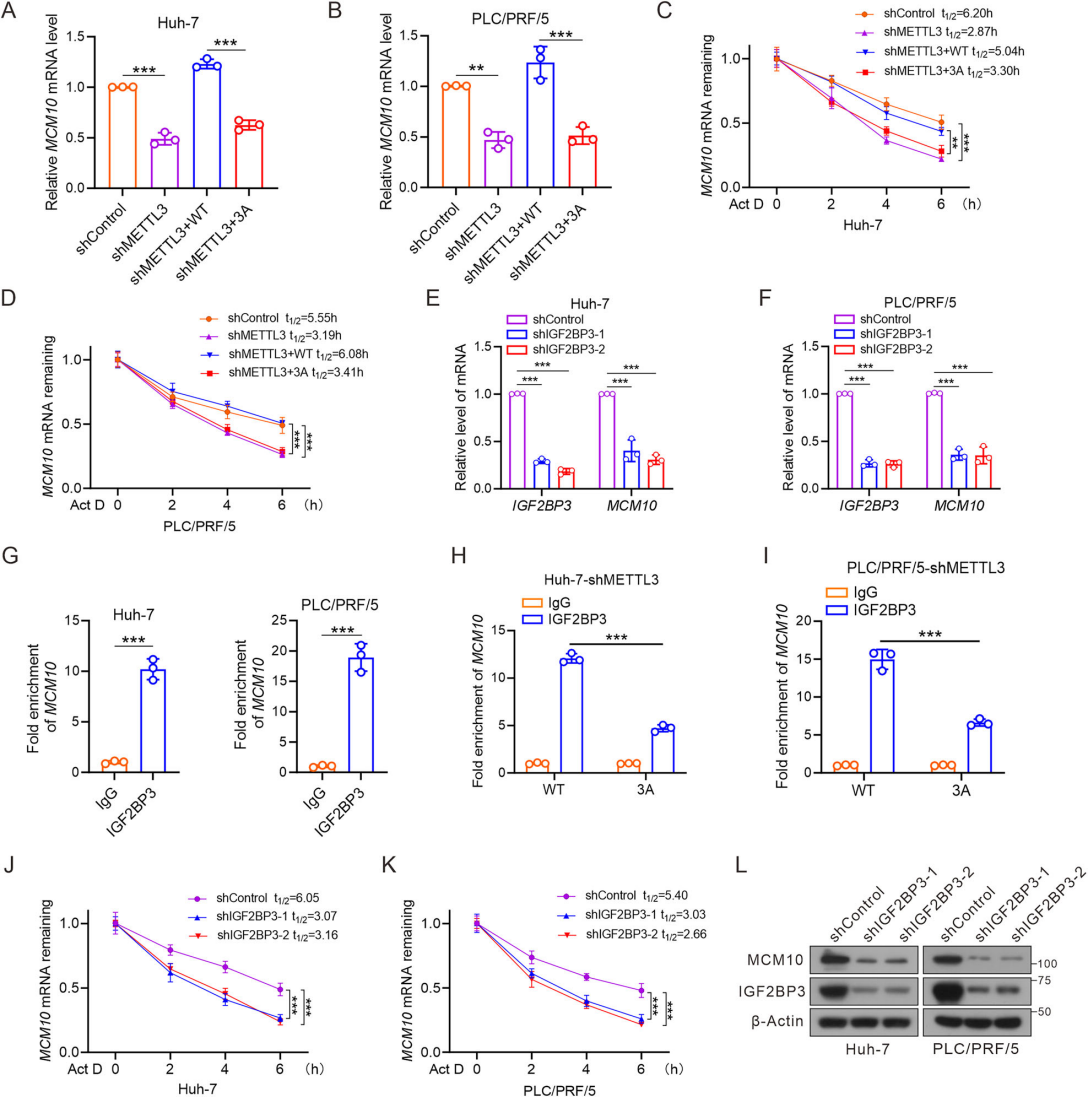
METTL3 *O*-GlcNAcylation enhances *MCM10* mRNA stability in an m6A-IGF2BP3-dependent manner. **A**, **B** RT-qPCR to observe the effect of METTL3 *O*-GlcNAcylation on relative *MCM10* mRNA expression in hepatoma cells. **C**, **D** Half-life of *MCM10* mRNA in Huh-7 and PLC/PRF/5 cells infected with shMETTL3 lentivirus followed by transfection with Ad-METTL3 (WT or 3A). Transcription was inhibited by actinomycin D (5 μg/mL). **E**, **F** Huh-7 and PLC/PRF/5 cells were transfected with shControl or shIGF2BP3 lentivirus. Thirty-six hours after infection, cell lysates were subjected to RT-qPCR to observe *MCM10* mRNA expression. **G** RIP-qPCR assay of *MCM10* using IgG or IGF2BP3 antibodies in Huh-7 and PLC/PRF/5 cells. **H**, **I** RIP-qPCR assay of *MCM10* in Huh-7-shMETTL3-WT (or 3A mutant) and PLC/PRF/5-shMETTL3-WT (or 3A mutant) cells. Half-life of *MCM10* mRNA in Huh-7 (**J**) and PLC/PRF/5 (**K**) cells infected with shIGF2BP3 lentivirus. **L** Western blot analysis of MCM10 levels in Huh-7 and PLC/PRF/5 cells infected with IGF2BP3 shRNA. All data represent mean ± SD from three independent experiments. Data in (**A**–**F** and **H**–**K**) were analyzed using one-way ANOVA followed by Tukey tests. Data in (**G**) were analyzed using an unpaired, two-tailed Student’s *t*-test. **P < 0.01, ***P < 0.001.

To elucidate the molecular mechanism through which METTL3 *O*-GlcNAcylation modulates *MCM10* mRNA, Huh-7 and PLC/PRF/5 cells were transduced with lentiviruses encoding shMETTL3, followed by the introduction of adenoviral METTL3-WT or -3A. METTL3 knockdown significantly reduced *MCM10* mRNA levels, and this effect was reversed by METTL3-WT, not -3A (Fig. 6A, B). We performed mRNA stability assays in Huh-7 and PLC/PRF/5 cells to investigate whether METTL3 upregulated *MCM10* mRNA levels by enhancing its stability. The decreased half-life of *MCM10* mRNA observed in METTL3 knockdown cells is mitigated by the expression of wild-type METTL3 (Fig. 6C, D). m6A reader proteins recognize m6A-modified mRNAs and mediate additional functions. To identify potential readers involved in m6A modification of *MCM10*, we used RBPsuite (http://www.csbio.sjtu.edu.cn/bioinf/RBPsuite/) and identified 223 candidate RNA-binding proteins (Supplementary Table S1). Insulin-like growth factor 2 mRNA-binding proteins (IGF2BPs), a family of m6A readers, recognize the m6A consensus sequence and stabilize target mRNAs. RIP-seq analysis identified IGF2BP1/2/3 as potential RNA-binding proteins for *MCM10* [40]. We knocked down IGF2BP1/2/3 expression using shRNA lentivirus and observed *MCM10* mRNA levels. IGF2BP3 knockdown significantly reduced *MCM10* expression in Huh-7 and PLC/PRF/5 cells (Fig. 6E, F), whereas no remarkable changes were observed upon IGF2BP1 and IGF2BP2 silencing (Fig. S6I–L). RIP-qPCR analysis confirmed a direct interaction between IGF2BP3 and *MCM10* mRNA in hepatoma cells (Fig. 6G), with METTL3-WT interacting stronger than METTL3-3A (Fig. 6H, I). The decay rate of *MCM10* mRNA decreased upon IGF2BP3 knockdown (Fig. 6J, K). MCM10 protein levels were significantly decreased in IGF2BP3 knockdown cells (Fig. 6L). These results indicated that METTL3 *O*-GlcNAcylation stabilizes *MCM10* mRNA via an m6A-IGF2BP3-dependent manner.

### Targeting METTL3 *O*-GlcNAcylation suppresses its oncogenic role in HCC

**Fig. 7:**
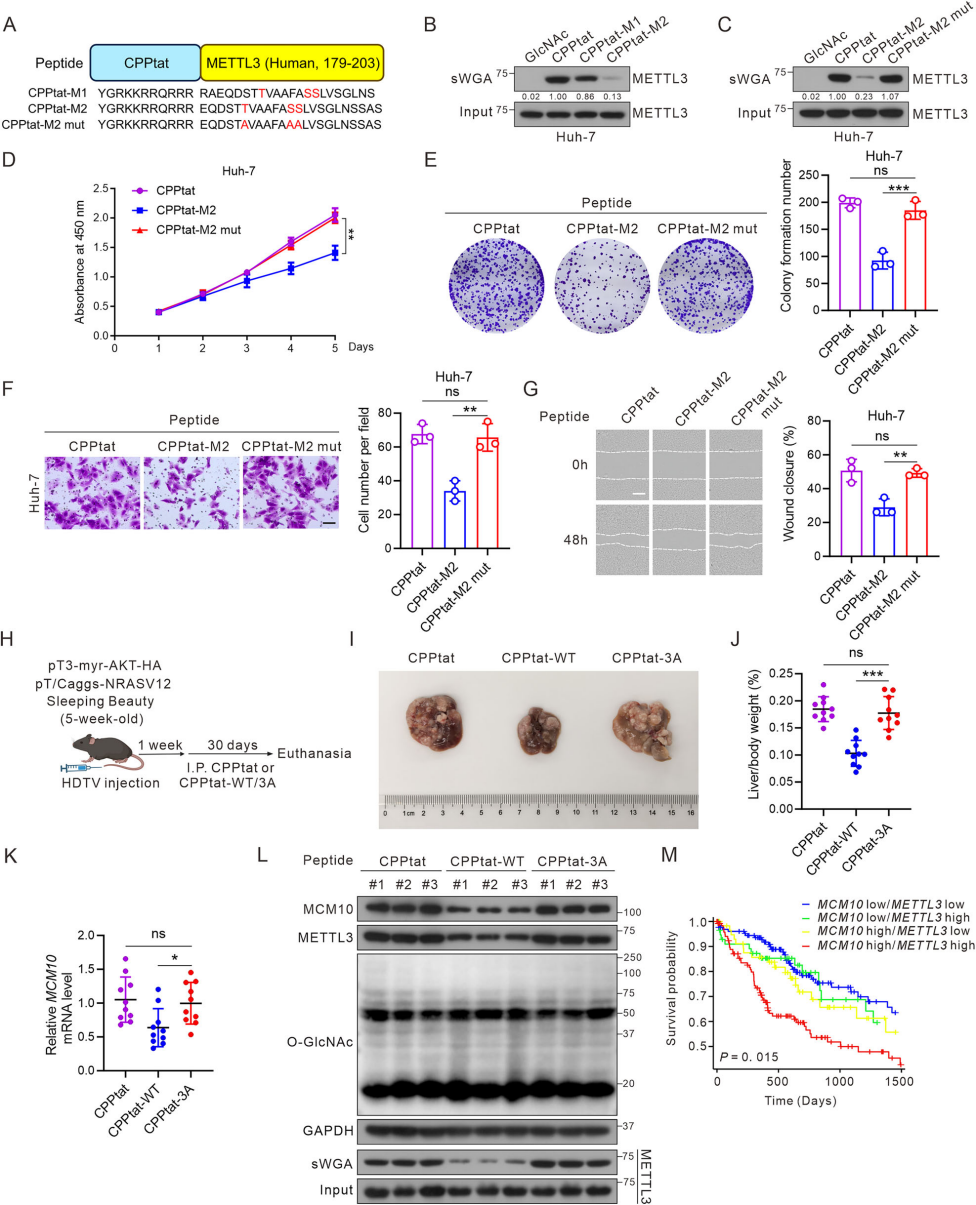
Targeting METTL3 *O*-GlcNAcylation suppresses its oncogenic role in HCC. **A** Schematic representations of the CPPtat-M1, CPPtat-M2, and CPPtat-M2 mut peptides. Differential residues are highlighted in red. **B**, **C** Huh-7 cells were treated with CPPtat, CPPtat-M1, CPPtat-M2, or CPPtat-M2 mut (10 µM) for 24 h, followed by an sWGA pull-down assay to asses METTL3 *O*-GlcNAcylation. CPPtat-, CPPtat-M2-, CPPtat-M2 mut-treated cells were subjected to CCK-8 (**D**), colony formation (**E**), Transwell (**F**, scale bar: 100 μm), and wound-healing (**G**, scale bar: 200 μm) assays. Representative images from three independent experiments are shown. **H** Schematic diagram of the timeline of cell-penetrating peptide (CPPtat, CPPtat-WT, or CPPtat-3A) treatment in the AKT/NRASV12 mouse model. The spontaneous HCC mouse model was established by hydrodynamic tail vein infection. Subsequently, 100 mg/kg of the polypeptides were injected intraperitoneally into mice every 5 days. The schematic was created with BioRender.com (License #RZ28F649PJ, ©2025). **I** Representative livers of AKT/NRASV12 mice treated with CPPtat, CPPtat-WT, or CPPtat-3A. **J** The ratio of liver to body weight was calculated (*n* = 10 mice per group). HCC tissues were harvested for RT-qPCR (**K**), immunoblotting and sWGA pull-down assays (**L**). For (**J**, **K**), data are presented as mean ± SD, *P < 0.05, ***P < 0.001. **M** Kaplan–Meier survival curves illustrating the overall survival (OS) of patients with HCC in the TCGA-LIHC cohort according to *METTL3* and *MCM10* levels, *P* < 0.05.

To further investigate the effect of METTL3 *O*-GlcNAcylation on HCC progression, we synthesized candidate peptides CPPtat-M1/M2 based on the identified *O*-GlcNAcylation motif of METTL3 (179–203) to inhibit this modification (Fig. 7A). The CPPtat sequence was added to enable the peptides to penetrate cells and used as a negative control [41]. CPP-M2, not CPP-M1, significantly attenuated METTL3 *O*-GlcNAcylation in hepatoma cells (Fig. 7B; Fig. S7A). Additionally, CPPtat-M2 mut, incorporating the METTL3-3A mutation, was synthesized (Fig. 7C; Fig. S7B). CPPtat-M2, not CPPtat-M2 mut, markedly inhibited hepatoma cell growth and colony formation (Fig. 7D, E; Fig. S7C, D). CPP-M2 also inhibited hepatoma cell invasion and migration (Fig. 7F, G; Fig. S7E, F). To explore the physiological role of METTL3 *O*-GlcNAcylation in vivo, we established an HCC mouse model by high-volume tail vein injection of plasmids containing SB100, AKT, and NRASV12 (Fig. 7H). Considering the genetic polymorphism across species, we developed CPPtat-WT peptides that specifically target the T186/S192/S193 sites of Mouse METTL3, as well as CPPtat-3A peptides that target the METTL3-3A mutation (Fig. S7G). Treatment with CPPtat-WT significantly inhibited HCC growth compared to treatment with CPPtat and CPPtat-3A (Fig. 7I, J). The sWGA assay and RT-qPCR provided additional evidence that CPPtat-WT suppressed both METTL3 *O*-GlcNAcylation and MCM10 expression (Fig. 7K, L). Survival analysis revealed that patients with elevated mRNA levels of both *METTL3* and *MCM10* had a reduced overall survival rate (Fig. 7M). Collectively, these findings indicated that peptides targeting METTL3 *O*-GlcNAcylation effectively inhibit HCC growth and metastasis.

## Discussion

**Fig. 8:**
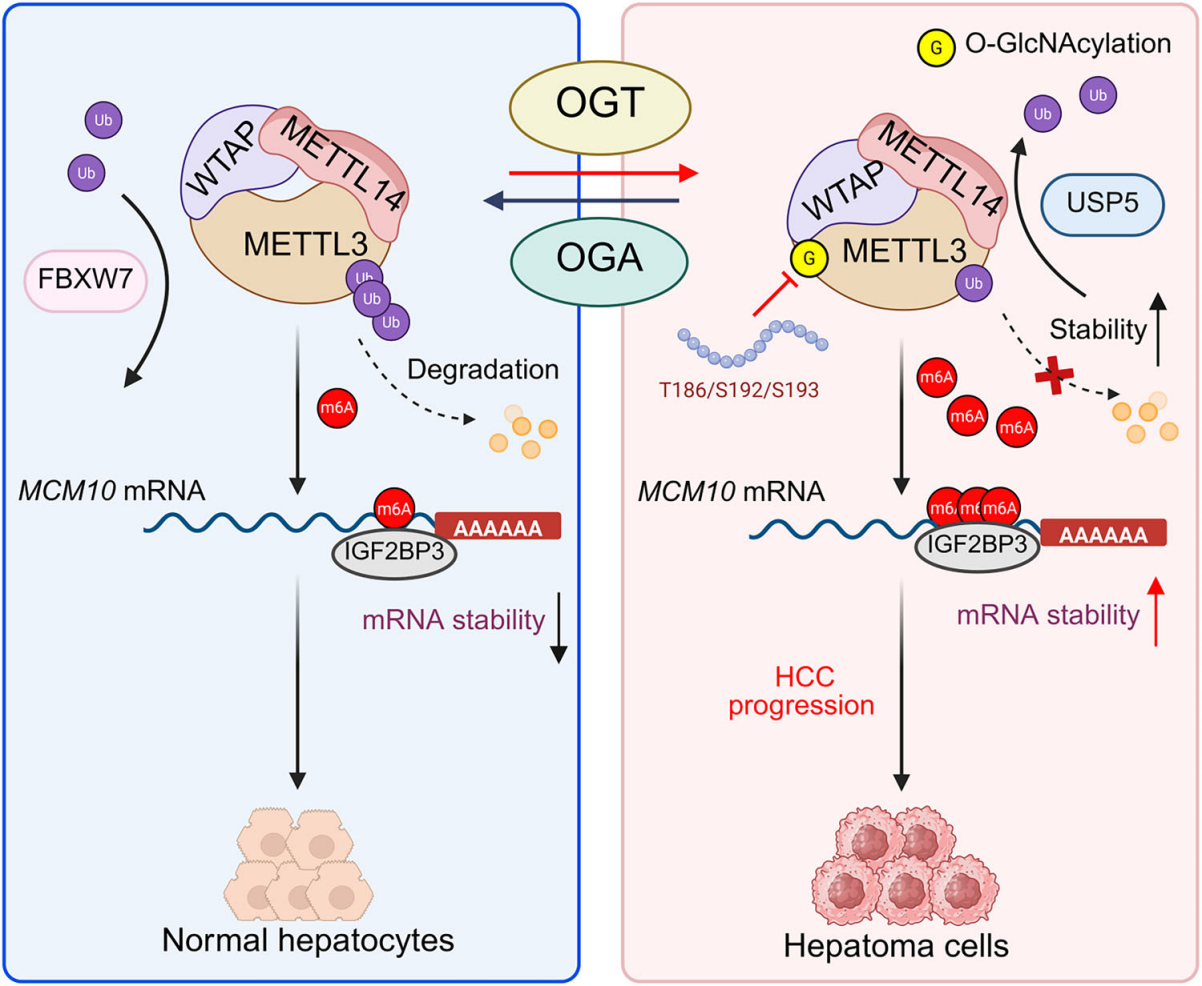
Schematic model of how METTL3 *O*-GlcNAcylation promotes HCC progression. *O*-GlcNAcylation of METTL3 enhances its stability and interaction with WTAP, upregulating *MCM10* mRNA levels in an m6A-IGF2BP3-dependent manner. Created with BioRender.com (License #VY28F5EEOY, ©2025).

RNA m6A modification is increasingly recognized for its pivotal role in human cancer initiation and progression [42]. As a central component of the m6A methylation machinery, METTL3 contributes to HCC carcinogenesis and progression in an m6A-dependent manner. Here, we revealed a novel layer of METTL3 regulation through *O*-GlcNAcylation, a PTM that has been increasingly implicated in HCC progression [30]. *O*-GlcNAcylation of METTL3 at Thr186/Ser192/Ser193 markedly enhanced hepatoma cell proliferation and metastasis. METTL3 *O*-GlcNAcylation elevated m6A levels in hepatoma cells by enhancing its stability and interaction with WTAP. Moreover, METTL3 *O*-GlcNAcylation promoted *MCM10* expression by enhancing its mRNA stability in an m6A-IGF2BP3-dependent manner. Peptides that target METTL3 *O*-GlcNAcylation sites represent a novel approach to suppressing HCC progression (Fig. 8).

PTMs of RNA-modifying proteins play crucial roles in regulating cellular dynamics and disease progression by altering protein stability, activity, localization, and interaction, particularly in cancer growth and metastasis [43]. Our research, along with that of others, suggests that dysregulated PTM of METTL3 significantly contributes to tumor initiation and progression. SUMOylation of METTL3 has been shown to accelerate HCC progression via stabilizing *Snail* mRNA in a manner dependent on its m6A methyltransferase activity [18]. METTL3 acetylation has been found to abrogate its nuclear translocation and impair breast cancer cell migration and invasion [19]. Lactate in tumor-infiltrating myeloid cells upregulates METTL3 expression, and METTL3 lactylation is essential for capturing target RNA and maintaining the immunosuppressive capacity of tumor-infiltrating myeloid cells [20]. ERK-phosphorylated METTL3 and WTAP are vital for m6A MTC stabilization, influencing stem cell differentiation and oncogenesis [21]. We discovered a novel pathway in which *O*-GlcNAcylation regulates METTL3 and its targets in HCC, which aligns with the burgeoning consensus that PTMs are central to the modulation of RNA-modifying proteins, with profound implications for the epitranscriptome and disease progression.

Dysregulated *O*-GlcNAcylation is a cancer hallmark implicated in disease initiation and progression through the modulation of protein function and cellular signaling [44, 45]. Substantial evidence suggests a complex interplay between *O*-GlcNAcylation and ubiquitination, which can be synergistic or antagonistic [46, 47]. *O*-GlcNAcylation exerts a significant effect on ubiquitination by altering proteasome activity [48], the ubiquitination process, ubiquitin-mediated protein degradation [49], and the function of ubiquitination-related enzymes [50]. Furthermore, *O*-GlcNAcylation regulates protein ubiquitination by interacting with phosphorylation events. For instance, *O*-GlcNAcylation of p53 at S149 strongly inhibits T155 phosphorylation by the COP9 signalosome, reducing p53 ubiquitination and degradation [51]. Our study confirmed that METTL3 *O*-GlcNAcylation at Thr186/Ser192/Ser193 markedly reduces its ubiquitination. Notably, we observed that METTL3 *O*-GlcNAcylation significantly hinders its interaction with FBXW7, leading to the decreased ubiquitination and enhanced protein stability. However, the potential antagonistic effect of METTL3 *O*-GlcNAcylation on protein ubiquitination and stability through interference with phosphorylation merits further exploration.

RNA modifications influence gene expression at the post-transcriptional level via a sophisticated regulatory mechanism. However, the interplay between *O*-GlcNAcylation and RNA m6A methylation was not well characterized. *O*-GlcNAcylation stabilizes YTHDF2, enhancing its oncogenic potential by preventing ubiquitination and upregulating *MCM2* and *MCM5* mRNA levels in an m6A-dependent manner [31]. *O*-GlcNAcylation reduces the translational activity of YTHDF1 and YTHDF3 by disrupting their interactions with mRNA translation factors and modulates stress granule dynamics to promote stress resilience [52]. *O*-GlcNAcylation of WTAP promotes glioblastoma malignant progression by stabilizing *LOXL2* mRNA via an m6A-IGF2BP2-dependent manner [53]. The methyltransferase domains of METTL3 (aa 357–580) and METTL14 (aa 111–456) are sufficient for METTL3/METTL14 complex formation and act cooperatively in RNA methylation [7]. Our findings reveal that *O*-GlcNAcylation does not affect METTL3 m6A methyltransferase activity or its interaction with METTL14. Moreover, we demonstrated that *O*-GlcNAcylation significantly increases METTL3 stability and binding to WTAP, leading to elevated m6A levels in hepatoma cells. We identified *MCM10* as an m6A target regulated by METTL3, with its mRNA m6A modification enhanced by *O*-GlcNAcylation. We demonstrated that METTL3 *O*-GlcNAcylation promotes HCC progression by stabilizing *MCM10* mRNA in an m6A-IGF2BP3-dependent manner. Our research elucidates, for the first time, the mechanism by which *O*-GlcNAcylation modulates m6A writer proteins and its specific contribution to tumorigenesis.

The therapeutic potential of targeting METTL3 in cancer is gaining traction, with inhibitors such as STM2457 and RSM3 showing promise in preclinical models. STM2457, a small-molecule inhibitor, effectively suppresses METTL3 methyltransferase activity [54]. Treatment with STM2457 suppressed growth and increased apoptosis in acute myeloid leukemia cells and a mouse model. The stapled peptide inhibitor RSM3 disturbs METTL3-METTL14 complex formation and induces its proteasomal degradation [55], significantly inhibiting tumor growth and RNA methylation in tumor models. Our research highlights the therapeutic potential of polypeptides targeting METTL3 *O*-GlcNAcylation to inhibit the carcinogenic capabilities of hepatoma cells and impede HCC progression in mouse models. Compared with small-molecule inhibitors, peptides offer versatile and effective target inhibition, particularly in disrupting protein–protein interactions. Considering their low risk of drug resistance development and low immunogenicity, therapeutic peptides show promising prospects for clinical application. However, due to species polymorphism and potential off-target effects of polypeptides, further exploration is necessary to assess their efficacy against human liver cancers.

Our study had some inherent limitations. First, *O*-GlcNAcylation is dynamic and reversible, and the regulatory balance of METTL3 *O*-GlcNAcylation in HCC requires further investigation. Second, the variability in m6A-modified RNA profiles among cell types and disease states highlights the need for comprehensive m6A-CLIP-sequencing analyses and expanded research in a broader range of cell lines to fully understand the regulatory effects of METTL3 *O*-GlcNAcylation on its targets. Third, O-GlcNAcylation drives immune evasion and tumor progression by regulating tumor-associated macrophage polarization [56, 57], while METTL3 modulates the tumor microenvironment through m6A-dependent mechanisms [58]. Elucidating the role of METTL3 O-GlcNAcylation in these processes may uncover new avenues for immunotherapy. Finally, although we established a link between METTL3 *O*-GlcNAcylation and the initiation of liver cancer, the extent of its influence on advanced HCC stages and its significance in the context of anti-cancer therapeutics remain to be determined.

In conclusion, we identified, for the first time, three sites on METTL3 for *O*-GlcNAc modification, which is key for its stability and interaction with WTAP. Our findings underscore the significance of the *O*-GlcNAcylation-METTL3-MCM10-IGF2BP3 regulatory axis in HCC development and highlight the potential of targeting this modification for therapeutic gain. As the field of RNA epigenetics continues to evolve, our study contributes to the growing body of knowledge on the role of PTMs in cancer, offering new insights into m6A epitranscriptome regulation and the development of targeted therapies.

## Materials and methods

Full details are available in Supplementary Material and Methods.

### Clinical specimens

HCC tumor tissues and adjacent non-cancerous tissue samples were obtained from patients undergoing surgery at the Second Affiliated Hospital of Chongqing Medical University who had no prior exposure to chemotherapy or radiation therapy. Informed consent was obtained from all participants.

### Plasmids and recombinant adenoviruses

The full-length cDNA of human *METTL3* (NM_019852.5) was PCR-amplified and ligated into a pSEB-3×Flag vector. C-terminal truncated (1–259 aa) and N-terminal truncated (260–580 aa) mutants were PCR-amplified and subcloned into pSEB-3×Flag. METTL3-T186A, METTL3-S192A, METTL3-S193A, and METTL3-T186A/S192A/S193A (3A) mutants were constructed using overlap PCR. The METTL3-WT-Flag and METTL3-3A-Flag mutants were subcloned into pAdTrack-TO4 (provided by Dr. Tong-Chuan He, University of Chicago, Chicago, IL USA). Ad-METTL3-WT-Flag and Ad-METTL3-3A-Flag were successfully expressed in HEK293 cells using the AdEasy system [59]. Primer sequences are listed in Supplementary Table S2. pLV3-CMV-USP5 (human)-3Myc-CopGFP-Puro was obtained from MiaoLingBio (Wuhan, China).

### Lentivirus-based RNA interference

The lentivirus-based vector pLL3.7 was used to construct short hairpin (sh)RNAs (provided by Prof. Bing Sun, Center for Excellence in Molecular Cell Science, Chinese Academy of Sciences, Shanghai, China). The shRNA sequences for the target genes are listed in Supplementary Table S2. Lentivirus was generated by cotransfecting pCMV-VSV-G, Δ8.9, and pLL3.7-shRNA into HEK293T cells using Lipo8000 (Beyotime, Shanghai, China), following the manufacturer’s instructions [60].

### In vivo ubiquitination assay

Huh-7 and PLC/PRF/5 cells were cotransfected with pCMV-His-Ub and indicated plasmids for 36 h and then treated with 10 μM MG132 (HY-13259; MCE), a proteasome inhibitor, for an additional 10 h. The cells were then lysed using buffer A (6 M guanidine-HCl, 0.1 M Na_2_HPO_4_/NaH_2_PO_4_, and 10 mM imidazole, pH 8.0), and sonicated in 10 two-second bursts. The lysates were mixed with Ni-NTA magnetic beads (Thermo Fisher Scientific, Waltham, MA, USA) at room temperature for 3 h. The His-tagged proteins were purified by washing the beads twice with buffer A, twice with a mixture of buffer A and buffer TI (1:3 ratio), and once with buffer TI (25 mM Tris-HCl, 20 mM imidazole, pH 6.8). The bound proteins were eluted by heating in 1× sodium dodecyl sulfate polyacrylamide gel electrophoresis (SDS-PAGE) loading buffer supplemented with 500 mM imidazole and subjected to immunoblot analysis with specific antibodies.

### Immunoprecipitation (IP)

Huh-7 cells were transfected with Flag-METTL3 (WT or 3A) and harvested using IP lysis buffer (50 mM Tris-HCl pH8.0, 150 mM NaCl, 5 mM EDTA, 0.5% Triton X-100, 0.1% sodium deoxycholate, protease, and phosphatase inhibitor cocktail (MCE). The lysates were incubated with anti-Flag M2 affinity gel (Cat. No. A2220, Sigma-Aldrich, USA). For Co-IP assays, Huh-7 cells were cotransfected with HA-tagged or Flag-tagged plasmids. At 48 h post-transfection, precleared cell lysates were incubated with anti-Flag or anti-HA antibodies overnight and then with protein A/G magnetic beads (MCE) for 4 h. Finally, the immunoprecipitates were immunoblotted with specific antibodies.

### Quantitative reverse transcription PCR (RT-qPCR)

Total RNA was extracted from hepatoma cells using TRIzol reagent (Invitrogen, Carlsbad, CA, USA) and reverse transcribed using a PrimeScript RT Reagent Kit with gDNA Eraser (RR047A; TaKaRa, Tokyo, Japan) according to the manufacturer’s instructions. qPCRs were run using SYBR Green qPCR Master Mix (1725121; Bio-Rad). The specific primers used are listed in Supplementary Table S2.

### Peptide synthesis

Cell-penetrating peptides, including CPPtat, CPPtat-M1, CPPtat-M2, CPPtat-M2 mut, CPPtat-WT, and CPPtat-3A were synthesized by GenScript Biotech Corp (Nanjing, China). Their sequences are listed in Supplementary Table S3. The polypeptides were dissolved in PBS and stored at –20 °C.

### Animal models

Four-week-old male BALB/c nude mice were randomly divided into four groups (*n* = 6 per group). MHCC-97H cells were infected with shMETTL3 lentivirus and then transfected with AdMETTL3-WT or AdMETTL3-3A. To establish a subcutaneous xenograft model, 2 × 10^6^ cells suspended in 100 μL of PBS were subcutaneously injected into the flanks of mice. Tumor diameter and volume (length × width^2^ × 0.5) were measured using calipers and calculated every 4 days. To establish a lung metastasis model, 2 × 10^6^ cells suspended in 100 µL of PBS were intravenously injected via the tail vein. At 6 weeks post-implantation, the mice were sacrificed, and lung tissues were harvested for hematoxylin and eosin staining.

A spontaneous HCC mouse model was established using the Sleeping Beauty transposase system. Five-week-old male C57BL/6J mice were randomly divided into three groups (*n* = 10 per group). 1 μg of pCMV(CAT)T7-SB100, 12.5 μg of pT3-myr-AKT-HA, and 12.5 μg of pT/Caggs-NRASV12 dissolved in 1.5 mL of saline was injected via the lateral tail vein. Mice were injected intraperitoneally with CPPtat, CPPtat-WT, or CPPtat-3A (100 mg/kg) every 5 days for a total of six times. The mice were euthanized, and their livers were harvested for further analysis.

### Statistical analysis

Data are presented as mean ± standard deviation (SD). Statistical analyses were performed using GraphPad Prism version 10.0 (GraphPad Software, San Diego, CA, USA). For comparisons of two groups, an unpaired Student’s *t*-test was employed, whereas one-way analysis of variance (ANOVA) followed by Tukey tests was utilized for comparing more than two groups. Spearman’s correlation coefficient (r) was used to assess linear correlation. Statistical significance was set to *P* < 0.05.

## Supplementary information

Supplementary information

Uncropped Western blots

## Data Availability

The data that support the findings of this study are available from the corresponding author upon reasonable request.

## References

[1] Fang F, Wang X, Li Z, Ni K, Xiong C. Epigenetic regulation of mRNA N6-methyladenosine modifications in mammalian gametogenesis. Mol Hum Reprod. 2021;27:gaab025. 33823008 10.1093/molehr/gaab025

[2] Linder B, Grozhik AV, Olarerin-George AO, Meydan C, Mason CE, Jaffrey SR. Single-nucleotide-resolution mapping of m6A and m6Am throughout the transcriptome. Nat Methods. 2015;12:767–72. 26121403 10.1038/nmeth.3453 PMC4487409

[3] Meyer KD, Saletore Y, Zumbo P, Elemento O, Mason CE, Jaffrey SR. Comprehensive analysis of mRNA methylation reveals enrichment in 3′ UTRs and near stop codons. Cell. 2012;149:1635–46. 22608085 10.1016/j.cell.2012.05.003 PMC3383396

[4] Liu J, Yue Y, Han D, Wang X, Fu Y, Zhang L, et al. A METTL3-METTL14 complex mediates mammalian nuclear RNA N6-adenosine methylation. Nat Chem Biol. 2014;10:93–95. 24316715 10.1038/nchembio.1432 PMC3911877

[5] Ping XL, Sun BF, Wang L, Xiao W, Yang X, Wang WJ, et al. Mammalian WTAP is a regulatory subunit of the RNA N6-methyladenosine methyltransferase. Cell Res. 2014;24:177–89. 24407421 10.1038/cr.2014.3 PMC3915904

[6] Oerum S, Meynier V, Catala M, Tisné C. A comprehensive review of m6A/m6Am RNA methyltransferase structures. Nucleic Acids Res. 2021;49:7239–55. 34023900 10.1093/nar/gkab378 PMC8287941

[7] Wang P, Doxtader KA, Nam Y. Structural basis for cooperative function of Mettl3 and Mettl14 methyltransferases. Mol Cell. 2016;63:306–17. 27373337 10.1016/j.molcel.2016.05.041 PMC4958592

[8] Huang J, Dong X, Gong Z, Qin LY, Yang S, Zhu YL, et al. Solution structure of the RNA recognition domain of METTL3-METTL14 N6-methyladenosine methyltransferase. Protein Cell. 2019;10:272–84. 29542011 10.1007/s13238-018-0518-7 PMC6418081

[9] Wang Q, Chen C, Ding Q, Zhao Y, Wang Z, Chen J, et al. METTL3-mediated m6A modification of HDGF mRNA promotes gastric cancer progression and has prognostic significance. Gut. 2020;69:1193–205. 31582403 10.1136/gutjnl-2019-319639

[10] Han J, Wang JZ, Yang X, Yu H, Zhou R, Lu HC, et al. METTL3 promote tumor proliferation of bladder cancer by accelerating pri-miR221/222 maturation in m6A-dependent manner. Mol Cancer. 2019;18:110. 31228940 10.1186/s12943-019-1036-9 PMC6588935

[11] Pan J, Liu F, Xiao X, Xu R, Dai L, Zhu M, et al. METTL3 promotes colorectal carcinoma progression by regulating the m6A-CRB3-Hippo axis. J Exp Clin Cancer Res. 2022;41:19. 35012593 10.1186/s13046-021-02227-8 PMC8744223

[12] Chen M, Wei L, Law CT, Tsang FH, Shen J, Cheng CL, et al. RNA N6-methyladenosine methyltransferase-like 3 promotes liver cancer progression through YTHDF2-dependent posttranscriptional silencing of SOCS2. Hepatology. 2018;67:2254–70. 29171881 10.1002/hep.29683

[13] Xu Y, Lv D, Yan C, Su H, Zhang X, Shi Y, et al. METTL3 promotes lung adenocarcinoma tumor growth and inhibits ferroptosis by stabilizing SLC7A11 m6A modification. Cancer Cell Int. 2022;22:11. 34996469 10.1186/s12935-021-02433-6 PMC8742440

[14] Xiao Z, Wang S, Tian Y, Lv W, Sheng H, Zhan M, et al. METTL3-mediated m6A methylation orchestrates mRNA stability and dsRNA contents to equilibrate γδ T1 and γδ T17 cells. Cell Rep. 2023;42:112684. 37355989 10.1016/j.celrep.2023.112684

[15] Wang X, Lu Z, Gomez A, Hon GC, Yue Y, Han D, et al. N6-methyladenosine-dependent regulation of messenger RNA stability. Nature. 2014;505:117–20. 24284625 10.1038/nature12730 PMC3877715

[16] Barbieri I, Tzelepis K, Pandolfini L, Shi J, Millán-Zambrano G, Robson SC, et al. Promoter-bound METTL3 maintains myeloid leukaemia by m6A-dependent translation control. Nature. 2017;552:126–31. 29186125 10.1038/nature24678 PMC6217924

[17] Lesbirel S, Viphakone N, Parker M, Parker J, Heath C, Sudbery I, et al. The m6A-methylase complex recruits TREX and regulates mRNA export. Sci Rep. 2018;8:13827. 30218090 10.1038/s41598-018-32310-8 PMC6138711

[18] Xu H, Wang H, Zhao W, Fu S, Li Y, Ni W, et al. SUMO1 modification of methyltransferase-like 3 promotes tumor progression via regulating Snail mRNA homeostasis in hepatocellular carcinoma. Theranostics. 2020;10:5671–86. 32483411 10.7150/thno.42539 PMC7254988

[19] Li Y, He X, Lu X, Gong Z, Li Q, Zhang L, et al. METTL3 acetylation impedes cancer metastasis via fine-tuning its nuclear and cytosolic functions. Nat Commun. 2022;13:6350. 36289222 10.1038/s41467-022-34209-5 PMC9605963

[20] Xiong J, He J, Zhu J, Pan J, Liao W, Ye H, et al. Lactylation-driven METTL3-mediated RNA m6A modification promotes immunosuppression of tumor-infiltrating myeloid cells. Mol Cell. 2022;82:1660–1677.e10. 35320754 10.1016/j.molcel.2022.02.033

[21] Sun HL, Zhu AC, Gao Y, Terajima H, Fei Q, Liu S, et al. Stabilization of ERK-phosphorylated METTL3 by USP5 increases m6A methylation. Mol Cell. 2020;80:633–647.e7. 33217317 10.1016/j.molcel.2020.10.026 PMC7720844

[22] Xie X, Wu Q, Zhang K, Liu Y, Zhang N, Chen Q, et al. O-GlcNAc modification regulates MTA1 transcriptional activity during breast cancer cell genotoxic adaptation. Biochim Biophys Acta Gen Subj. 2021;1865:129930. 34019948 10.1016/j.bbagen.2021.129930

[23] Zhang W, Liu T, Dong H, Bai H, Tian F, Shi Z, et al. Synthesis of a highly azide-reactive and thermosensitive biofunctional reagent for efficient enrichment and large-scale identification of O-GlcNAc proteins by mass spectrometry. Anal Chem. 2017;89:5810–7. 28510447 10.1021/acs.analchem.6b04960

[24] Hart GW, Housley MP, Slawson C. Cycling of O-linked beta-N-acetylglucosamine on nucleocytoplasmic proteins. Nature. 2007;446:1017–22. 17460662 10.1038/nature05815

[25] Chu Y, Jiang M, Wu N, Xu B, Li W, Liu H, et al. O-GlcNAcylation of SIX1 enhances its stability and promotes hepatocellular carcinoma proliferation. Theranostics. 2020;10:9830–42. 32863962 10.7150/thno.45161 PMC7449927

[26] Wang Y, Liu J, Jin X, Zhang D, Li D, Hao F, et al. O-GlcNAcylation destabilizes the active tetrameric PKM2 to promote the Warburg effect. Proc Natl Acad Sci USA. 2017;114:13732–7. 29229835 10.1073/pnas.1704145115 PMC5748163

[27] Zhang Y, Zhou S, Kai Y, Zhang YQ, Peng C, Li Z, et al. O-GlcNAcylation of MITF regulates its activity and CDK4/6 inhibitor resistance in breast cancer. Nat Commun. 2024;15:5597. 38961064 10.1038/s41467-024-49875-w PMC11222436

[28] Li J, Ahmad M, Sang L, Zhan Y, Wang Y, Yan Y, et al. O-GlcNAcylation promotes the cytosolic localization of the m6A reader YTHDF1 and colorectal cancer tumorigenesis. J Biol Chem. 2023;299:104738. 37086786 10.1016/j.jbc.2023.104738 PMC10208891

[29] Xu C, Pan X, Wang D, Guan Y, Yang W, Chen X, et al. O-GlcNAcylation of Raptor transduces glucose signals to mTORC1. Mol Cell. 2023;83:3027–3040.e11. 37541260 10.1016/j.molcel.2023.07.011

[30] Zhang J, Xun M, Li C, Chen Y. The O-GlcNAcylation and its promotion to hepatocellular carcinoma. Biochim Biophys Acta Rev Cancer. 2022;1877:188806. 36152903 10.1016/j.bbcan.2022.188806

[31] Yang Y, Yan Y, Yin J, Tang N, Wang K, Huang L, et al. O-GlcNAcylation of YTHDF2 promotes HBV-related hepatocellular carcinoma progression in an N6-methyladenosine-dependent manner. Signal Transduct Target Ther. 2023;8:63. 36765030 10.1038/s41392-023-01316-8 PMC9918532

[32] Liu S, Zhuo L, Wang J, Zhang Q, Li Q, Li G, et al. METTL3 plays multiple functions in biological processes. Am J Cancer Res. 2020;10:1631–46. 32642280 PMC7339281

[33] Yang X, Qian K. Protein O-GlcNAcylation: emerging mechanisms and functions. Nat Rev Mol Cell Biol. 2017;18:452–65. 28488703 10.1038/nrm.2017.22 PMC5667541

[34] Dewson G, Eichhorn PJA, Komander D. Deubiquitinases in cancer. Nat Rev Cancer. 2023;23:842–62. 37935888 10.1038/s41568-023-00633-y

[35] Cruz Walma DA, Chen Z, Bullock AN, Yamada KM. Ubiquitin ligases: guardians of mammalian development. Nat Rev Mol Cell Biol. 2022;23:350–67. 35079164 10.1038/s41580-021-00448-5

[36] Ahmed SMQ, Sasikumar J, Laha S, Das SP. Multifaceted role of the DNA replication protein MCM10 in maintaining genome stability and its implication in human diseases. Cancer Metastasis Rev. 2024;43:1353–71. 39240414 10.1007/s10555-024-10209-3

[37] Yang WD, Wang L. MCM10 facilitates the invaded/migrated potentials of breast cancer cells via Wnt/β-catenin signaling and is positively interlinked with poor prognosis in breast carcinoma. J Biochem Mol Toxicol. 2019;33:e22330. 30990947 10.1002/jbt.22330

[38] Tian J, Lu Z, Niu S, Zhang S, Ying P, Wang L, et al. Aberrant MCM10 SUMOylation induces genomic instability mediated by a genetic variant associated with survival of esophageal squamous cell carcinoma. Clin Transl Med. 2021;11:e485. 34185429 10.1002/ctm2.485 PMC8236122

[39] Chen YR, Li YT, Wang MQ, Zhu SL. Prognostic significance and function of MCM10 in human hepatocellular carcinoma. Future Oncol. 2021;17:4457–70. 34350781 10.2217/fon-2021-0225

[40] Huang H, Weng H, Sun W, Qin X, Shi H, Wu H, et al. Recognition of RNA N6-methyladenosine by IGF2BP proteins enhances mRNA stability and translation. Nat Cell Biol. 2018;20:285–95. 29476152 10.1038/s41556-018-0045-z PMC5826585

[41] Bu L, Zhang Z, Chen J, Fan Y, Guo J, Su Y, et al. High-fat diet promotes liver tumorigenesis via palmitoylation and activation of AKT. Gut. 2024;73:1156–68. 38191266 10.1136/gutjnl-2023-330826

[42] He L, Li H, Wu A, Peng Y, Shu G, Yin G. Functions of N6-methyladenosine and its role in cancer. Mol Cancer. 2019;18:176. 31801551 10.1186/s12943-019-1109-9 PMC6892141

[43] Lin Y, Lin P, Lu Y, Zheng J, Zheng Y, Huang X, et al. Post-translational modifications of RNA-modifying proteins in cellular dynamics and disease progression. Adv Sci. 2024;11:e2406318. 10.1002/advs.202406318 PMC11600222 39377984

[44] He XF, Hu X, Wen GJ, Wang Z, Lin WJ. O-GlcNAcylation in cancer development and immunotherapy. Cancer Lett. 2023;566:216258. 37279852 10.1016/j.canlet.2023.216258

[45] Zhang D, Qi Y, Inuzuka H, Liu J, Wei W. O-GlcNAcylation in tumorigenesis and its implications for cancer therapy. J Biol Chem. 2024;300:107709. 39178944 10.1016/j.jbc.2024.107709 PMC11417186

[46] Ruan HB, Nie Y, Yang X. Regulation of protein degradation by O-GlcNAcylation: crosstalk with ubiquitination. Mol Cell Proteom. 2013;12:3489–97. 10.1074/mcp.R113.029751 PMC3861702 23824911

[47] Sun K, Zhi Y, Ren W, Li S, Zheng J, Gao L, et al. Crosstalk between O-GlcNAcylation and ubiquitination: a novel strategy for overcoming cancer therapeutic resistance. Exp Hematol Oncol. 2024;13:107. 39487556 10.1186/s40164-024-00569-5 PMC11529444

[48] Zhang F, Su K, Yang X, Bowe DB, Paterson AJ, Kudlow JE. O-GlcNAc modification is an endogenous inhibitor of the proteasome. Cell. 2003;115:715–25. 14675536 10.1016/s0092-8674(03)00974-7

[49] Song T, Zou Q, Yan Y, Lv S, Li N, Zhao X, et al. DOT1L O-GlcNAcylation promotes its protein stability and MLL-fusion leukemia cell proliferation. Cell Rep. 2021;36:109739. 34551297 10.1016/j.celrep.2021.109739

[50] Feng Z, Yin J, Zhang Z, Chen Z, Huang L, Tang N, et al. O-GlcNAcylation of E3 ubiquitin ligase SKP2 promotes hepatocellular carcinoma proliferation. Oncogene. 2024;43:1149–59. 38396292 10.1038/s41388-024-02977-7

[51] Yang WH, Kim JE, Nam HW, Ju JW, Kim HS, Kim YS, et al. Modification of p53 with O-linked N-acetylglucosamine regulates p53 activity and stability. Nat Cell Biol. 2006;8:1074–83. 16964247 10.1038/ncb1470

[52] Chen Y, Wan R, Zou Z, Lao L, Shao G, Zheng Y, et al. O-GlcNAcylation determines the translational regulation and phase separation of YTHDF proteins. Nat Cell Biol. 2023;25:1676–90. 37945829 10.1038/s41556-023-01258-x PMC12060179

[53] Qiu J, Zhao R, Ma C, Wang Q, Li B, Qi Y. et al. O-GlcNAcylation stabilized WTAP promotes GBM malignant progression in an N6-methyladenosine-dependent manner. Neuro Oncol. 7844;27:900–15. 39671515 10.1093/neuonc/noae268 PMC12083224

[54] Yankova E, Blackaby W, Albertella M, Rak J, De Braekeleer E, Tsagkogeorga G, et al. Small-molecule inhibition of METTL3 as a strategy against myeloid leukaemia. Nature. 2021;593:597–601. 33902106 10.1038/s41586-021-03536-w PMC7613134

[55] Li Z, Feng Y, Han H, Jiang X, Chen W, Ma X, et al. A stapled peptide inhibitor targeting the binding interface of N6-adenosine-methyltransferase subunits METTL3 and METTL14 for cancer therapy. Angew Chem Int Ed Engl. 2024;63:e202402611. 38607929 10.1002/anie.202402611

[56] Lee JB, Pyo K-H, Kim HR. Role and function of O-GlcNAcylation in cancer. Cancers. 2021;13:5365. 34771527 10.3390/cancers13215365 PMC8582477

[57] Ciraku L, Esquea EM, Reginato MJ. O-GlcNAcylation regulation of cellular signaling in cancer. Cell Signal. 2022;90:110201. 34800629 10.1016/j.cellsig.2021.110201 PMC8712408

[58] Su W, Che L, Liao W, Huang H. The RNA m6A writer METTL3 in tumor microenvironment: emerging roles and therapeutic implications. Front Immunol. 2024;15:1335774. 38322265 10.3389/fimmu.2024.1335774 PMC10845340

[59] Luo J, Deng ZL, Luo X, Tang N, Song WX, Chen J, et al. A protocol for rapid generation of recombinant adenoviruses using the AdEasy system. Nat Protoc. 2007;2:1236–47. 17546019 10.1038/nprot.2007.135

[60] Kutner RH, Zhang XY, Reiser J. Production, concentration and titration of pseudotyped HIV-1-based lentiviral vectors. Nat Protoc. 2009;4:495–505. 19300443 10.1038/nprot.2009.22

