# Supplementary material for: O-GlcNAcylation of METTL3 drives hepatocellular carcinoma progression by upregulating MCM10 expression in an m6A-IGF2BP3-dependent manner
Source: Cell Death Dis. 2025 Jul 12;16(1):518. doi: 10.1038/s41419-025-07844-1 (PMC12255776; doi:10.1038/s41419-025-07844-1)
Supplement: Supplementary file 1 — Supplementary information [file 41419_2025_7844_MOESM1_ESM.pdf]

Supplementary information

O-GlcNAcylation of METTL3 Drives Hepatocellular Carcinoma Progression by Upregulating MCM10 Expression in an m6A-IGF2BP3-Dependent Manner

Zhen Chen[1#], Jiaxin Yin[1#], Zhongqi Feng[1#], Yanlai Zhang[1], Li Liang[1], Xiaojun Wang[2*], Kai Wang[1*], Ni Tang[1*]

[1]Department of Infectious Diseases, Key Laboratory of Molecular Biology for Infectious Diseases (Ministry of Education), Institute for Viral Hepatitis, the Second Affiliated Hospital, Chongqing Medical University, Chongqing 400016, China

[2]Institute of Hepatobiliary Surgery, Southwest Hospital, Third Military Medical University (Army Medical University), Chongqing 400038, China

This PDF file includes:

Supplementary Materials and Methods

Supplementary Figures and Figure Legends

Supplementary Table S1-S4

Supplementary Materials and Methods

Cell Culture

PLC/PRF/5 cells were purchased from the American Type Culture Collection (Manassas, VA, USA). Huh-7 and MHCC-97H cells were obtained from the Cell Bank of Chinese Academy of Sciences (Shanghai, China). HEK293 and HEK293T cells were stored in our laboratory. All cells used in experiments were maintained in Dulbecco's modified Eagle's medium supplemented with 10% fetal bovine serum (FBS; Natocor, Cordoba, Argentina), 100 units/mL penicillin, and 100 μg/mL streptomycin (MedChemExpress [MCE], Monmouth Junction, NJ, USA).

Western Blotting

Cells and liver tissue lysates were denatured in 6× SDS-PAGE loading buffer. The proteins were separated by 10% SDS-PAGE and transferred to polyvinylidene difluoride membranes (IPVH00010; Merck Millipore, Billerica, MA, USA). After blocking with 5% non-fat milk, the membranes were incubated with the indicated primary antibodies (Supplementary Table S3). Subsequently, the membranes were incubated with HRP-linked secondary antibodies (Bio-Rad) and visualized using Clarity Western ECL Substrate (Bio-Rad). Densitometric analysis was performed using ImageJ, with target protein expression normalized to β-actin (loading control) or Input lysate (for immunoprecipitation and sWGA pull-down assays). Uncropped western blots are included in the Supplementary Materials.

GST Pull-down Assay

Purified recombinant GST-OGT and His-METTL3 proteins were incubated with glutathione agarose beads (GE Healthcare, Piscataway, NJ, USA) at 4 °C for 3 h. The beads were washed five times with wash buffer (137 mM NaCl, 2.7 mM KCl, 10 mM Na_2HPO_4, 2 mM KH_2PO_4, and 0.5% Triton X-100) and boiled in 2× SDS loading buffer. The samples were subjected to Coomassie blue staining and immunoblotting.

Succinylated Wheat Germ Agglutinin (sWGA) Pull-Down Assay

Hepatic cells and liver tissues were lysed in Lysis 125 buffer containing 50 mM Tris pH 7.4, 5 mM EDTA, 125 mM NaCl, 0.1% NP-40, and 1× protease inhibitor cocktail. The supernatant was denatured in glycoprotein-denaturing buffer and digested with

PNGase F (P0704S; New England Biolabs, Ipswich, MA, USA) to remove N-linked glycoproteins. Pre-cleared lysates were incubated with sWGA-conjugated agarose bead (AL-1023S; Vector Laboratories, Burlingame, CA, USA) at 4 °C overnight. The precipitated complexes were washed and immunoblotted with anti-Flag or anti-METTL3 antibodies.

***In Vitro O*-GlcNAcylation Assays**

Recombinant His-METTL3 (1–580 aa) was purified using a His-tag Protein Purification Kit (P2226; Beyotime) according to the manual. Recombinant glutathione S-transferase (GST)-OGT (aa 313–1031) was purified using a GST-tag Protein Purification Kit (P2262; Beyotime). His-METTL3 protein (2 µg) was incubated with 1 µg of GST-OGT in a 50 µL reaction volume (50 mM Tris-HCl, 12.5 mM $MgCl_2$, 2 mM UDP-GlcNAc 1 mM dithiothreitol [DTT], pH 7.5) at 37 °C for 4 h. The samples were analyzed using Coomassie staining and immunoblotting with the indicated antibodies.

Click-iT *O*-GlcNAc Enzymatic Labeling Assay

O-GlcNAcylation of METTL3 was analyzed using an enzymatic labeling approach, as previously reported [1]. In brief, 400 µg of cellular lysate was subjected to *O*-GlcNAc labeling using an Invitrogen Click-iT *O*-GlcNAc Enzyme Labeling Kit (C33368; Thermo Fisher Scientific, Waltham, MA, USA) per the manufacturer's instructions. Enzymatically labeled proteins were conjugated with an alkyne-biotin compound using an Invitrogen Click-iT Protein Analysis Detection Kit (C33372; Thermo Fisher Scientific). Biotinylated proteins were precipitated with streptomycin-linked globular reagent (53113; Thermo Fisher Scientific) and eluted in 1× loading buffer under boiling. *O*-GlcNAcylation of METTL3 was detected using immunoblotting.

METTL3 *O*-GlcNAcylation Site Mapping

HCC was induced in mice through cotreatment with diethylnitrosamine (75 mg/kg) and CCl_4 (2 mL/kg, twice weekly for 12 weeks). HCC tissue samples were sent to Shanghai Applied Protein Technology (Shanghai, China) for O-GlcNAcylation 4D label-free quantitative proteomics, as previously described [2].

Cell Proliferation and Colony Formation Assays

Cells (2×10^3) were seeded in 96-well plates and cultured for 5 days. Cell proliferation was measured using a Cell Counting Kit-8 (CCK-8; TargetMol, Boston, USA) at indicated times. For the colony formation assay, 1×10^3 cells were seeded in 6-well plates and cultured for 14 days. Then, colonies were stained with 0.04% crystal violet and photographed.

Wound-healing Assay

Cells (2×10^4) were seeded in 96-well plates, and wounds were created on the cell surface using a WoundMaker (Essen Bioscience, Ann Arbor, MI, USA). The wound areas were photographed using a IncuCyte ZOOM Live-Cell Imaging system (Essen BioScience) at indicated times.

Transwell Migration Assay

Cell migration was assessed using Transwell units with a polycarbonate filter (BD Falcon, USA). Cells (2×10^4) suspended in serum-free medium were transferred into the upper chamber, whereas the lower chamber was filled with medium containing 10% FBS. After 24 h, the cells were fixed with 4% formaldehyde and stained with crystal violet. The migrated cells were counted (3 random 200× fields per well) under an Axio Imager A2 (ZEISS, Oberkochen, Germany).

Immunohistochemical Staining (IHC)

Liver tissues were fixed in 4% paraformaldehyde, embedded in paraffin, and sectioned following standard procedures. Tissue sections were incubated with the indicated primary antibodies at 4 °C overnight and then with secondary anti-mouse or anti-rabbit IgG antibodies (ZSGB-BIO, Beijing, China). The sections were stained with 3,3′-diaminobenzidine (ZSGB-BIO) and scanned with a Pannoramic Scan 250 Flash or MIDI system. Images were captured with Pannoramic Viewer 1.15.2 (3DHistech, Budapest, Hungary).

Immunofluorescence Staining

Cells were fixed in 4% paraformaldehyde for 30 min, permeabilized with 0.5% Triton X-100 for 20 min, and incubated with specific primary antibodies overnight at 4 °C. Subsequently, the cells were incubated with fluorescence-labeled secondary

antibodies (ZSGB-BIO) and treated with 1 μg/mL 4′,6-diamidine-2′-phenylindole dihydrochloride (DAPI; Cat. No. 10236276001; Roche Diagnostics GmbH) for nuclear staining. Images were acquired using a Leica confocal microscope (Leica TCS SP8; Leica Microsystems, Wetzlar, Germany).

m6A Dot Blot Assay

mRNA was isolated from total RNA using a TIANSeq mRNA Capture Kit (TIANGEN Biotech, Beijing, China). After denaturation at 95 °C for 5 min and followed by immediately chilling on ice, 400, 200, and 100 ng mRNA were spotted onto a Hybond-N+ membrane (Roche Diagnostics GmbH, Mannheim, Germany). The membrane was crosslinked using a UV cross linker and washed with phosphate-buffered saline (PBS) with Tween-20, stained with 0.02% ethylene blue (Solarbio, Beijing, China), and scanned to determine the total amount of input mRNA. After blocking with 5% non-fat milk, the membrane was incubated with m6A antibody (1:1000, cat. No. ab208577; Abcam, Cambridge, USA) at 4 °C overnight. Finally, the membrane was hatched with horseradish peroxidase (HRP)-conjugated anti-mouse IgG for 1 h and visualized using an imaging system (Bio-Rad, Hercules, CA, USA).

***In vitro* m6A Methyltransferase Activity Assay**

Huh-7 and PLC/PRF/5 cells were infected with Ad-METTL3-Flag (WT or 3A mutant). The infected cells were harvested and lysed. The lysates were incubated with anti-FLAG M2 agarose beads (Sigma-Aldrich) at 4 °C overnight. The Flag-METTL3 proteins were eluted from the beads through incubation in elution buffer (10 mM Tris-HCl pH 7.4, 50 mM HEPES, 150 mM NaCl, 1 mg/mL Flag peptide, 1× protease inhibitor cocktail) at 4 °C for 2 h. Immunoprecipitates were immunoblotted with anti-Flag for normalization. METTL3 catalytic activity was measured by using an Epigenase m6A Methylase Activity/Inhibition Assay Kit (Cat. No. P9019; EpiGentek, Farmingdale, NY, USA) following the manufacturer's instructions.

Cytoplasmic and Nuclear Protein Extraction

Cytoplasmic and nuclear proteins were extracted using a Nuclear and Cytoplasmic Protein Extraction Kit (Cat. No. P0028; Beyotime) according to the manufacturer's instructions. Histone H3 and β-tubulin were used as controls for the nuclear and

cytosolic fractions, respectively.

Protein Stability Assay

For the endogenous METTL3 protein stability assay, Huh-7 and PLC/PRF/5 cells were treated with 25 μM Thiamet G (TMG; HY-12588; MCE), a selective *O*-GlcNAcase inhibitor, for 12 h or infected with shOGT lentivirus for 48 h. To detect the stability of exogenous METTL3 (WT or 3A) protein, Huh-7 and PLC/PRF/5 cells were infected with Ad-METTL3-Flag (WT or 3A mutant) and then treated with 100 μM cycloheximide (CHX; MCE), a protein synthesis inhibitor. Cell lysates were harvested at 0, 12, 36, and 48 h, and proteins were detected using specific antibodies.

mRNA Stability Assay

Cells were treated with 5 μg/mL actinomycin D (S8964; Selleck, TX, USA) for 0, 2, 4, 6 h, and total RNA was isolated at indicated times using TRIzol reagent. mRNA levels were quantified using RT-qPCR with the specific primers listed in Supplementary Table S1. Data were normalized to actin used as the endogenous control.

Methylated RNA Immunoprecipitation-qPCR (MeRIP-qPCR)

MeRIP-qPCR was conducted as previously described, with minor adjustments [3]. Total RNA was isolated using TRIzol reagent, and mRNA was enriched using a TIANSeq mRNA Capture Kit. The mRNA was immunoprecipitated with anti-m6A antibody or anti-mouse IgG in IP lysis buffer (10 mM Tris-HCl, pH 7.4, 150 mM NaCl, 0.1% NP-40, and 100 U/mL RNase inhibitor) containing 50 μL of protein A/G magnetic beads at 4 °C overnight. The m6A-enriched mRNA was extracted using a phenol:chloroform mixture followed by ethanol precipitation. Changes in m6A methylation of specific target genes were analyzed using qPCR. The primer sequences are listed in Supplementary Table S1.

RNA immunoprecipitation (RIP)

After ultraviolet crosslinking, cells were lysed with RIP lysis buffer (150 mM KCl, 0.5% NP-40, 25 mM Tris-HCl PH 7.4, 0.5 mM DTT, 5 mM EDTA, protease inhibitor, and 100 U/mL RNase inhibitor). The RIP lysates were incubated with 50 μL of

protein A/G magnetic beads coated with rabbit anti-IGF2BP3 antibody or normal rabbit IgG under orbital rotation at 4 °C overnight. The immunoprecipitated RNAs were reverse transcribed into cDNA for qPCR analysis. The IP enrichment ratio of a transcript was calculated as the ratio of its amount in IP to that in the input.

Supplementary References

1. Chen L, Zhou Q, Zhang P, Tan W, Li Y, Xu Z, et al. Direct stimulation of de novo nucleotide synthesis by O-GlcNAcylation. Nat Chem Biol 2024; 20: 19–29.
2. Zhou P, Chang WY, Gong DA, Xia J, Chen W, Huang LY, et al. High dietary fructose promotes hepatocellular carcinoma progression by enhancing O-GlcNAcylation via microbiota-derived acetate. Cell Metab 2023; 35: 1961-1975.e6.
3. Wan W, Ao X, Chen Q, Yu Y, Ao L, Xing W, et al. METTL3/IGF2BP3 axis inhibits tumor immune surveillance by upregulating N6-methyladenosine modification of PD-L1 mRNA in breast cancer. Mol Cancer 2022; 21: 60.
4. Du Y, Hou G, Zhang H, Dou J, He J, Guo Y, et al. SUMOylation of the m6A-RNA methyltransferase METTL3 modulates its function. Nucleic Acids Res 2018; 46: 5195–5208.
5. Tian J, Lu Z, Niu S, Zhang S, Ying P, Wang L, et al. Aberrant MCM10 SUMOylation induces genomic instability mediated by a genetic variant associated with survival of esophageal squamous cell carcinoma. Clin Transl Med 2021; 11: e485.

Supplementary Figures and Figure Legends

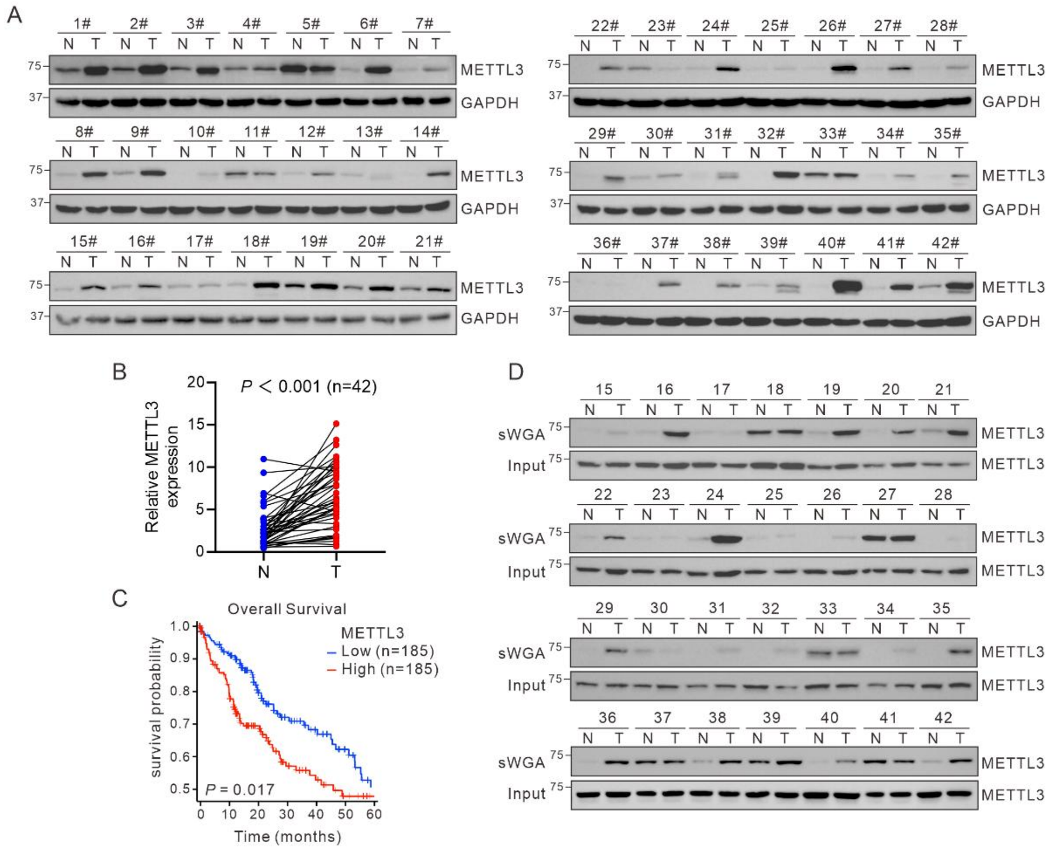

Fig. S1 Upregulation of METTL3 and O-GlcNAcylation in clinical samples.

A, B The protein levels of METTL3 in 42 paired human HCCs were detected by the western blot analysis (A) and quantified by Image J (B). **C** Kaplan–Meier survival analysis of overall survival rate in HCC patients based on METTL3 expression levels. *$P < 0.05$, log-rank test. **D** The O-GlcNAcylation levels of METTL3 in paired HCC tissues.

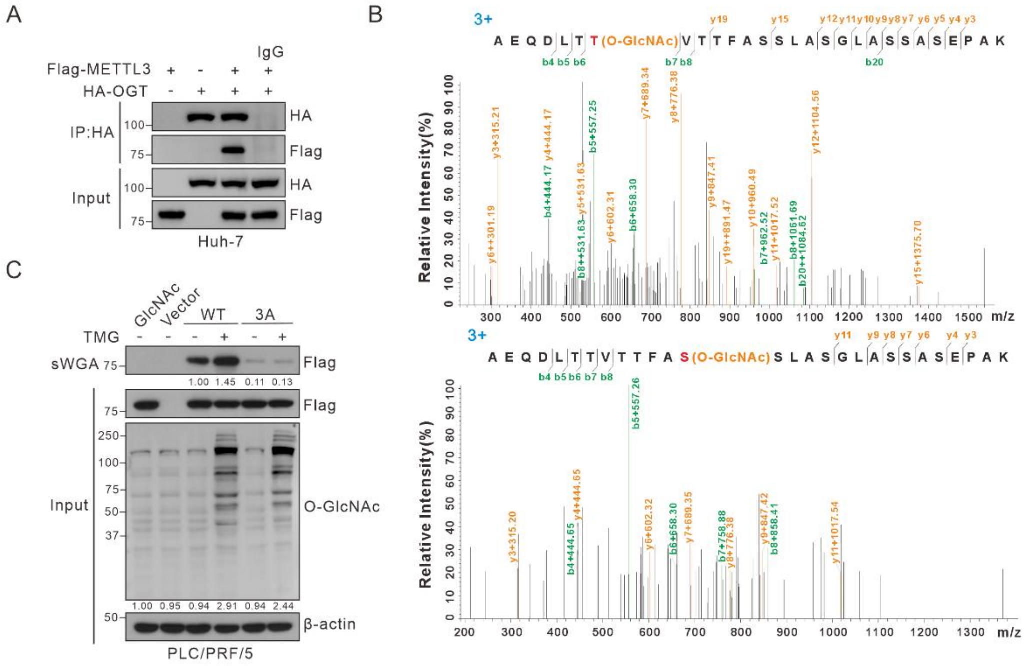

Fig. S2 OGT O-GlcNAcylates METTL3 at Thr186/Ser192/Ser193.

A Co-IP assays were performed to observe the interaction between METTL3 and OGT in Huh-7 cells. **B** LC-MS identified Thr186 and Ser192 as the METTL3 O-GlcNAcylation sites, with the O-GlcNAc-modified peptides being demonstrated. **C** sWGA pull-down assays were conducted to confirm METTL3 O-GlcNAcylation sites in PLC/PRF/5 hepatoma cells.

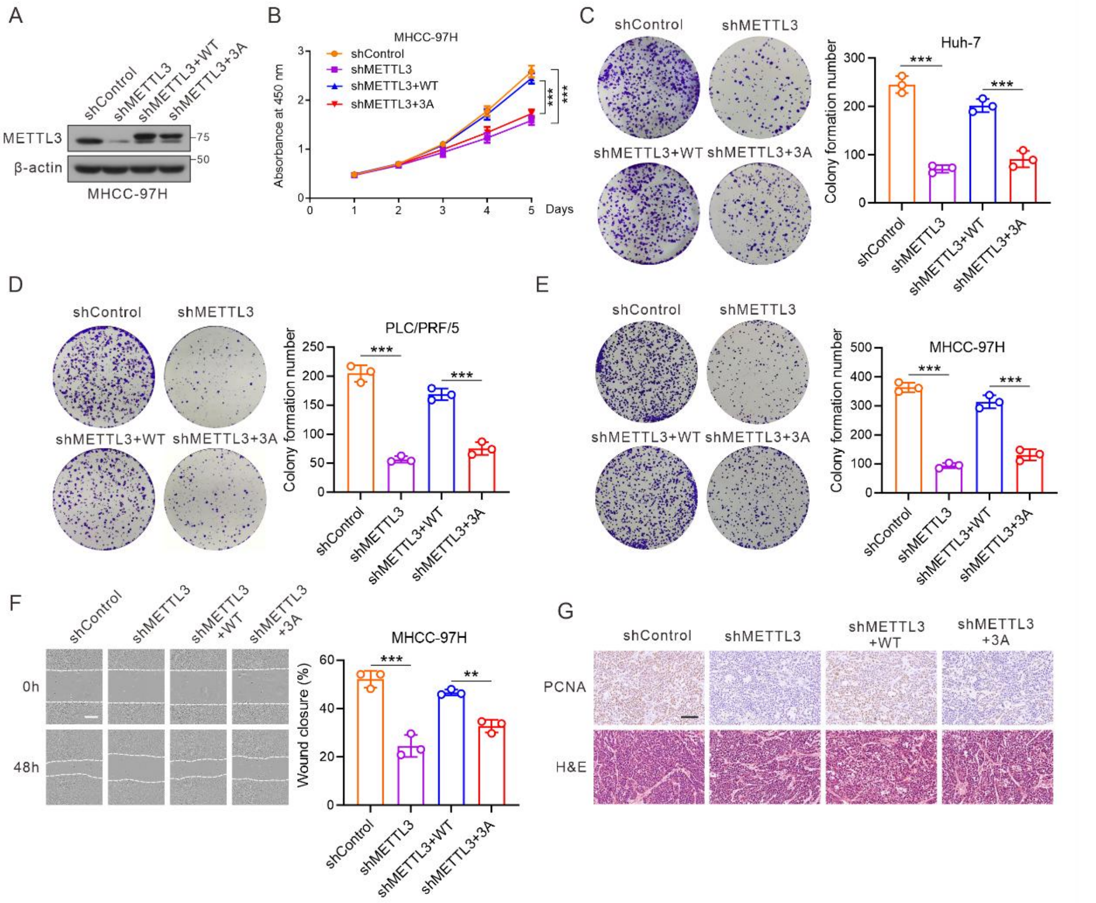

Fig. S3 O-GlcNAcylation of METTL3 promotes HCC progression *in vitro* and *in vivo*.

Hepatoma cells were transfected with shMETTL3 lentivirus and subsequently infected with adenoviruses expressing Flag-METTL3 (WT or 3A). **A** Immunoblotting of METTL3 in MHCC-97H cells. **B** CCK-8 assays to examine cell proliferation ability of MHCC-97H cells. **C–E** Colony formation capacity of Huh-7 cells (C), PLC/PRF/5 cells (D), and MHCC-97H cells (E). **F** Wound-healing assays to observe migration capacity of MHCC-97H cells. Scale bar: 200 μm. For B–F, data were shown as mean ± SD from three independent experiments. One-way ANOVA followed by Tukey's test, $^{**}P < 0.01$, $^{***}P < 0.001$. **G** Immunohistochemical (IHC) staining of PCNA in subcutaneous implantation tumors. Scale bar: 100 μm.

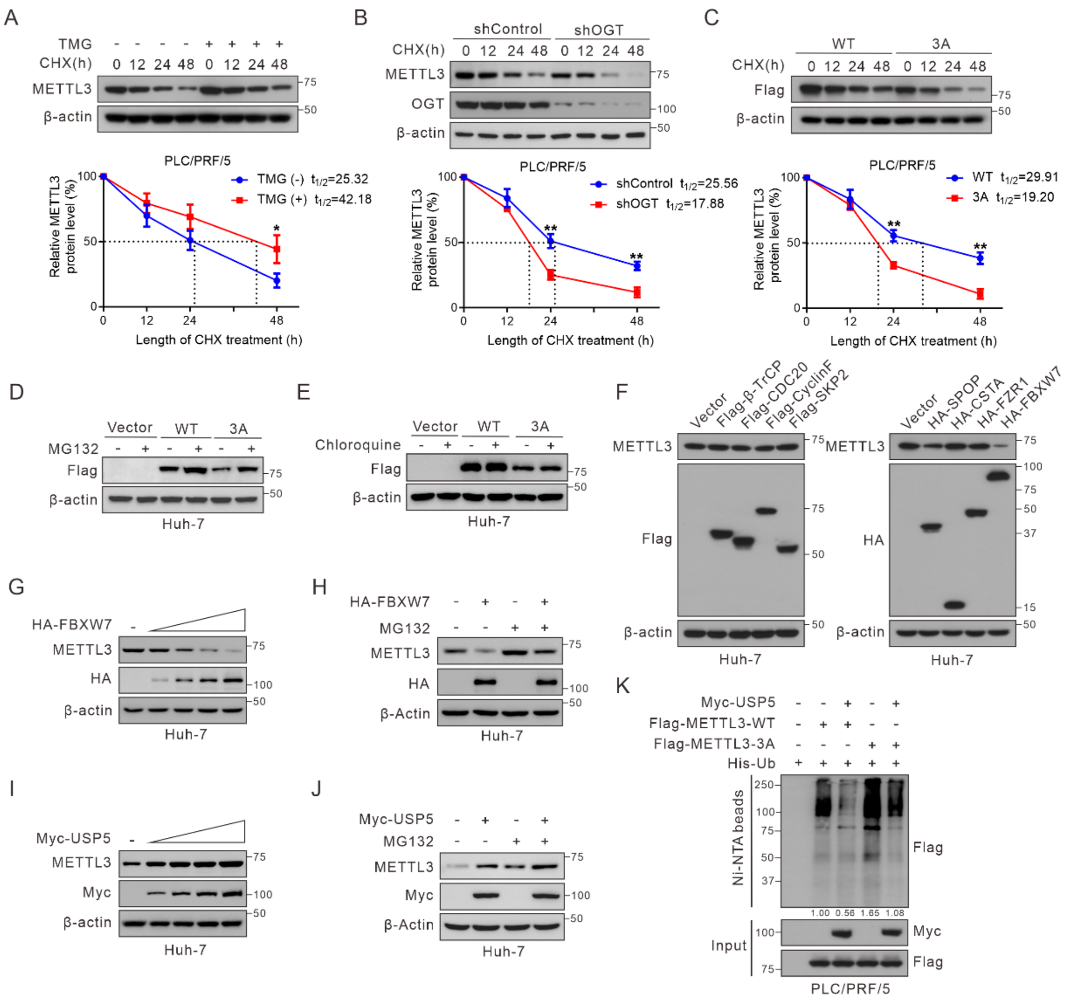

Fig. S4 O-GlcNAcylation of METTL3 enhances its stability through ubiquitin-proteasome pathway.

A, B Half-life and quantitative analysis of METTL3. PLC/PRF/5 cells were treated with 25 μM TMG (A) or OGT shRNA lentivirus (B), followed by addition of 100 μM CHX for the indicated times. **C** Half-life of Flag-METTL3 and quantitative analysis in PLC/PRF/5 cells. Data in panels A–C were analyzed using an unpaired Student's *t*-test. $*P < 0.05$, $**P < 0.01$. **D, E** Huh-7 cells were infected with Flag-METTL3 (WT or 3A mutant), followed by MG132 (D) or Chloroquine (E) treatment, then cell lysates were subjected to immunoblotting. **F** Huh-7 cells were transfected with the indicated plasmids for 48 h and cell lysates were collected to immunoblotting to monitor changes in METTL3 protein levels. **G, I** Huh-7 cells were transfected with FBXW7 (G) or USP5 (I) in a dose-dependent manner to observe alterations in METTL3 protein levels. **H**

FBXW7 leads to METTL3 downregulation via ubiquitin-proteasome pathway, **J** while USP5 up-regulates METTL3 protein levels through the same pathway. **K** USP5 decreases the poly-ubiquitination level of METTL3 in PLC/PRF/5 cells.

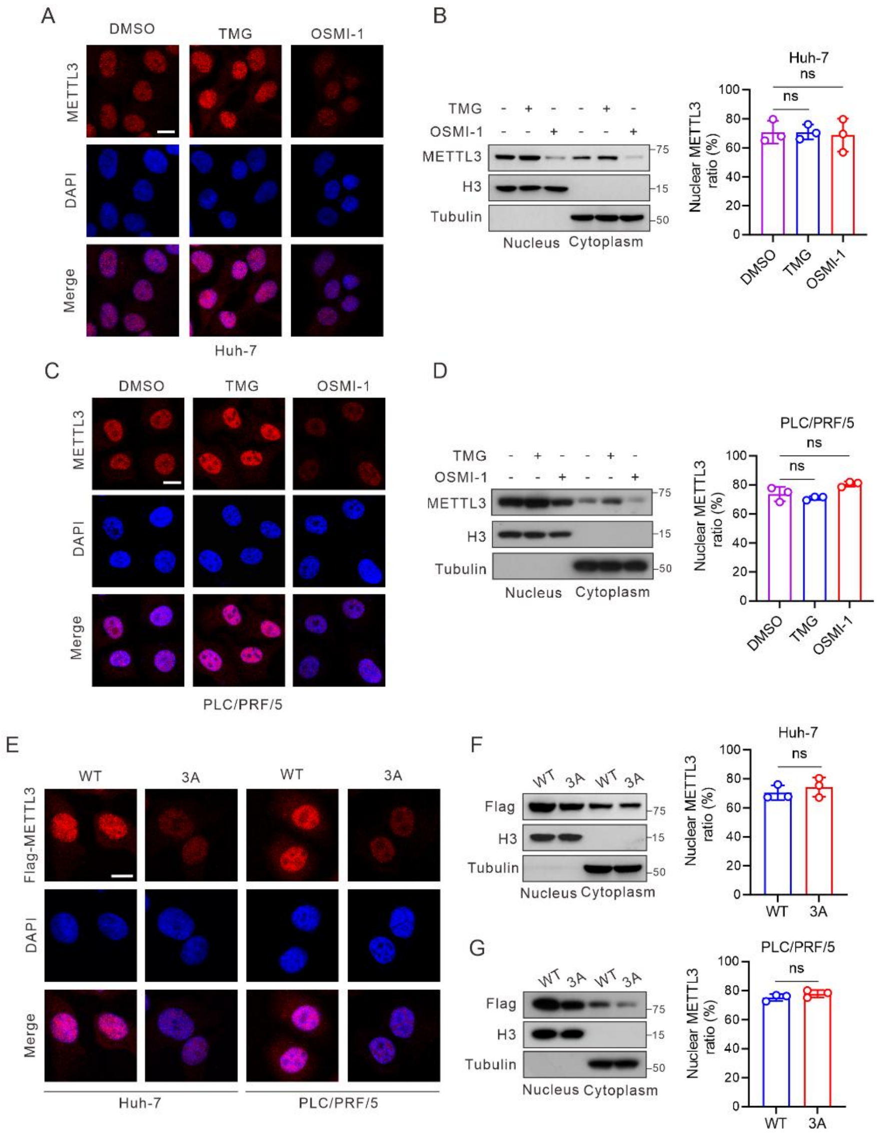

Fig. S5 O-GlcNAcylation of METTL3 does not influence its nucleocytoplasmic localization.

A–D Hepatoma cells were treated with DMSO, 25 μM Thiamet G, or 25 μM OSMI-1 for 12 h. Immunofluorescence staining was conducted to examine the subcellular localization of METTL3 in Huh-7 cells (A) and PLC/PRF/5 cells (C). Scale bar: 10 μm. Immunoblot analysis of the nuclear and cytoplasmic METTL3 in Huh-7 cells (B) and PLC/PRF/5 cells (D). Data were obtained from three independent experiments, analyzed by a one-way ANOVA test. **E–G** Hepatoma cells were transfected with Flag-METTL3-WT or 3A mutant for 48 h, followed by immunofluorescence (E) and immunoblotting (F, G) to evaluate METTL3 subcellular localization. Scale bar: 10 μm.

For F and G, Data were analyzed using an unpaired t-test.

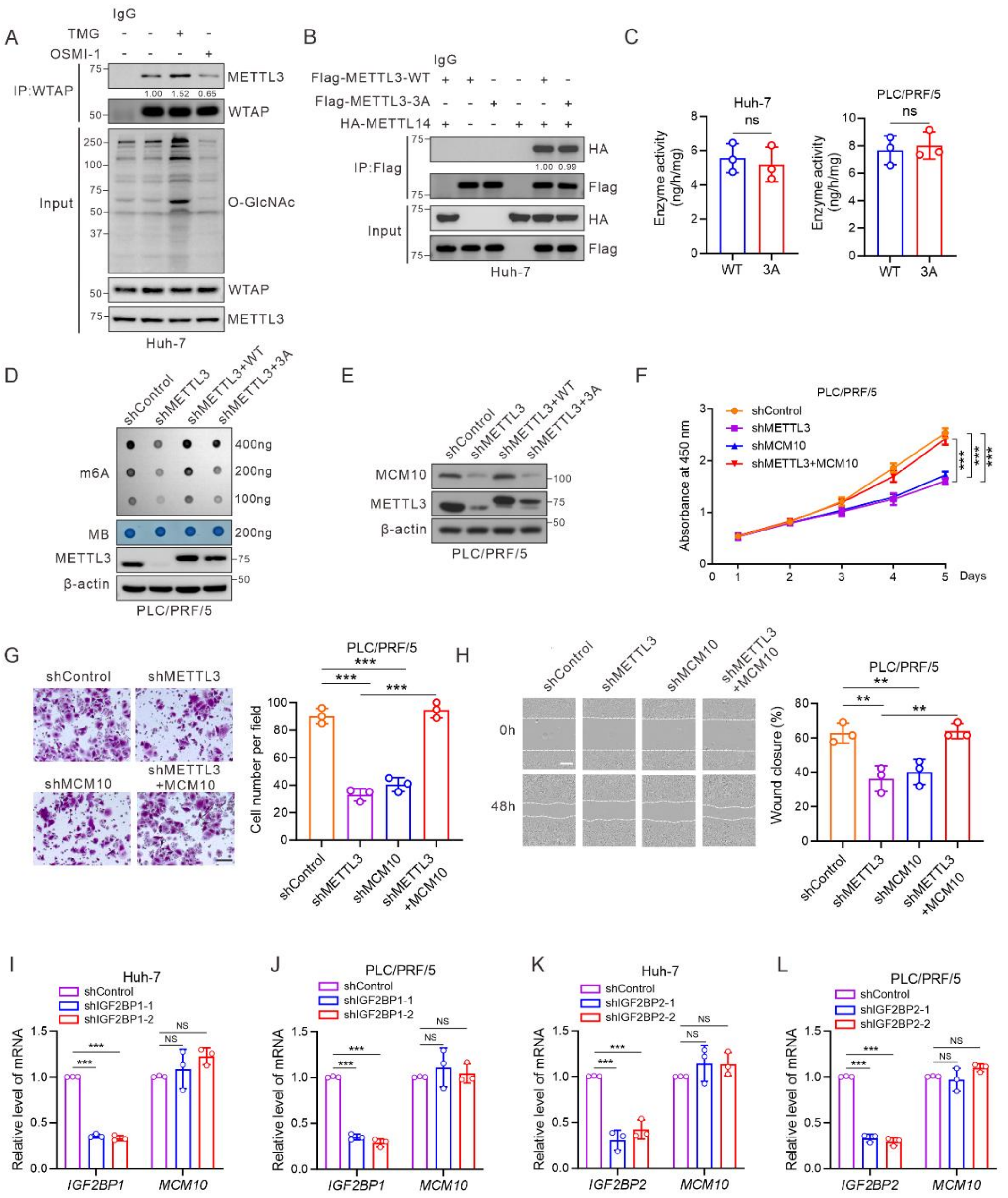

Fig. S6 METTL3 O-GlcNAcylation targets MCM10 to accelerate HCC progression.

A Huh-7 cells were treated with 25μM TMG or 20μM OSMI-1 for 12 h, then cells lysates were subjected to Co-IP assay to observe the interaction between METTL3 and WTAP. **B** Co-IP assay was conducted to investigate the association between METTL3 and METTL14. **C** Huh-7 and PLC/PRF/5 cells were infected with Ad-METTL3-Flag (either WT or 3A mutant). At 48 h after infection, the enzyme activity of purified proteins from hepatoma cells was measured. **D** Dot-blot assay was performed to access m6A levels in PLC/PRF/5 cells. **E** Immunoblotting to assess the impact of METTL3

O-GlcNAcylation on MCM10 protein levels in PLC/PRF/5 cells. **F–H** PLC/PRF/5 cells were infected with shMETTL3 or shMCM10 lentiviruses. Subsequently, PLC/PRF/5-shMETTL3 cells were infected with either Ad-GFP control or Ad-MCM10. Cells were then subjected to CCK-8 (F), Transwell (G, bar = 100 μm), and wound-healing (H, bar = 200 μm) assays. **I–L** RT-qPCR was conducted to observe alterations in *MCM10* mRNA levels following shIGF2BP1 or shIGF2BP2 treatment in Huh-7 and PLC/PRF/5 cells. For F–L, data were obtained from three independent experiments and are showed as mean ± SD. Data analysis was performed using a one-way ANOVA followed by the Tukey test, with significance indicated as $^{**}P < 0.01$ and $^{***}P < 0.001$.

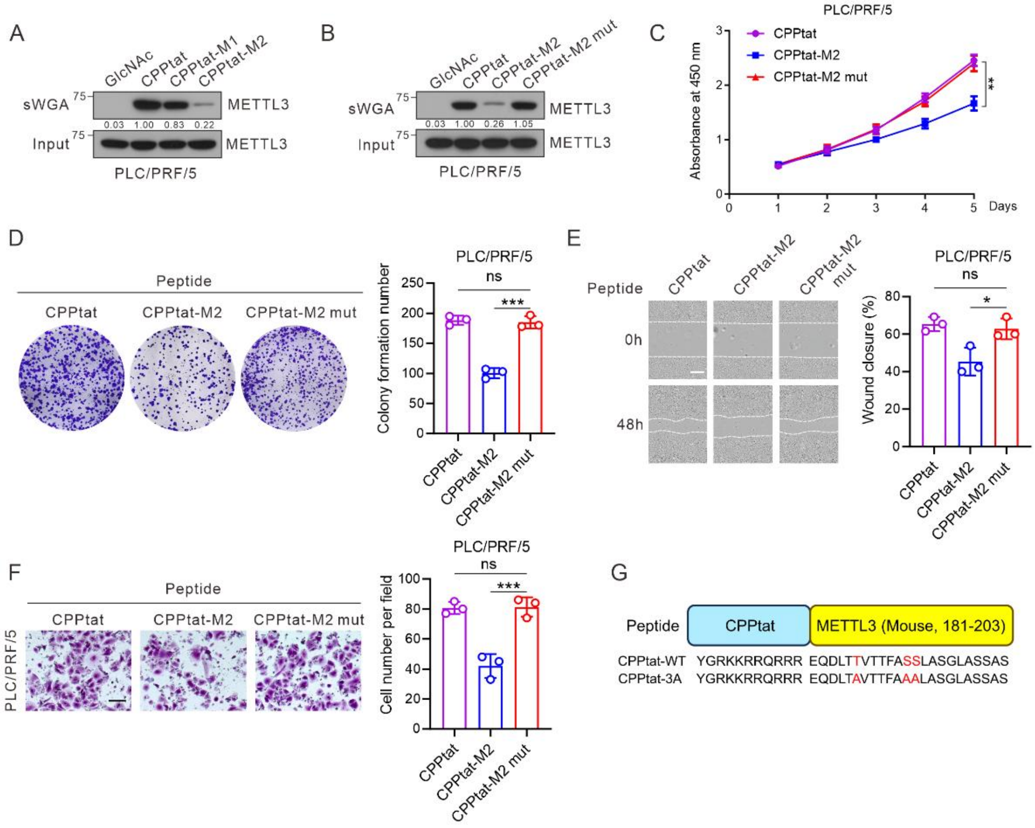

Fig. S7 Inhibition of METTL3 O-GlcNAcylation impedes HCC progression.

A, B sWGA pull-down assays to observe METTL3 O-GlcNAcylation levels following treatment with cell-penetrating peptides in PLC/PRF/5 hepatoma cells. **C–F** PLC/PRF/5 cells were treated with CPPtat, CPPtat-M2, or CPPtat-M2 mut (10 μM) for 24 h, then subjected to CCK-8 (C), colony formation (D), wound-healing (E, bar = 200 μm), and Transwell (F, bar = 100 μm) assays. Data are presented from three independent experiments. One-way followed by the Tukey test, *$P < 0.05$, **$P < 0.01$, ***$P < 0.001$. **G** Schematic of CPPtat-WT and CPPtat-3A. The different residues are highlighted in red.

Supplementary Table S1. Predicted MCM10 mRNA-binding proteins

AARS	ABCF1	ACI	AGGF1	AGO2	AKAP1
AKAP8L	ALKBH1	ALKBH5	ALYREF	APOBEC3C	ATXN2
AATF	AQR	AUH	BCCIP	BCLAF1	BUD13
CAPRIN1	CASP2	CDC40	CELF2	CEP85	CHTOP
CIRBP	CNBP	CPEB4	CPSF1	CPSF2	CPSF3
CPSF4	CPSF6	CPSF7	CSTF2	CSTF2T	DDX21
DDX24	DDX3	DDX3X	DDX42	DDX51	DDX52
DDX55	DDX59	DDX6	DGCR8	DHX30	DICER1
DIS3L2	DKC1	DROSHA	EIF3A	EIF3B	EIF3D
EIF3G	EIF3H	EIF4A3	EIF4G2	ELAVL1	ELAVL3
ENO1	EWSR1	EXOSC5	FASTKD2	FBL	FIP1L1
FKBP4	FMR1	FTO	FUBP3	FUS	FXR1
FXR2	G3BP1	GEMIN5	GNL3	GPKOW	GRSF1
GRWD1	GTF2F1	HNRNPA1	HNRNPA2B1	HNRNPC	HNRNPD
HNRNPF	HNRNPH	HNRNPH1	HNRNPK	HNRNPL	HNRNPM
HNRNPU	HNRNPUL1	IGF2BP1	IGF2BP2	IGF2BP3	ILF3
IMP3	KHDRBS1	KHSRP	LARP4	LARP7	LIN28A
LIN28B	LSM11	MATR3	MBNL1	MBNL2	METAP2
METTL14	METTL3	MOV10	MTPAP	NCBP2	NCBP3
NIP7	NIPBL	NKRF	NOL12	NOLC1	NONO
NOP56	NOP58	NPM1	NSUN2	NUDT21	NXF1
PABPC1	PABPC4	PABPN1	PCBP1	PCBP2	PHF6
POLR2G	PPIG	PPIL4	PRKRA	PRPF4	PRPF8
PTBP1	PUM1	PUM2	PUS1	QKI	RBFOX1
RBFOX2	RBM10	RBM15	RBM15B	RBM22	RBM27
RBM47	RBM5	RBPMS	RPS11	RPS3	RPS5
RTCB	SAFB	SAFB2	SBDS	SDAD1	SDOS
SERBP1	SF3A3	SF3B1	SF3B4	SFPQ	SLBP
SLTM	SMN	SMNDC1	SND1	SRRM4	SRSF1
SRSF10	SRSF3	SRSF7	SRSF9	SSB	STAU1
STAU2	SUB1	SUGP2	SUPV3L1	TAF15	TARBP2
TARDBP	TBRG4	TDP43	TIA1	TIAL1	TNRC6A
TNRC6B	TNRC6C	TRA2A	TROVE2	U2AF1	U2AF2
UCHL5	UNR	UPF1	UTP18	UTP3	WDR3
WDR33	WDR43	WRN	WTAP	XPO5	XRCC6
YBX3	YTHDC1	YTHDC2	YTHDF1	YTHDF2	YTHDF3
YWHAG	ZC3H11A	ZC3H7B	ZC3H8	ZNF622	ZNF800
ZRANB2					

Supplementary Table S2. Primers and oligos used in the studies

Name	Sequence (5’>3’)
**Oligos for shRNA**	
shOGT	Forward:<br>TGCATGTTATTTGAAAGCAATTCAAGAGATTGCTTTC<br>AAATAACATGCTTTTTC<br>Reverse:<br>TCGAGAAAAAAGCATGTTATTTGAAAGCAATCTCTT<br>GAATTGCTTTCAAATAACATGCA
shMETTL3	Forward:<br>TGCTAAACCTGAAGAGTGATATTTCAAGAGAATATC<br>ACTCTTCAGGTTTAGCTTTTTC<br>Reverse:<br>TCGAGAAAAAAGCTAAACCTGAAGAGTGATATTCTC<br>TTGAAATATCACTCTTCAGGTTTAGCA [4]
shMCM10	Forward:<br>TGCAAATGAAGGCCTTACAAGATTCAAGAGATCTTG<br>TAAGGCCTTCATTTGCTTTTTC<br>Reverse:<br>TCGAGAAAAAAGCAAATGAAGGCCTTACAAGATCT<br>CTTGAATCTTGTAAGGCCTTCATTTGCA [5]
shIGF2BP1	Forward1:<br>TGCTGCTCCTCCGCTTGTAAGATTCAAGAGATCTTA<br>CAAGCGGAGGAGCAGCTTTTTC<br>Reverse1:<br>TCGAGAAAAAAGCTGCTCCTCCGCTTGTAAGATCTC<br>TTGAATCTTACAAGCGGAGGAGCAGCA<br>Forward2:<br>TGGAAATAATGAAGAAAGTTCGTTCAAGAGACGAA<br>CTTTCTTCATTATTTCCTTTTTC<br>Reverse2:<br>TCGAGAAAAAAGGAAATAATGAAGAAAGTTCGTCT<br>CTTGAACGAACTTTCTTCATTATTTCCA
shIGF2BP2	Forward1:<br>TGCCGTTGTCAACGTCACATATTTCAAGAGAATATGT<br>GACGTTGACAACGGCTTTTTC<br>Reverse1:<br>TCGAGAAAAAAGCCGTTGTCAACGTCACATATTCTC<br>TTGAAATATGTGACGTTGACAACGGCA<br>Forward2:<br>TGCATGATTCTTGAAATCATGCTTCAAGAGAGCATG<br>ATTTCAAGAATCATGCTTTTTC<br>Reverse2:<br>TCGAGAAAAAAGCATGATTCTTGAAATCATGCTCTC<br>TTGAAGCATGATTTCAAGAATCATGCA
shIGF2BP3	Forward1:<br>TGGAAGTGCTGAATGGTGTTGGTTCAAGAGACCAA<br>CACCATTCAGCACTTCCTTTTTC<br>Reverse1:<br>TCGAGAAAAAAGGAAGTGCTGAATGGTGTTGGTCT<br>CTTGAACCAACACCATTCAGCACTTCCA<br>Forward2:<br>TGCCAAACCAAAGACAGATTGCTTCAAGAGAGCAA<br>TCTGTCTTTGGTTTGGCTTTTTC<br>Reverse2:<br>TCGAGAAAAAAGCCAAACCAAAGACAGATTGCTCT

		CTTGAAGCAATCTGTCTTTGGTTTGGCA
**Primers for recombinant DNA**		
pSEB-3Flag-METTL3	Forward:	TCCAAGCTTACCATGGGCTCGGACACGTGGAGCT
	Reverse:	CGCGTCGACTAAATTCTTAGGTTTAGAGATGAT
pAdTrack-TO4-3Flag-METTL3	Forward:	ATAGCGGCCGCACCATGGGCTCGGACACGTGGAGCT
	Reverse:	CCCAAGCTTTTAAATCTTATCGTCGTCATCCT
pET28a(+)-6xHis-METTL3	Forward:	TCCAAGCTTGCTCGGACACGTGGAGCTC
	Reverse:	ATTTGCGGCCGCCTATAAATTCTTAGGTTTAGAGATG
pBudCE4.1-3HA-FBXW7	Forward:	TGGGGTACCATGGGCAATCAGGAACTGCTCTCTGTG
	Reverse:	TGAAGATCTGACTTCATGTCCACATCAAAGTCC
pBuCE4.1-3HA-OGT	Forward:	ATAGCGGCCGCACCATGGCGTCTTCCGTGGGC
	Reverse:	CGCGGATCCTGCTGACTCAGTGACTTCAACAGG
pGEX-6P-1-GST-OGT (aa313- 1031)	Forward:	ACGCGTCGACACCATGGCGTCTTCCGTGGGC
	Reverse:	CGCGGATCCTGCTGACTCAGTGACTTCAACAGG
pBudCE4.1-3HA-METTL14	Forward:	CGGGGTACCATGGATAGCCGCTTGCAG
	Reverse:	CCGCTCGAGTCTCGAGGTGGAAAGCCACC
pBudCE4.1-3HA-WTAP	Forward:	CGGGGTACCATGACCAACGAAGAACCTCTTC
	Reverse:	CCGCTCGAGTCCAAAACTGAACCCTGTACATTTA
pSEB-3Flag-METTL14	Forward:	CCCAAGCTTACCATGGATAGCCGCTTGCAG
	Reverse:	TGCGGATCCGTATCGGCTCCTCCCACC
pSEB-3Flag-WTAP	Forward:	ATAGCGGCCGCACCATGACCAACGAAGAACCTCTTC
	Reverse:	CGCGGATCCCAAAACTGAACCCTGTACATTTA
pAdTrack-TO4-MCM10	Forward:	CGGGGTACCATGGATGAGGAGGAAGACA
	Reverse:	CCC AAGCTTTTATTTAAGGCTGTTCAGAAA
**Primers for mutagenesis**		
pAdTrack-TO4-3Flag-METTL3-T186A	Forward:	CAGGACTCGACTGCAGTAGCTGCCTTTGC
	Reverse:	AGGCAGCTACTGCAGTCGAGTCCTGTTCT
pAdTrack-TO4-3Flag-METTL3-S192A	Forward:	TAGCTGCCTTTGCCGCTTCGTTAGTCTCTGG
	Reverse:	GAGACTAACGAAGCGGCAAAGGCAGCTACTG
pAdTrack-TO4-3Flag-	Forward:	

METTL3-S193A	CTGCCTTTGCCAGTGCGTTAGTCTCTGGTC<br>Reverse:<br>CCAGAGACTAACGCACTGGCAAAGGCAGCT
pAdTrack-TO4-3Flag-METTL3-T186A/S192A/S193A	Forward:<br>CAGGACTCGACTGCAGTAGCTGCCTTTGCCGCTGCGTTAGTCTCTGGTC<br>Reverse:<br>CCAGAGACTAACGCAGCGGCAAAGGCAGCTACTGCAGTCG AGTCCTGTT
pSEB-3Flag-ΔC (METTL3 1aa-259aa)	Forward:<br>TCCAAGCTTACCATGGGCTCGGACACGTGGAGCT<br>Reverse:<br>CGCGTCGACGGATTGTTCCTTGGCTGTT
pSEB-3Flag-ΔN (METTL3 260aa-580aa)	Forward:<br>TCCAAGCTTACCATGGGCATTGTTGAAAAATTTCGCTC<br>Reverse:<br>CGCGTCGACTAAATTCTTAGGTTTAGAGATGAT
**Primers for Real-time quantitative PCR**	
METTL3-Human	Forward:<br>CCAAAAGGTCAAGGAAACAAA<br>Reverse:<br>GGAGAAGCCAATGGAGGG
UHRF1-Human	Forward:<br>CGGGAACTCTACGCCAACG<br>Reverse:<br>TGTCGCACTCATCGCACAT
CDC25A-Human	Forward:<br>CTCCGAGTCAACAGATTCAGG<br>Reverse:<br>GGCAGCCACGAGATACAGG
HSPA8-Human	Forward:<br>ATCAGGGAAACCGAACCACT<br>Reverse:<br>CATCAAATCTGCGTCCAATCA
AMD1-Human	Forward:<br>GTGAGCTTGACCCAGCAGTT<br>Reverse:<br>AGTTCCATCCGATTTCATTCC
ATAD3B-Human	Forward:<br>TGGAGGGTGTTGTGCTTAGTC<br>Reverse:<br>GCAGGGCGAGTTTCTTGG
LRRC59-Human	Forward:<br>CGGCGGCTGGAAGTAGAAC<br>Reverse:<br>GCTTGGCTGCTTTGAGGG
CHPF2-Human	Forward:<br>GGCAGCAGTATCGCTCATTT<br>Reverse:<br>CCTGAGCGGTTGGTTTGG
SRRT-Human	Forward:<br>TCAGCCCGCCACAGAAG<br>Reverse:<br>TCATCCACCGAGTCATCCAG
NOL6-Human	Forward:<br>CCGCCCTTGGACATTTACG

	Reverse: GGAGGATCATAGCCCAGCAC
MCM10-Human	Forward: AGAAGCAGCGGATGTTGGA<br>Reverse: TTTGGTCTTGGTGGTGGTGA
MCM10-Mouse	Forward: AGCGAGCAGCATAACCTCC<br>Reverse: ATTCCATCCCGTTCCCAC
IGF2BP1-Human	Forward: GGGCAGCACATCAAACAGC<br>Reverse: ATAGATTCTTCCCTGAGCCTTG
IGF2BP2-Human	Forward: AAACATCCCTCCTCACCTGC<br>Reverse: TCAAACTGATGCCCGCTTA
IGF2BP3-Human	Forward: CCGTATCCAAGCAGAAACCA<br>Reverse: GAATAGACTTACAAGCCGCAGA
β-actin-Human	Forward: AGGCCAACCGCGAGAAGATGACC<br>Reverse: GAAGTCCAGGGCGACGTAGCAC
β-actin-Mouse	Forward: CGTTCAATACCCCAGCCATG<br>Reverse: GACCCCGTCACCAGAGTCC

Supplementary Table S3. Summary of peptides sequence

Peptides	Sequence
CPPtat	YGRKKRRQRRR
CPPtat-M1	YGRKKRRQRRRRAEQDSTTVAAFASSLVSGLNS
CPPtat-M2	YGRKKRRQRRREQDSTTVAAFASSLVSGLNSSAS
CPPtat-M2 mut	YGRKKRRQRRREQDSTAVAAFAAALVSGLNSSAS
CPPtat-WT	YGRKKRRQRRREQDLTTVTTFASSLASGLASSAS
CPPtat-3A	YGRKKRRQRRREQDLTAVTTFAAALASGLASSAS

307 **Supplementary Table S4. List of antibodies in the studies**

Antibodies	Source	Identifier
Rabbit anti-METTL3	Abcam	ab195352
Mouse anti-HA	Invitrogen	26183
Mouse anti-Flag	Sigma	F3165
Mouse anti-O-GlcNAc	Abcam	ab2739
Mouse anti-GAPDH	Beyotime	AG019
Mouse anti-β-actin	ZSGB-BIO	TA-09
Goat anti-rabbit, secondary	Bio-rad	1706515
Goat anti-mouse, secondary	Bio-rad	1706516
Rabbit anti-OGT	Abcam	ab96718
Mouse anti-OGT	Santa Cruz	sc-4546
Goat anti-Rabbit IgG, Alexa Fluor ® 488	Invitrogen	A-11034
Goat anti-Mouse IgG, Alexa Fluor ®594	Invitrogen	A-21125
Mouse anti-m6A	Abcam	ab208577
Mouse anti-Myc	Proteintech	60003-2-Ig
Mouse anti-PCNA	Proteintech	60097-1-Ig
Rabbit anti-IGF2BP3	Proteintech	81805-1-RR
Rabbit anti-β-Tubulin	Proteintech	80713-1-RR

Rabbit anti-Histone H3	Proteintech	17168-1-AP
Rabbit anti-MCM10	Proteintech	12251-1-AP
Mouse anti-IgG Control	Invitrogen	10400C
Rabbit anti-IgG Control	Proteintech	30000-0-AP
