# Supplementary material for: O-GlcNAcylation of METTL3 drives hepatocellular carcinoma progression by upregulating MCM10 expression in an m6A-IGF2BP3-dependent manner
Source: Cell Death Dis. 2025 Jul 12;16(1):518. doi: 10.1038/s41419-025-07844-1 (PMC12255776; doi:10.1038/s41419-025-07844-1)

**Figure 1A**


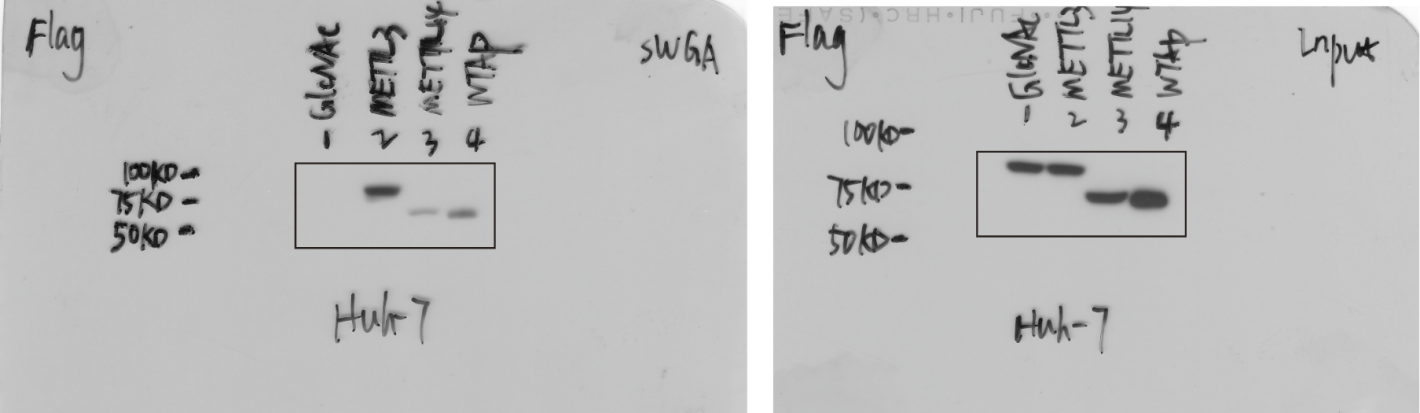


**Figure 1B**


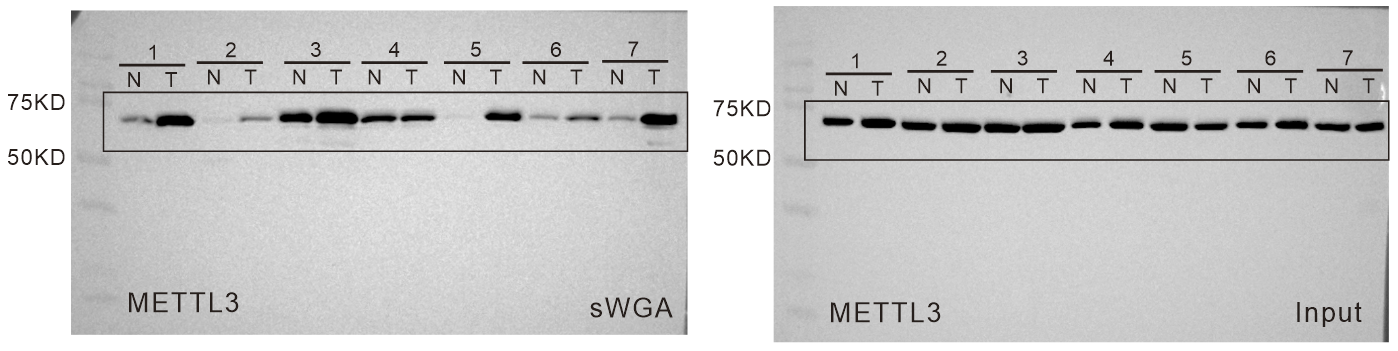


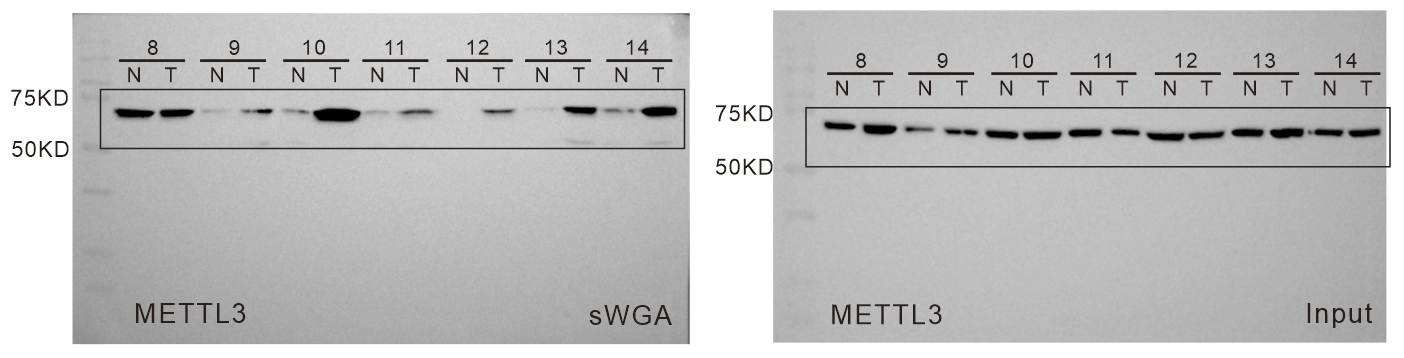


**Figure 1D**


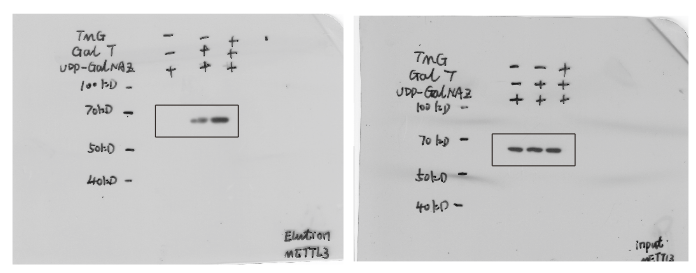


**Figure 1D**


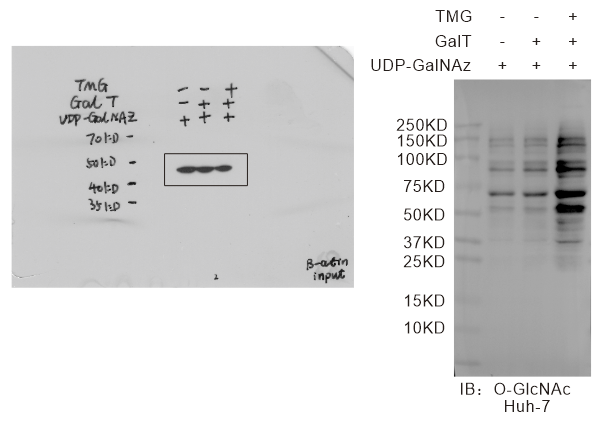


**Figure 1E**


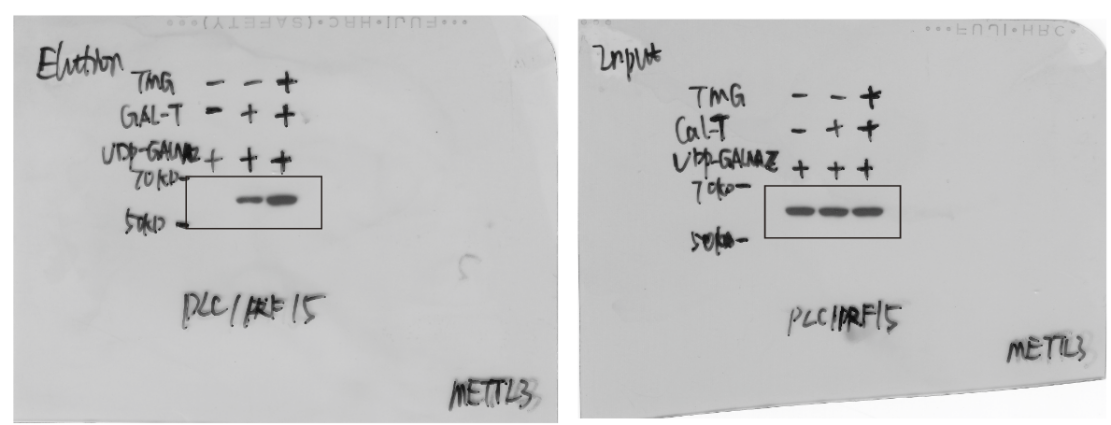


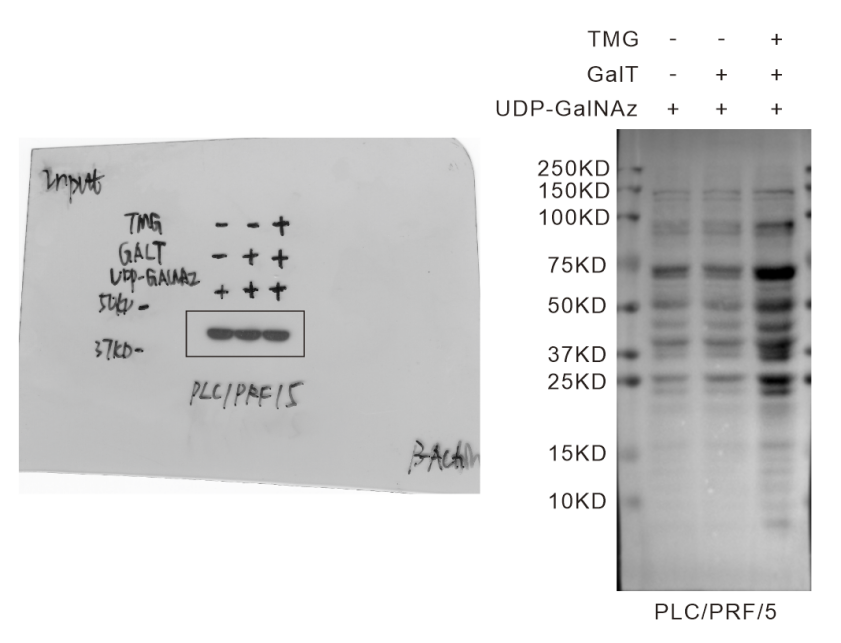


**Figure 1F**


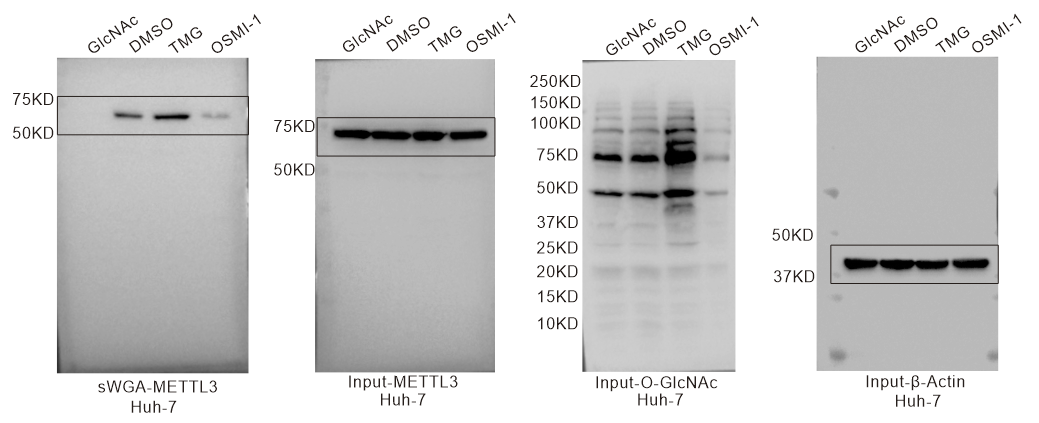


**Figure 1G**


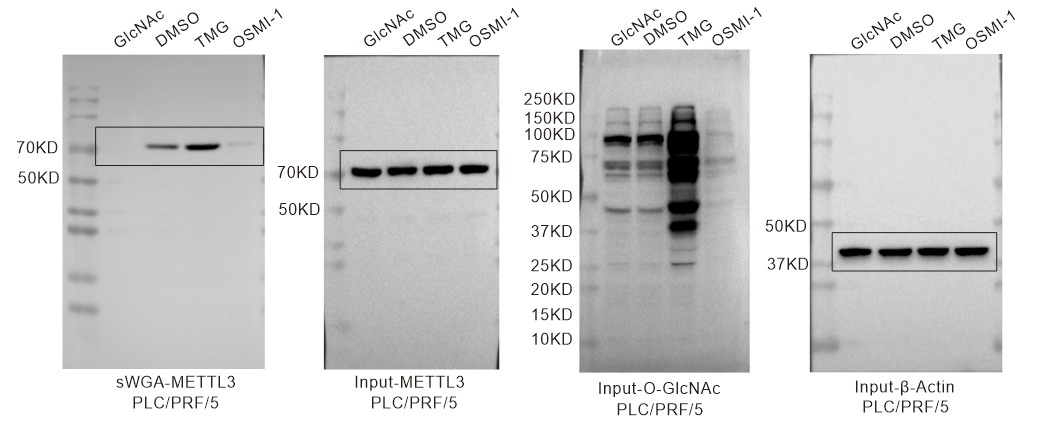


**Figure 1H**


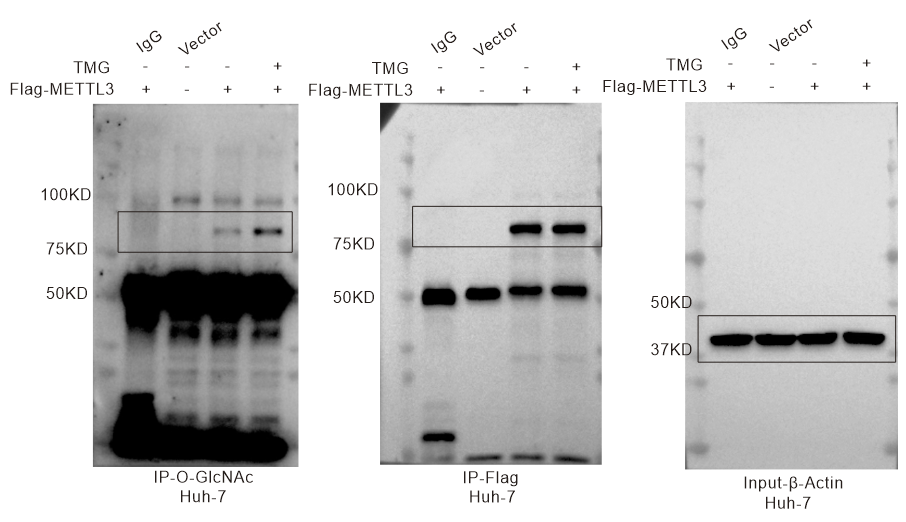


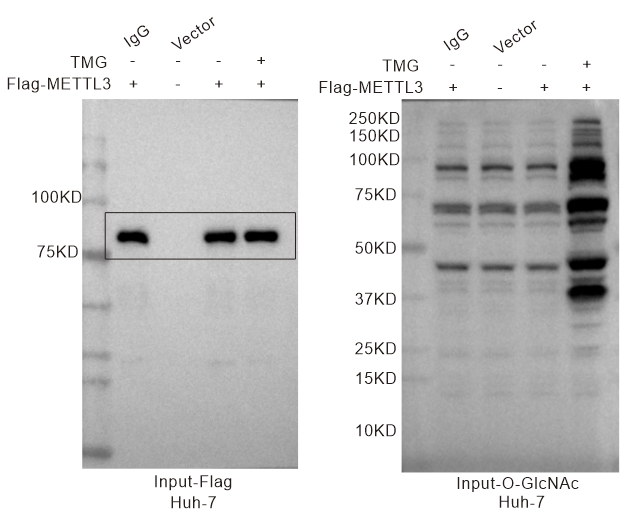


**Figure 1I**


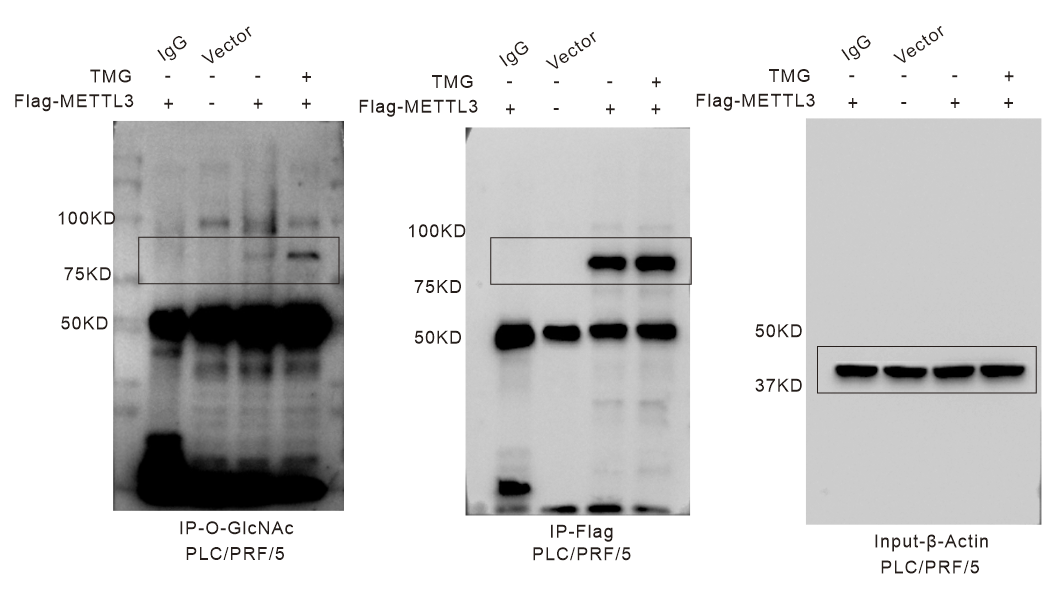


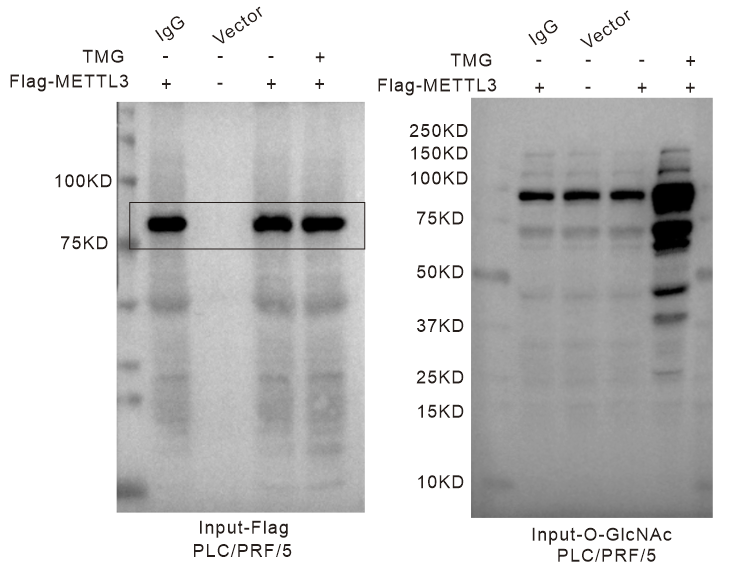


**Figure 1J**


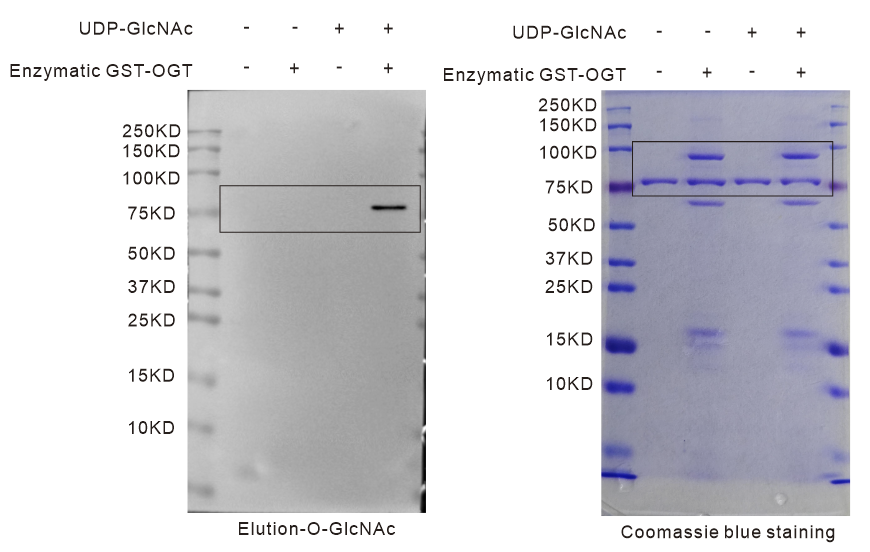


**Figure 2A**


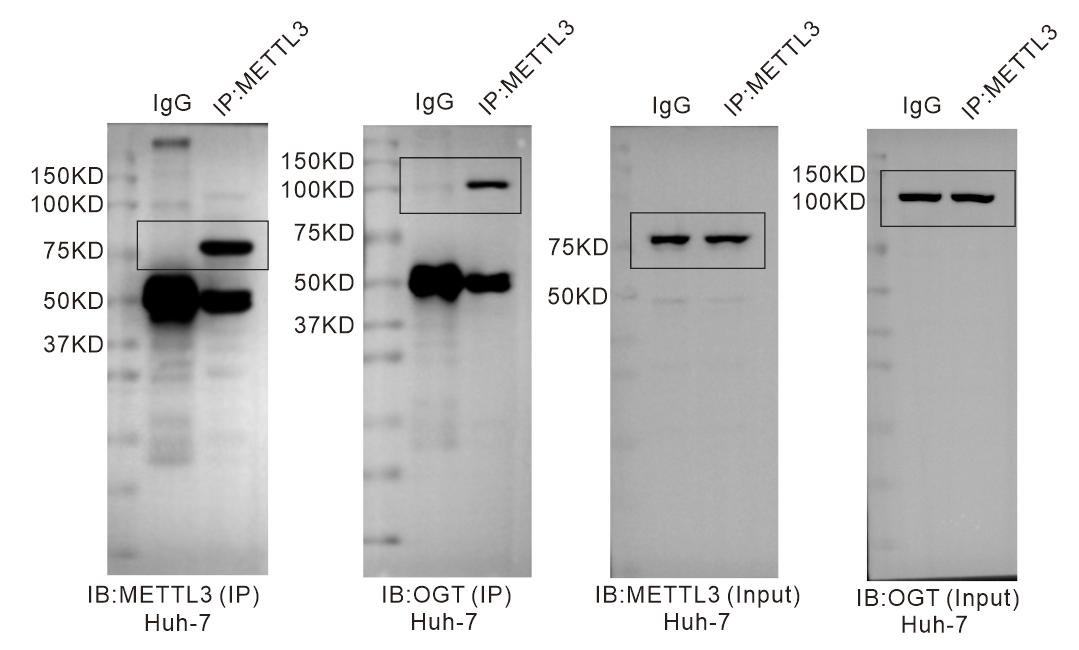


**Figure 2B**


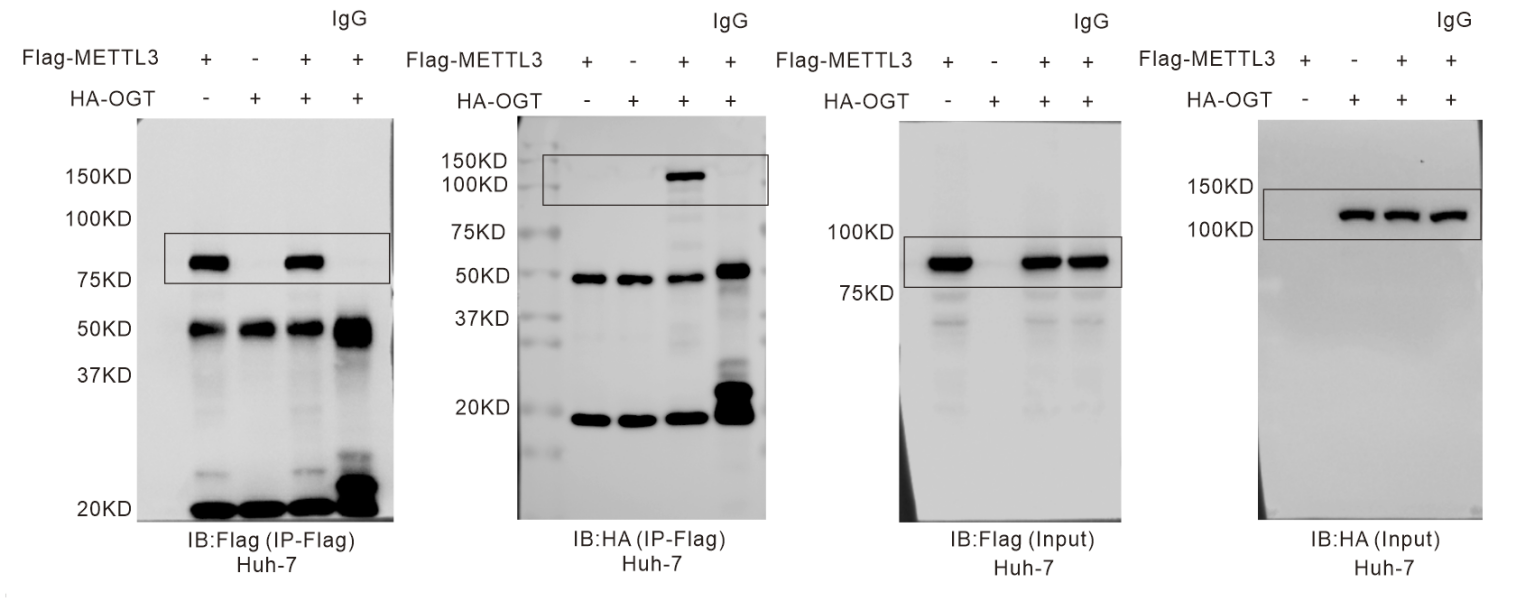


**Figure 2C**


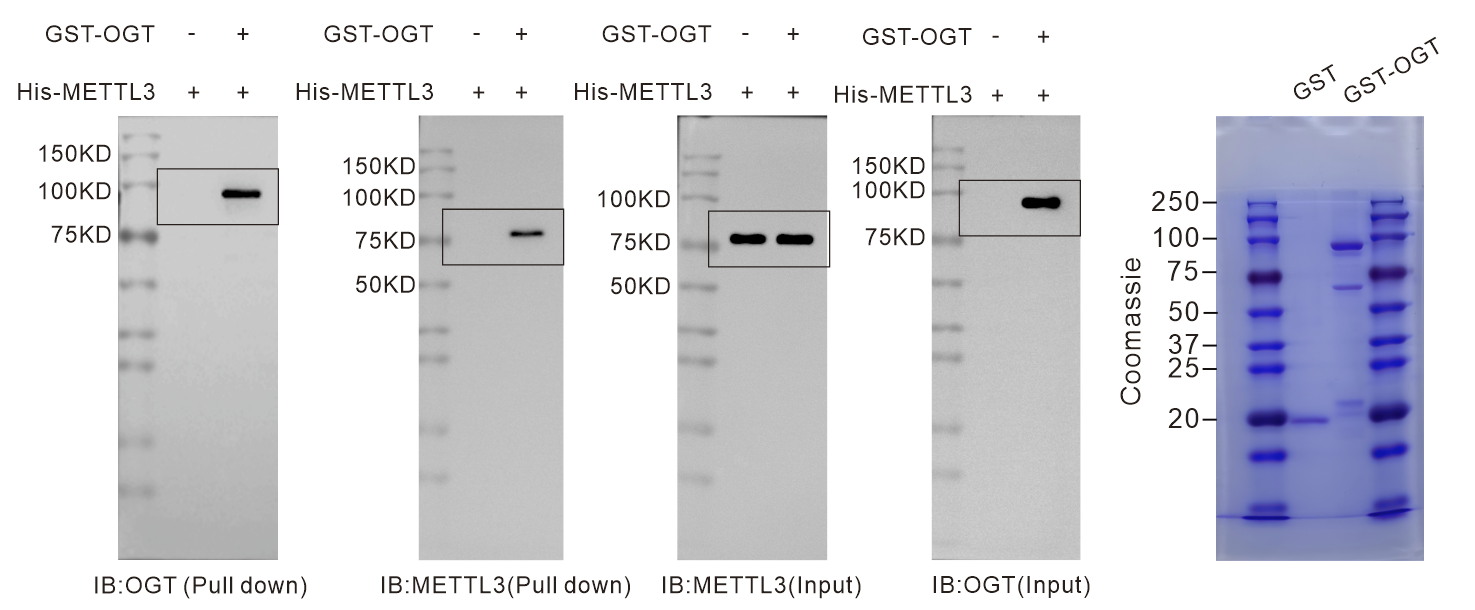


**Figure 2F**

**
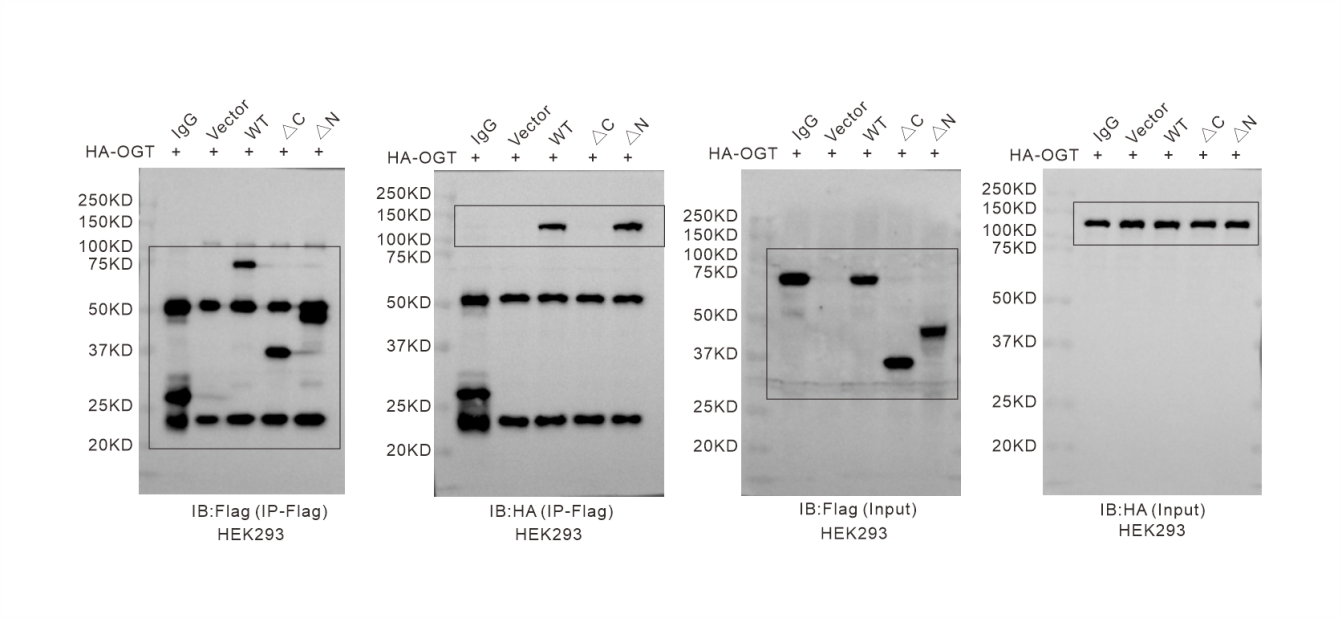
**

**Figure 2I**


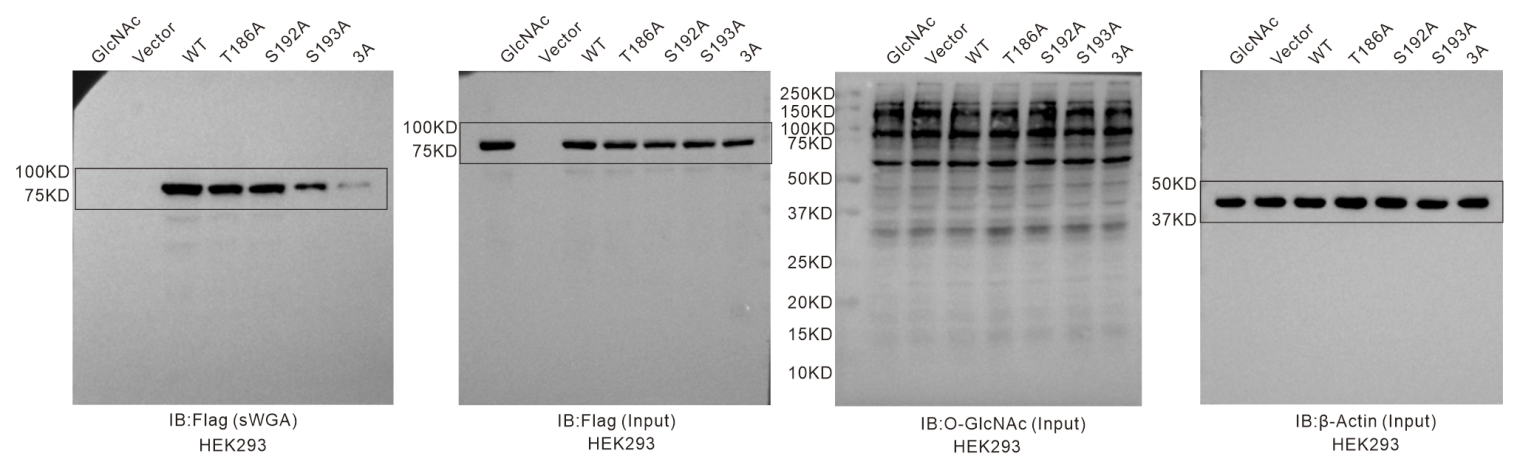


**Figure 2J**


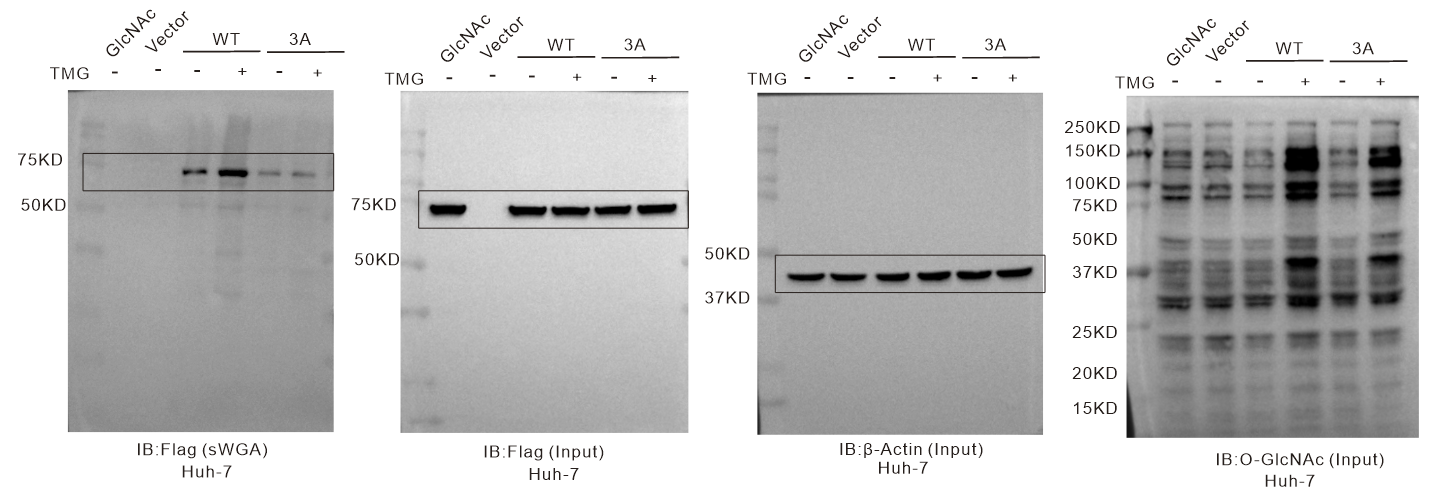


**Figure 2K**


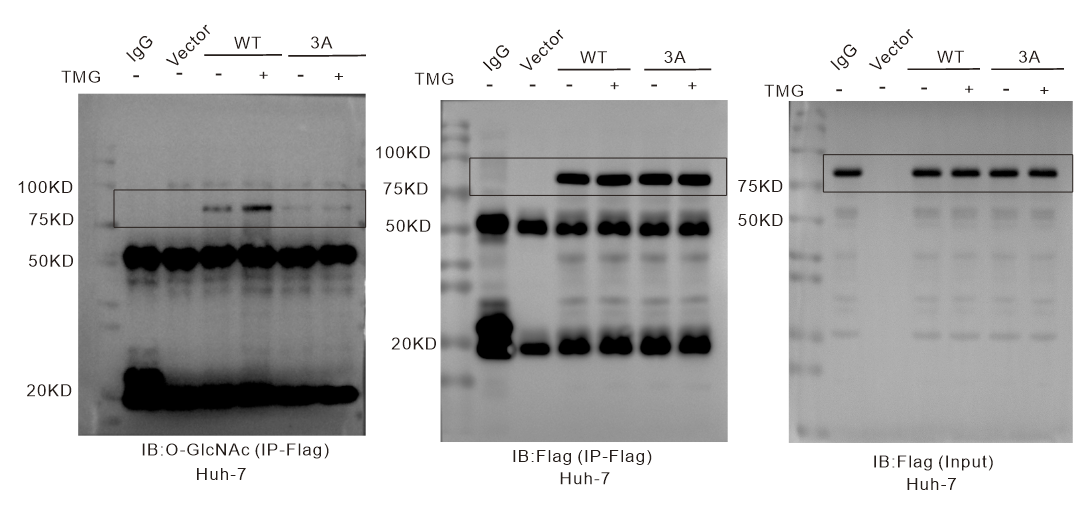


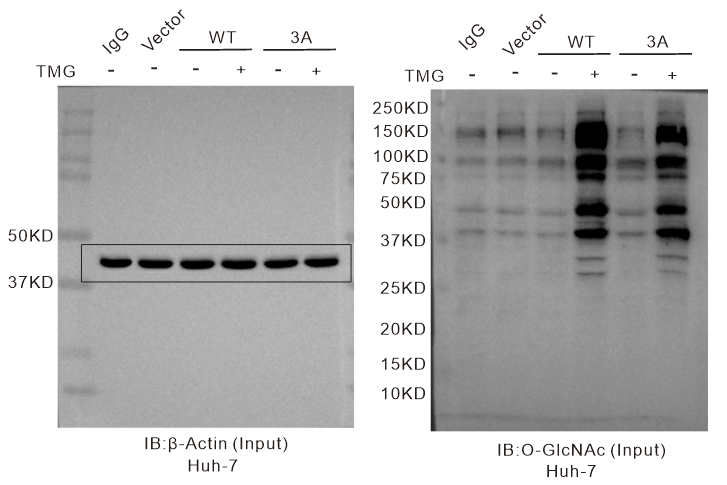


**Figure 3A**


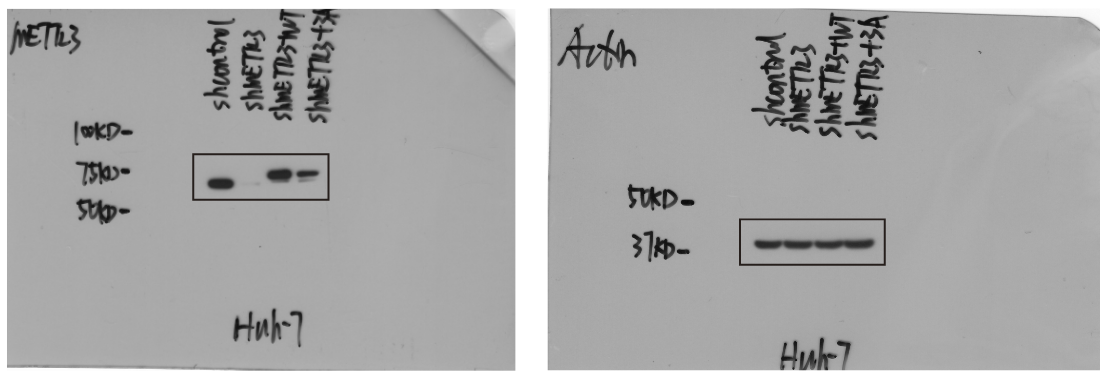


**Figure 3A**


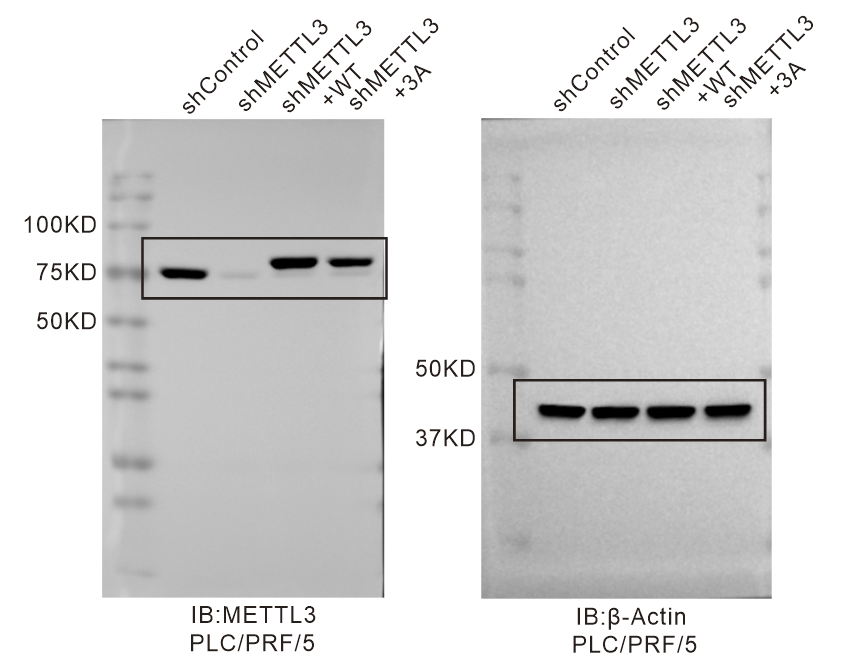


**Figure 4A**


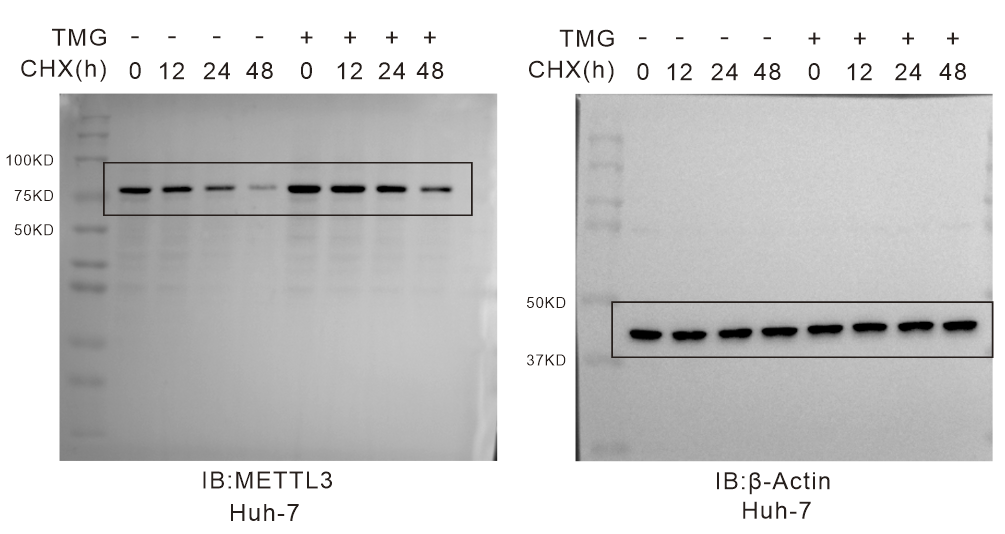


**Figure 4B**


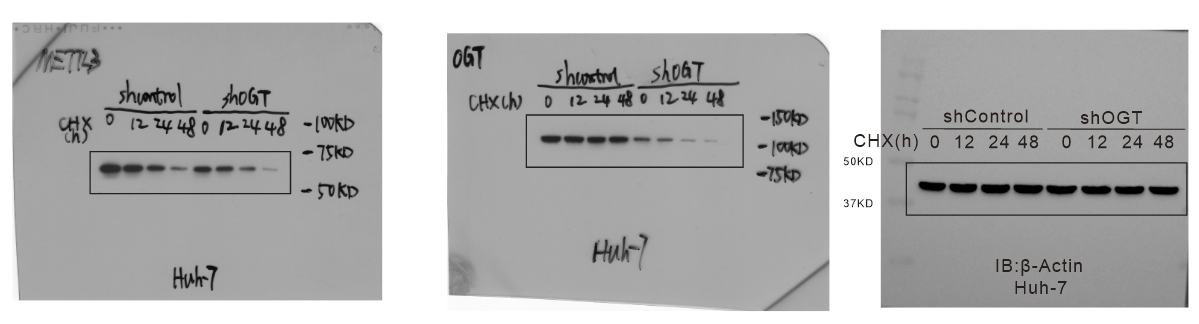


**Figure 4C**


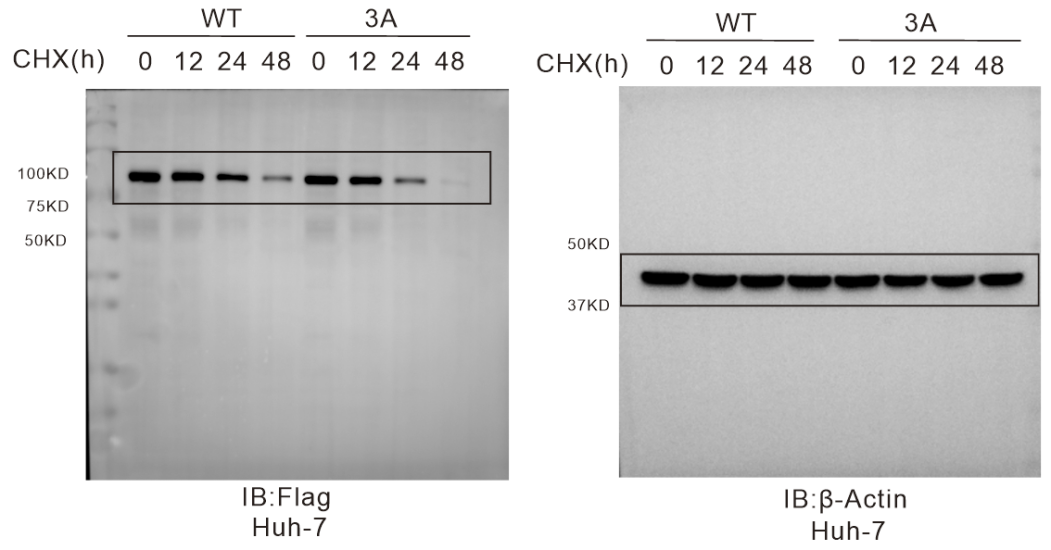


**Figure 4D**


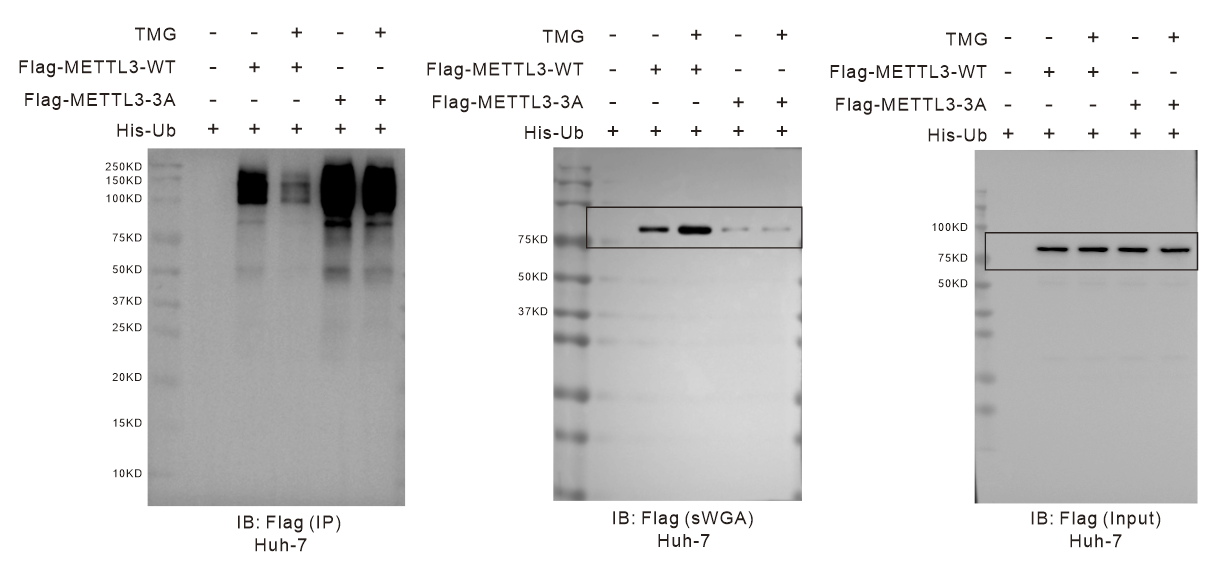


**Figure 4E**


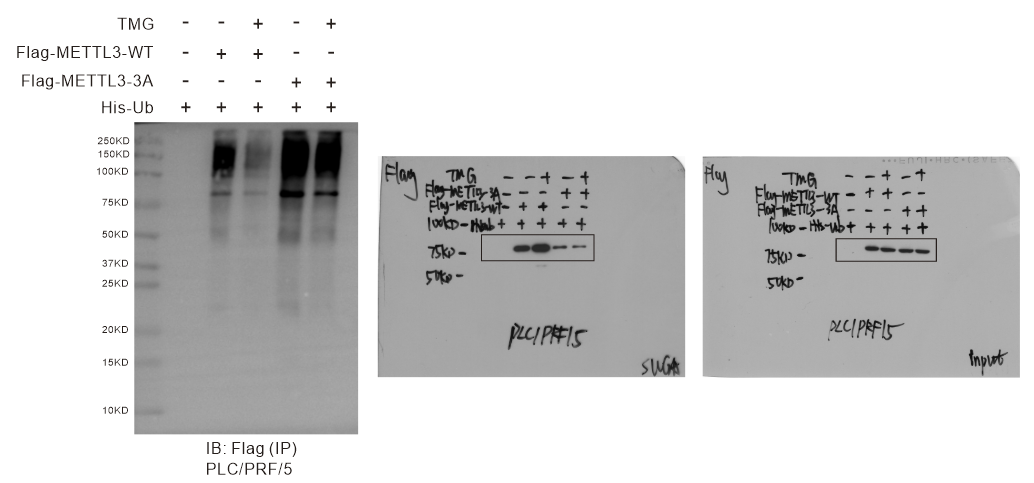


**Figure 4F**


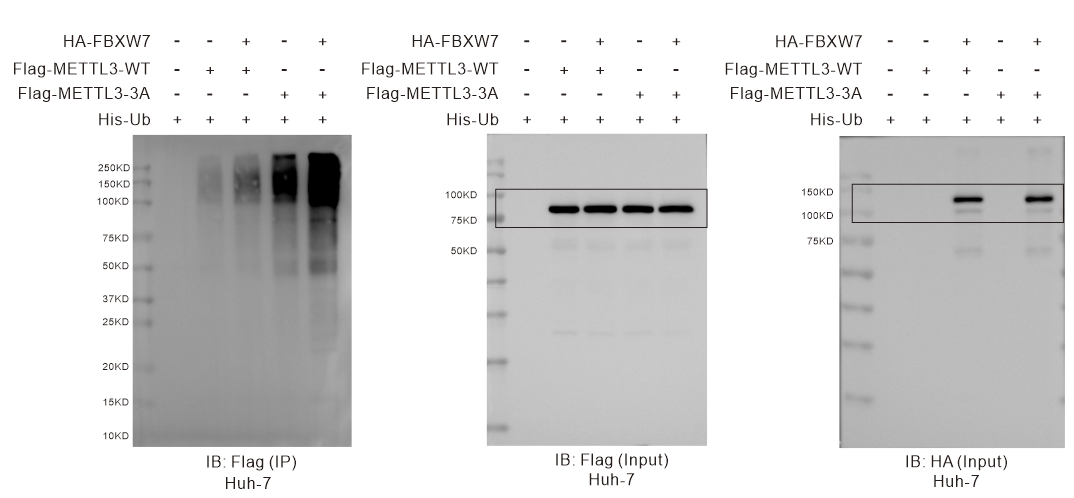


**Figure 4G**


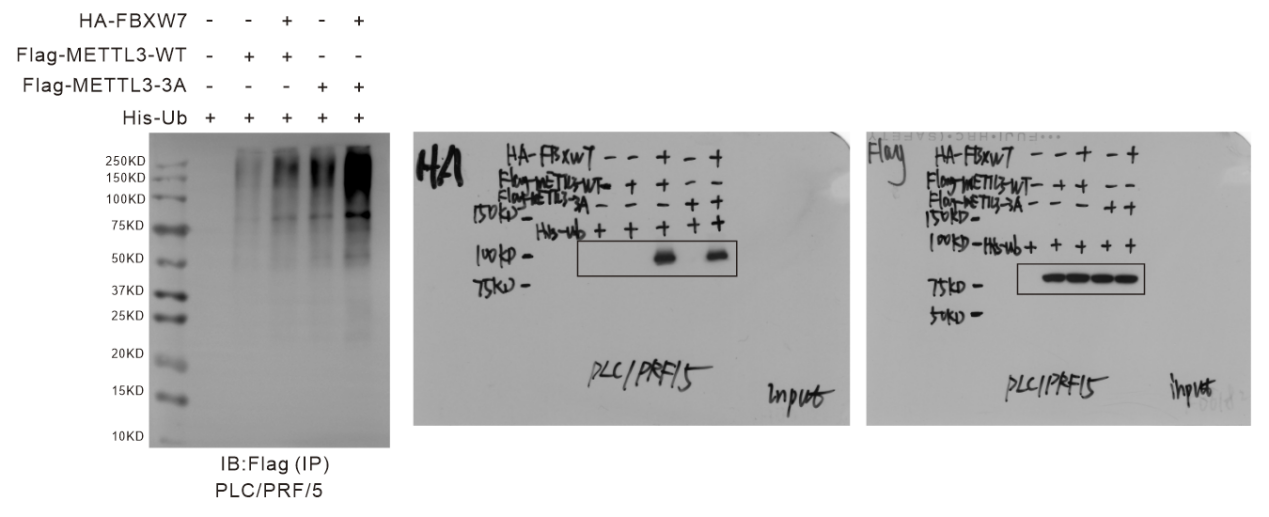


**Figure 4H**


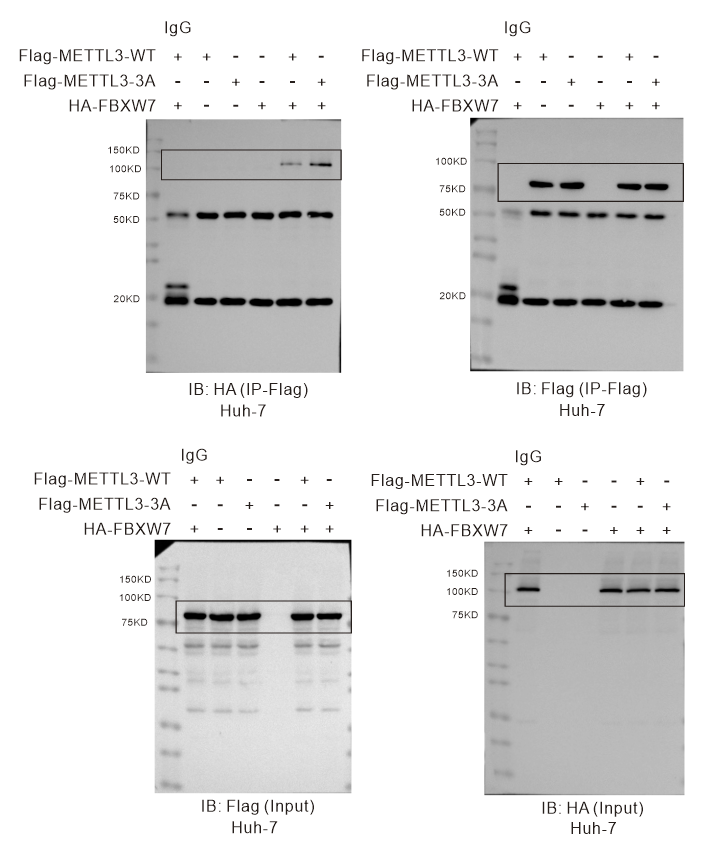


**Figure 4I**


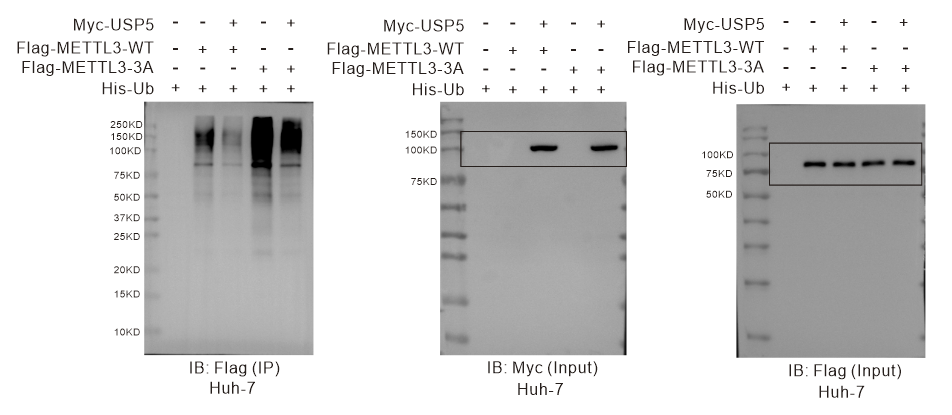


**Figure 5B**


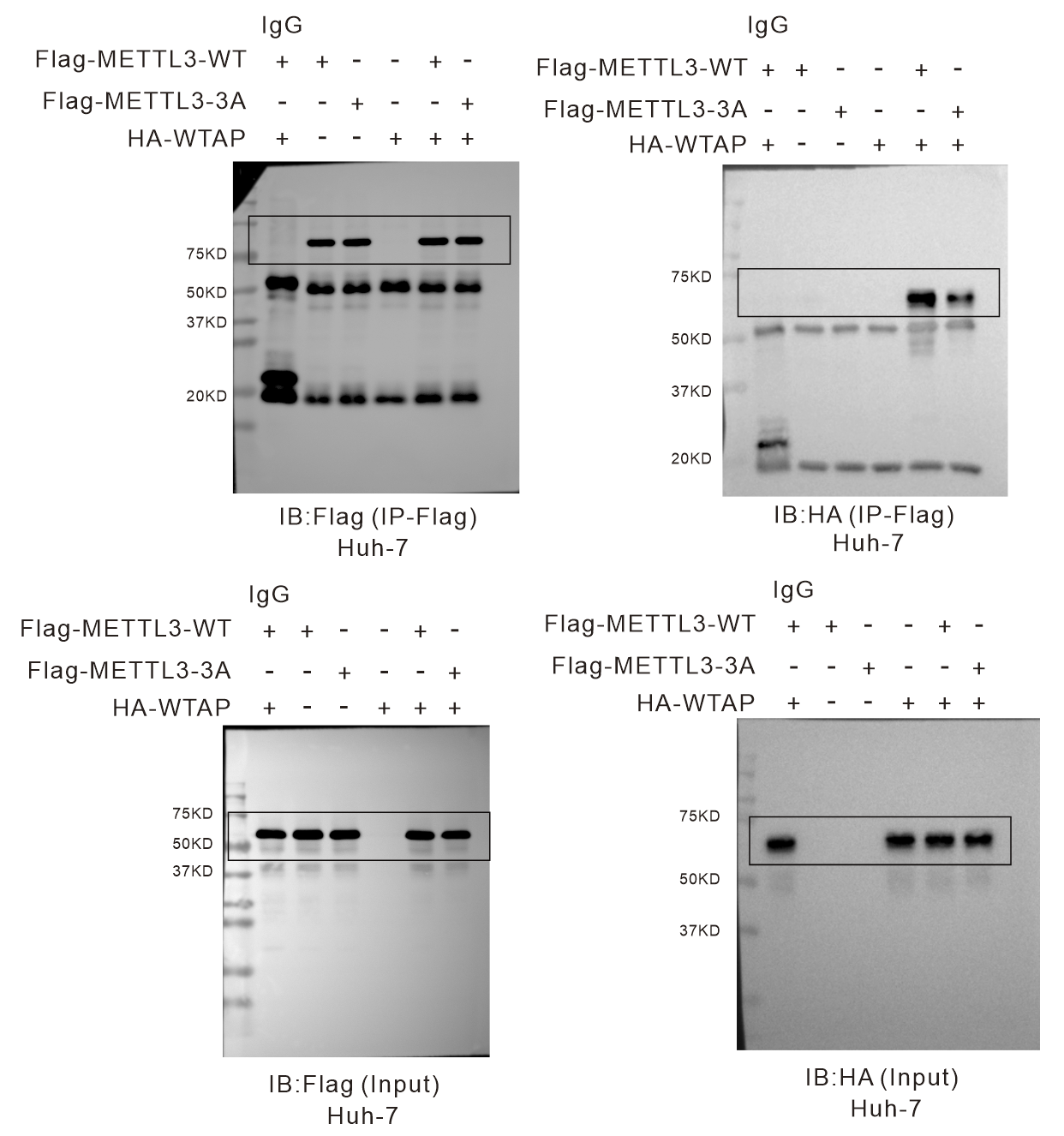


**Figure 5C**


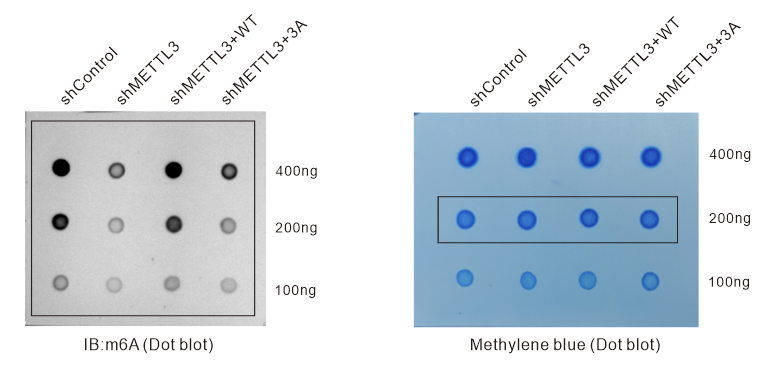


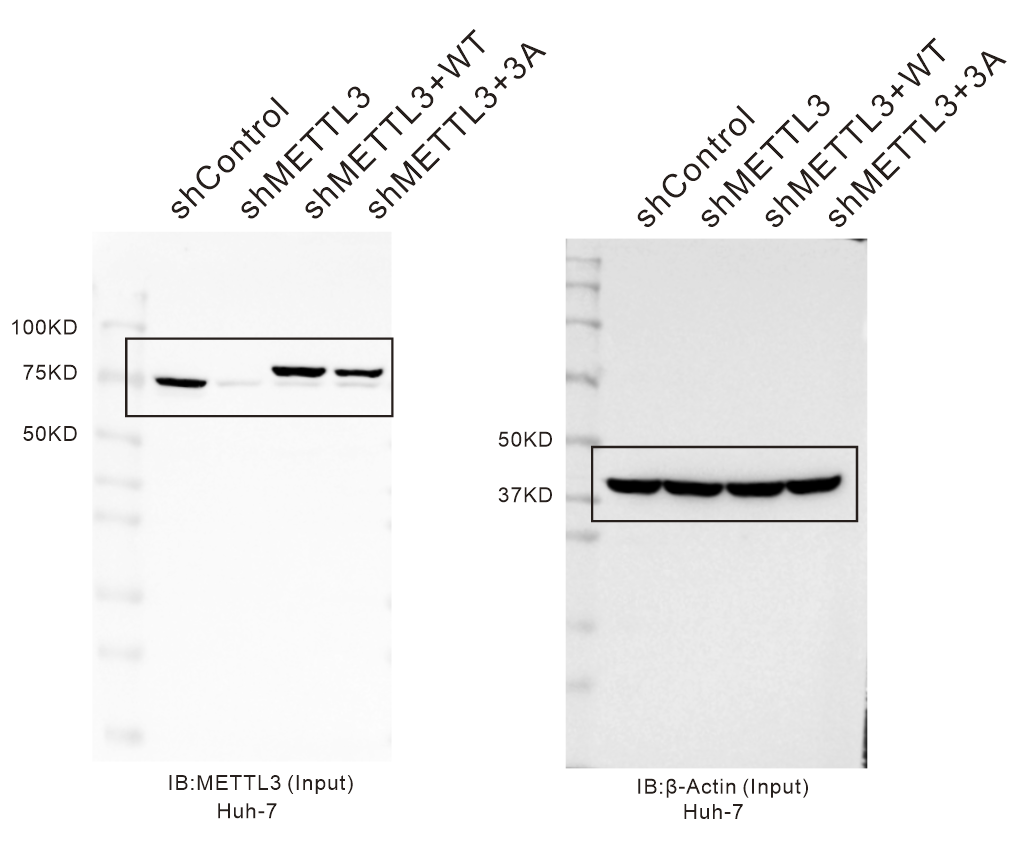


**Figure 5J**


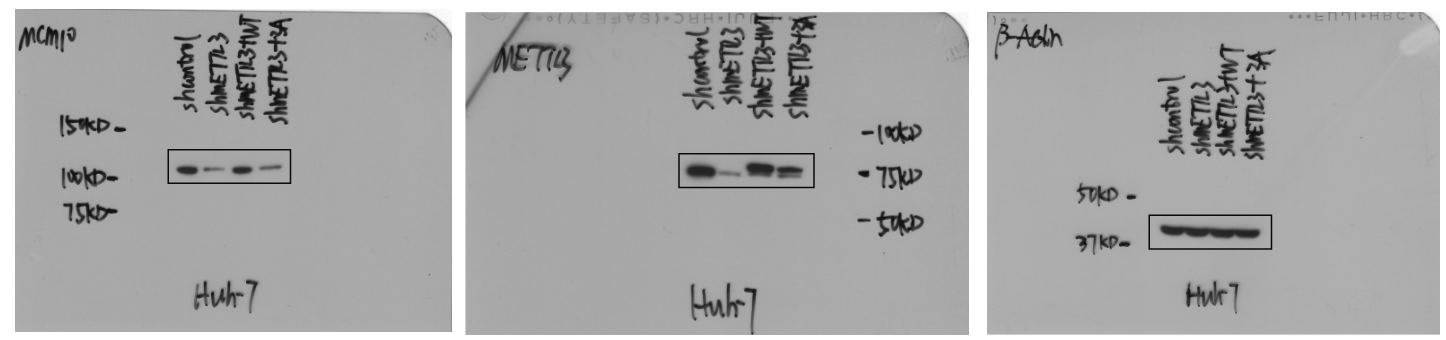


**Figure 6L**


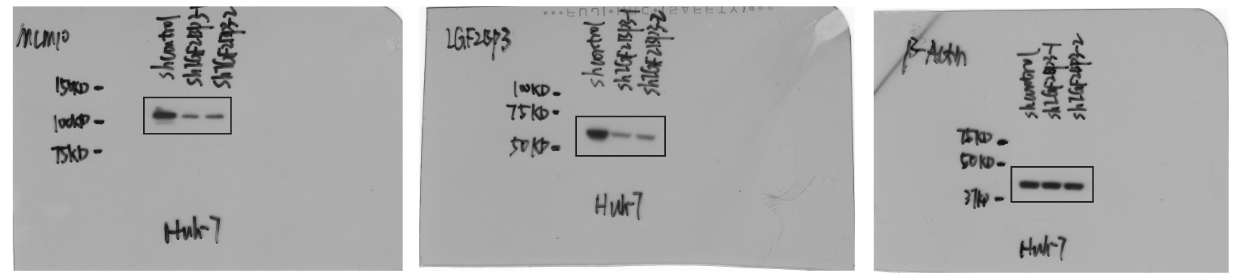


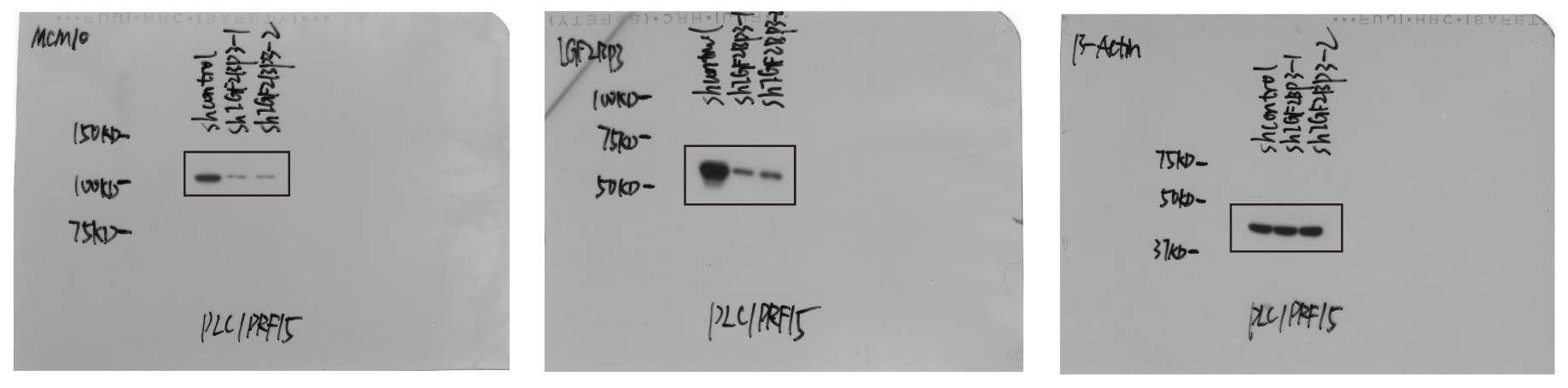


**Figure 7B**


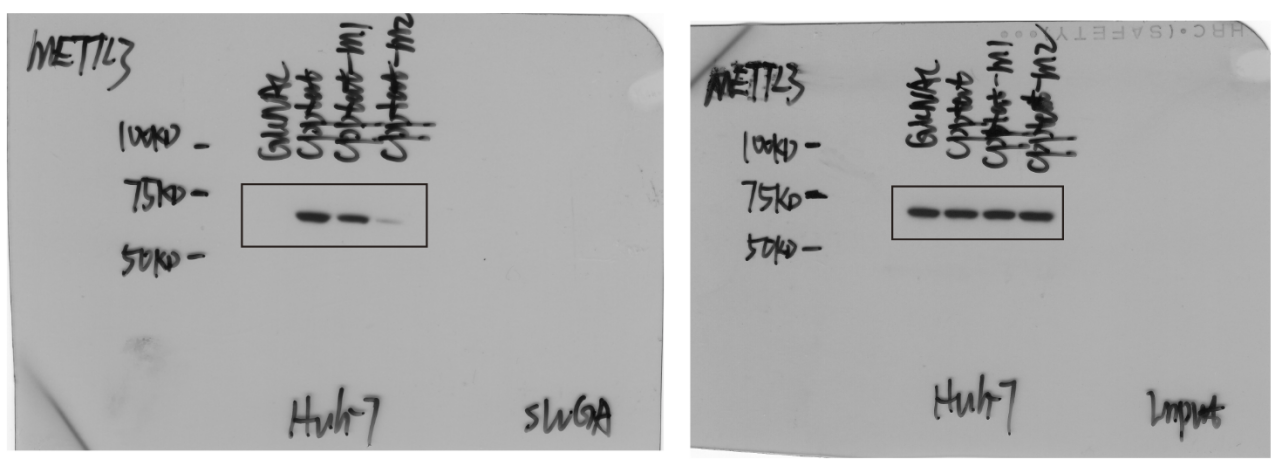


**Figure 7C**


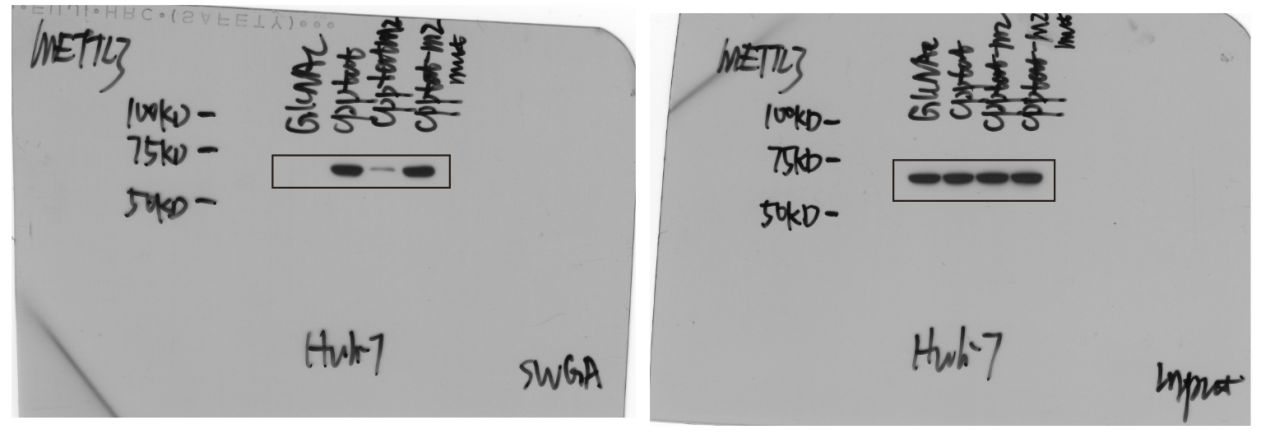


**Figure 7L**


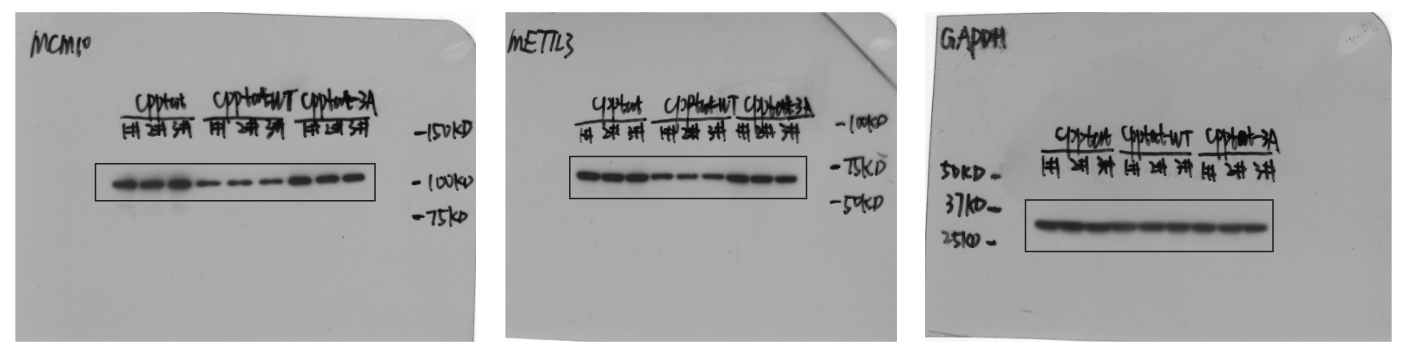


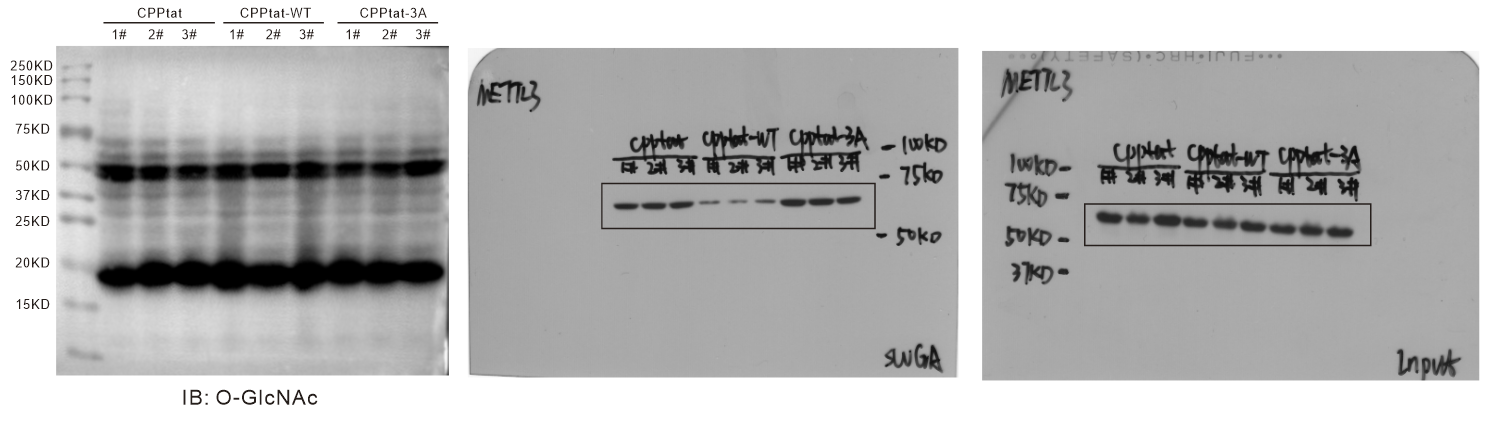


**Figure S1A**


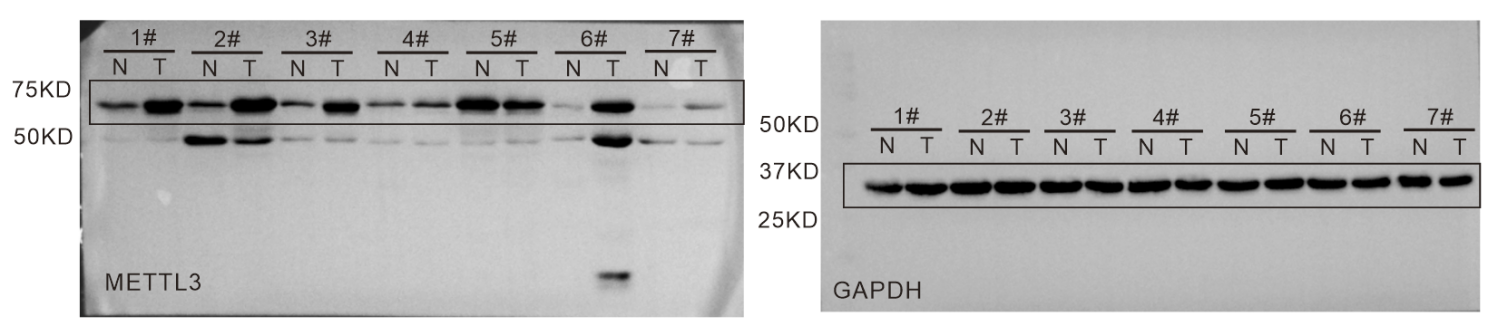


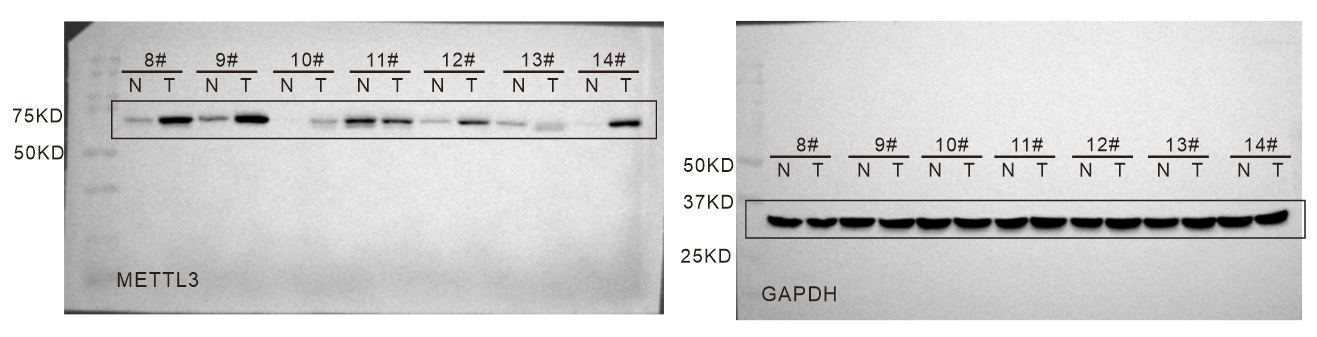


**Figure S1A**


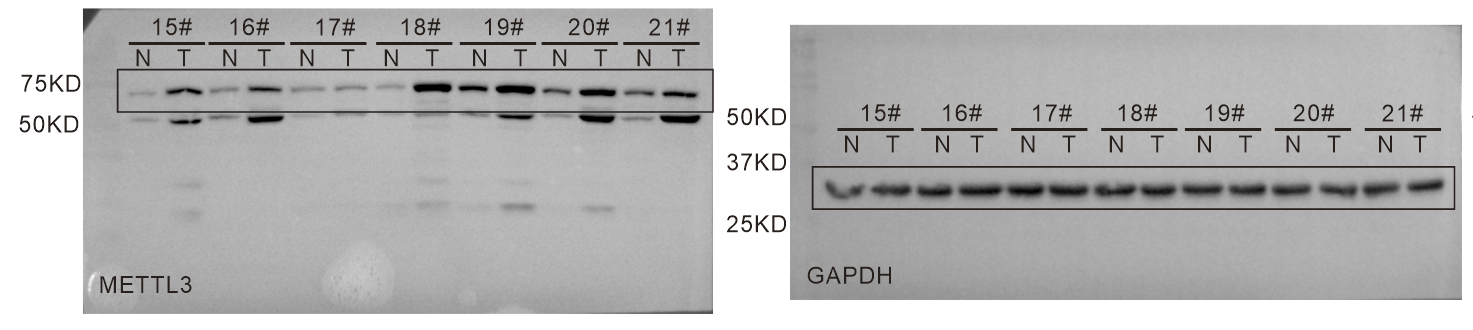


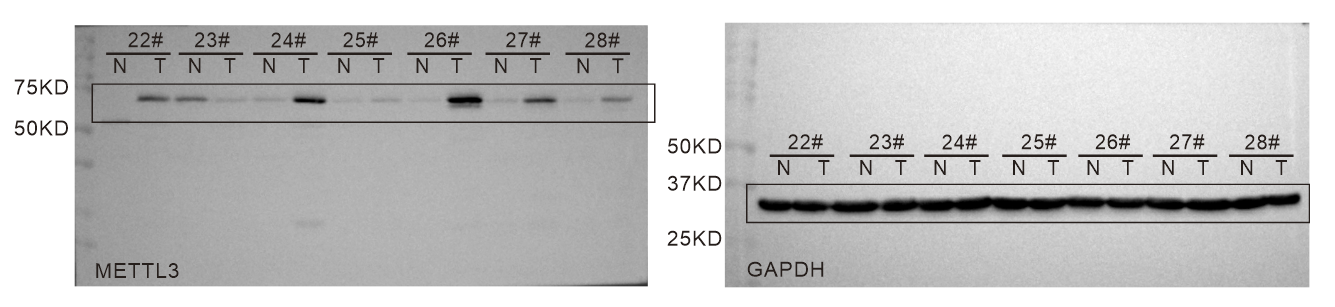


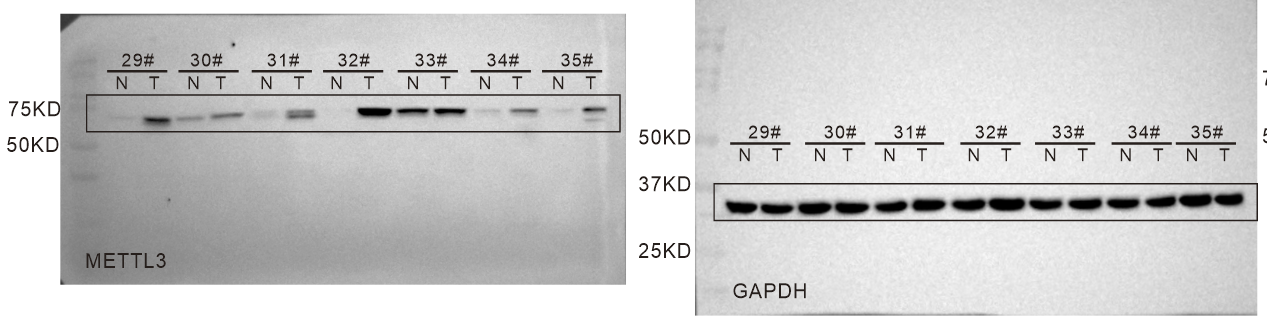


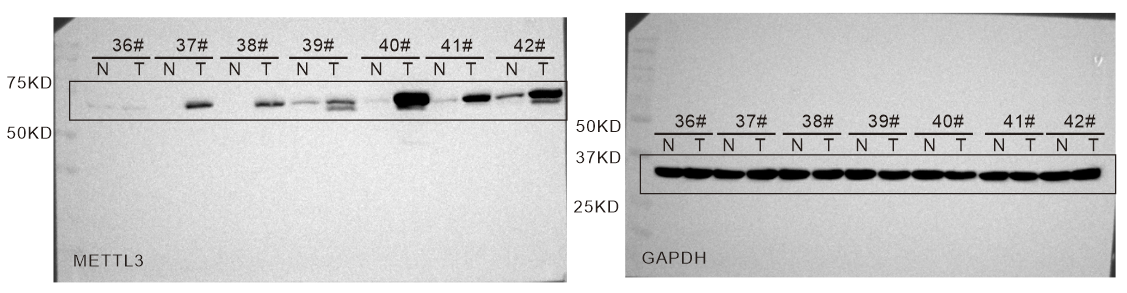


**Figure S1D**


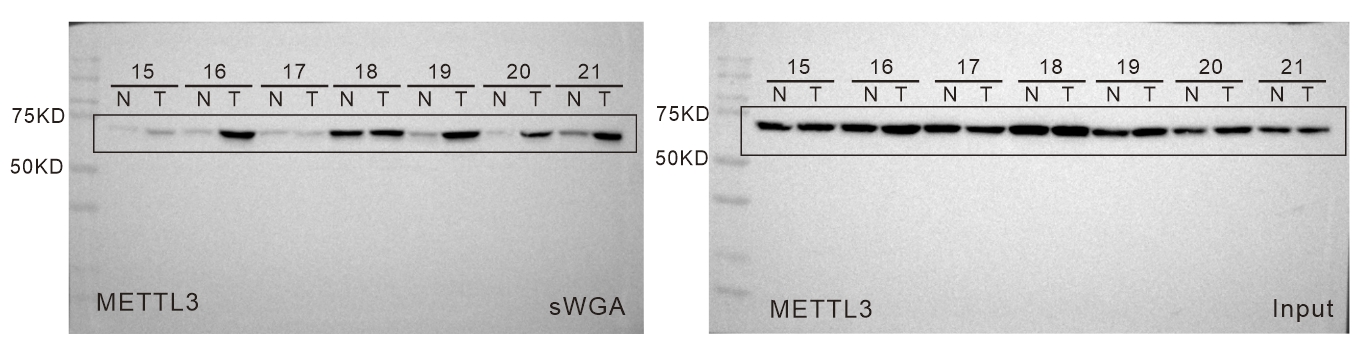


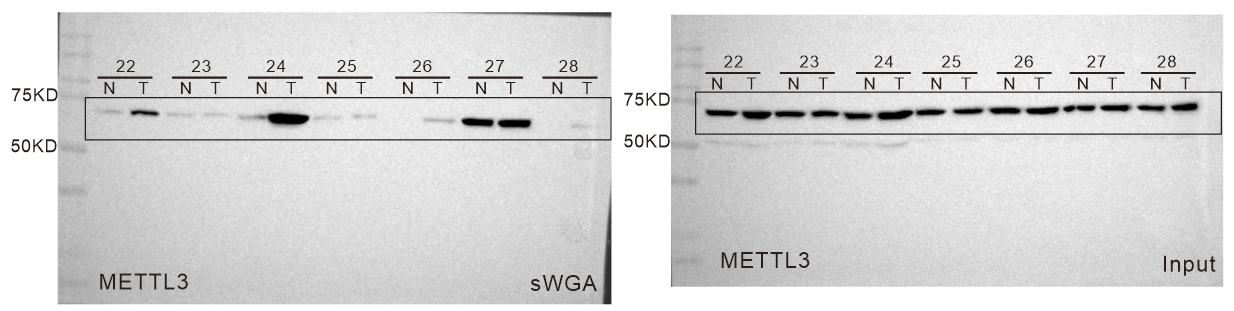


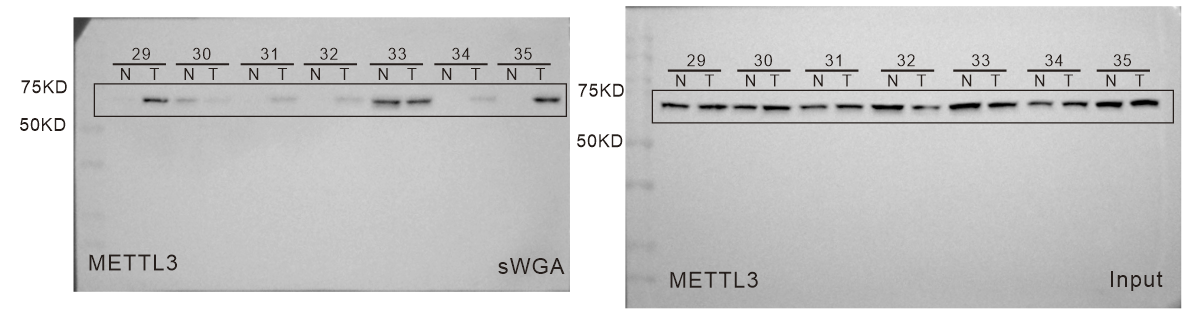


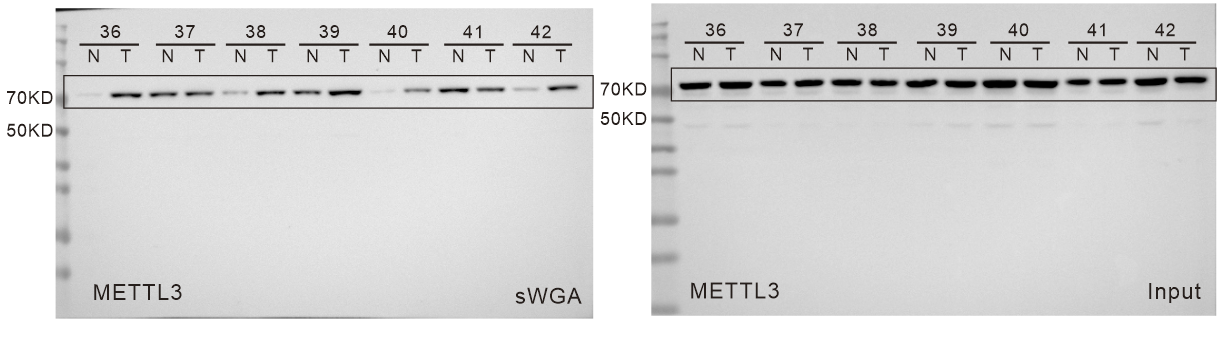


**Figure S2A**


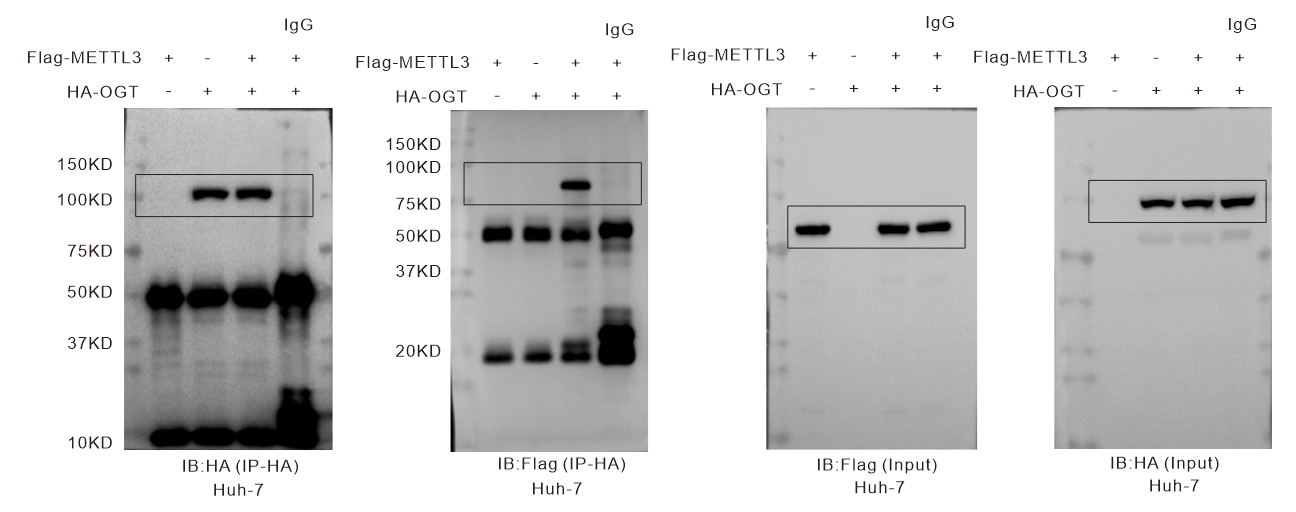


**Figure S2C**


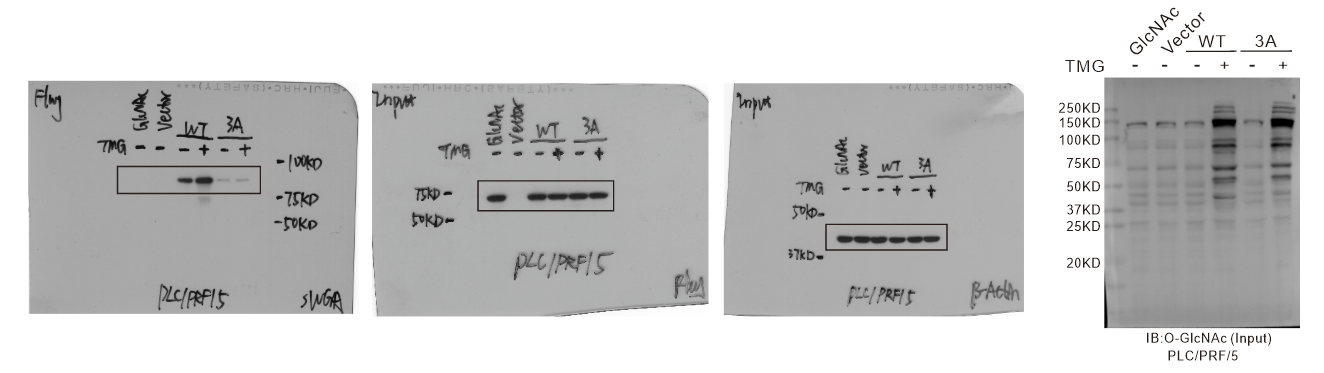


**Figure S3A**


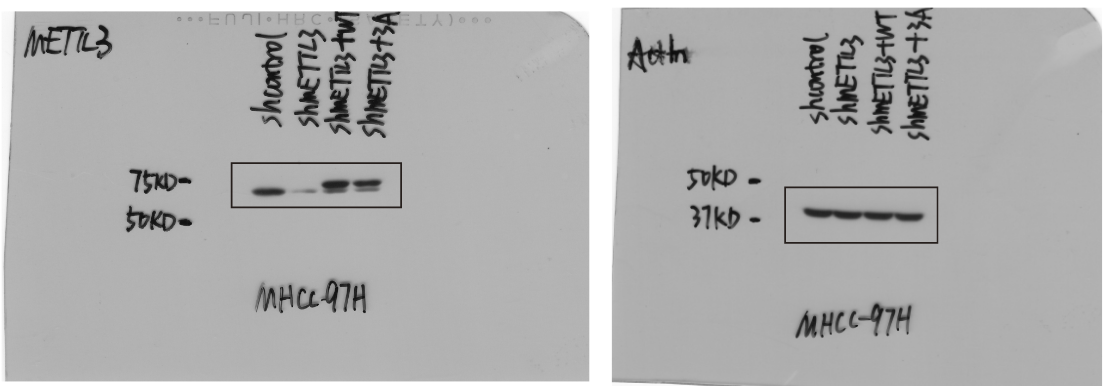


**Figure S4A**


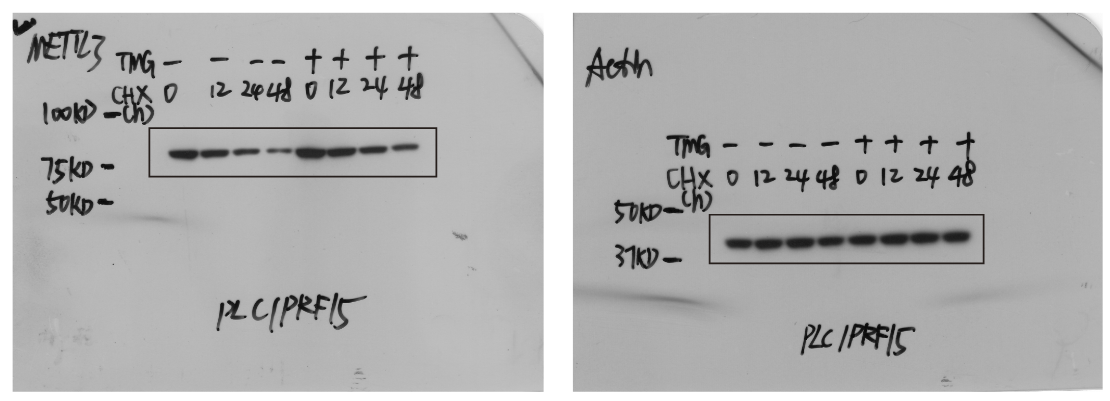


**Figure S4B**


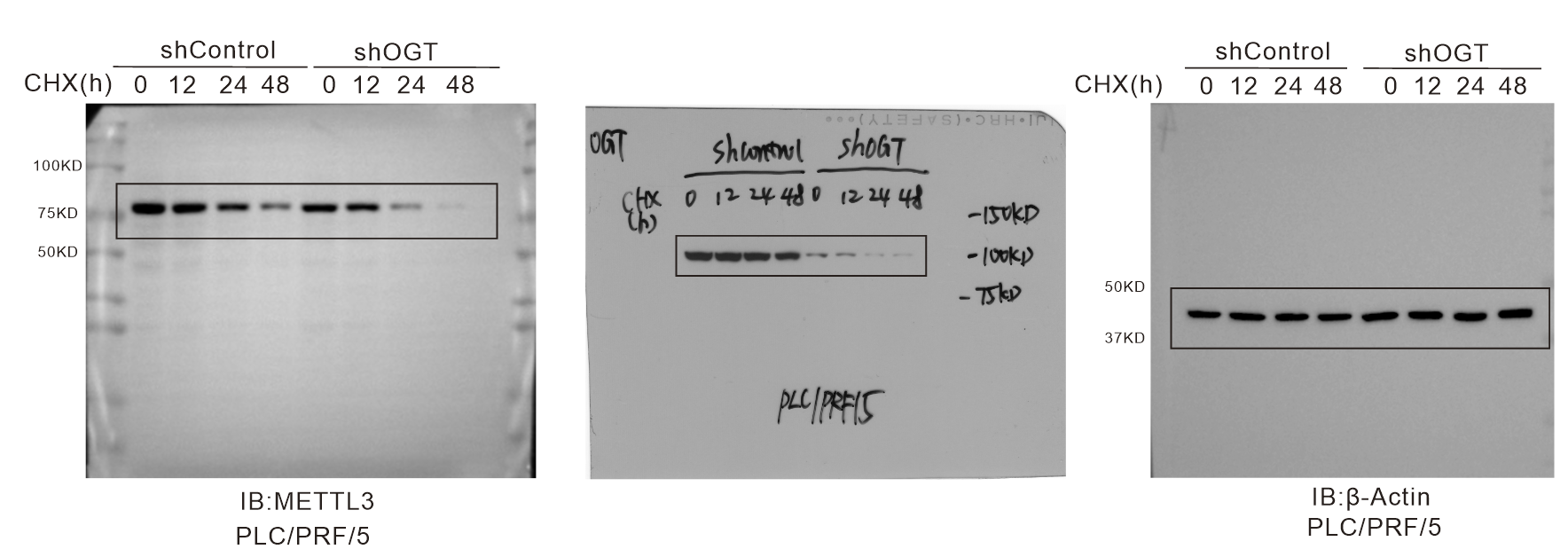


**Figure S4C**


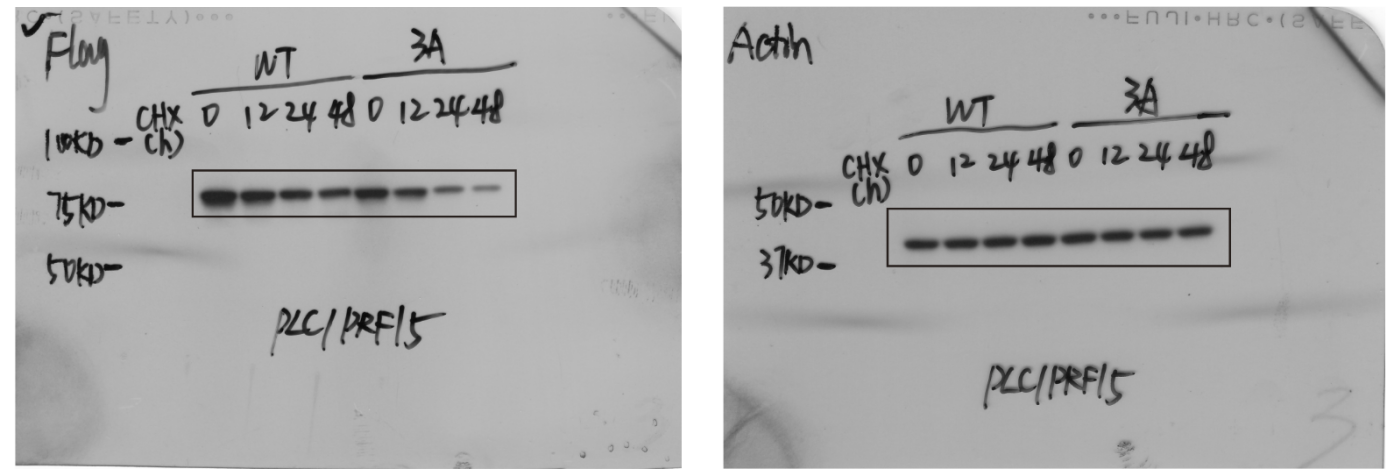


**Figure S4D**


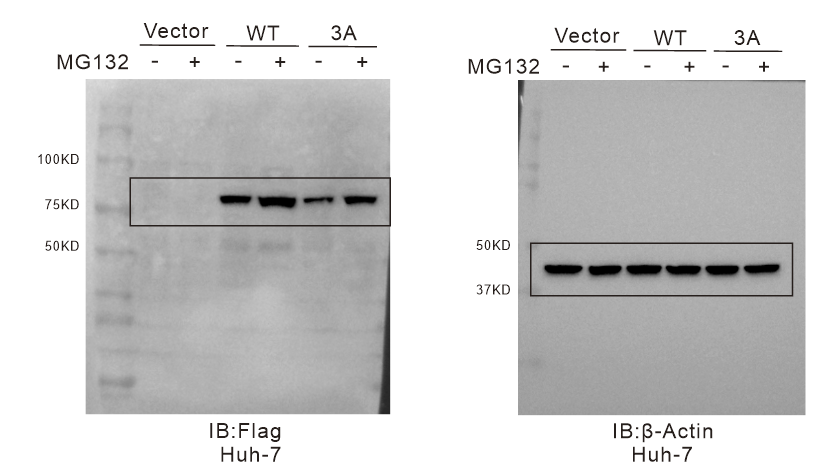


**Figure S4E**


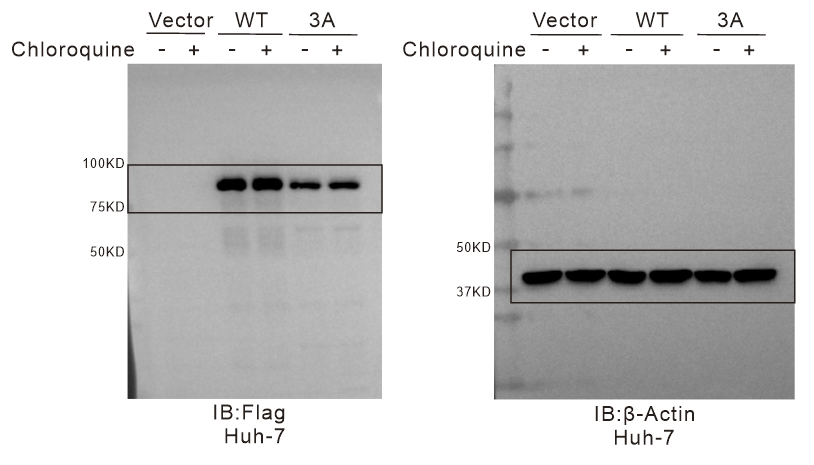


**Figure S4F**


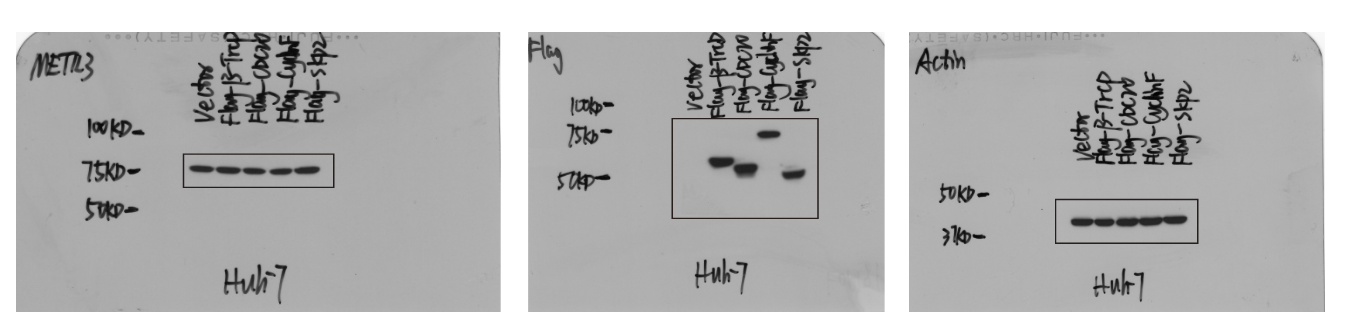


**Figure S4F**


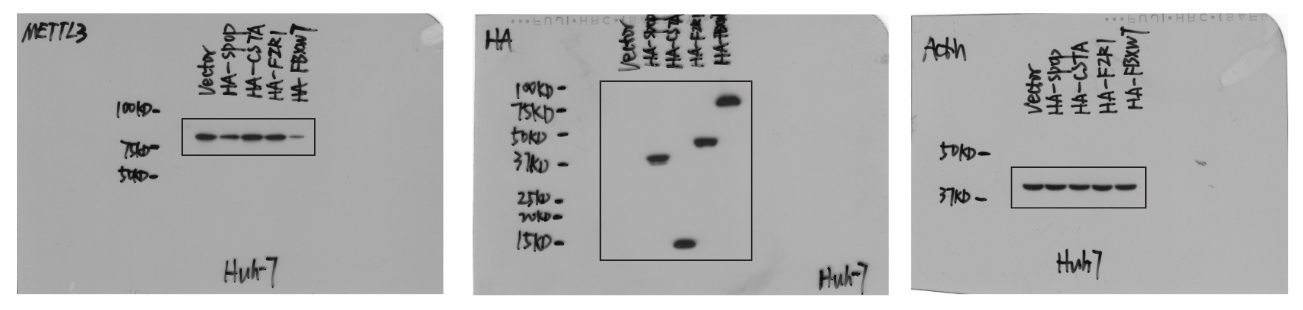


**Figure S4G**


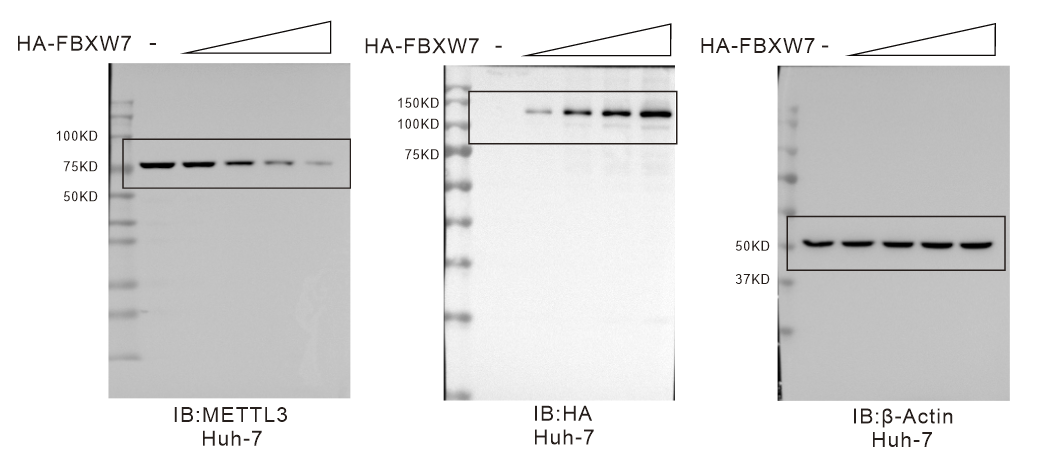


**Figure S4H**


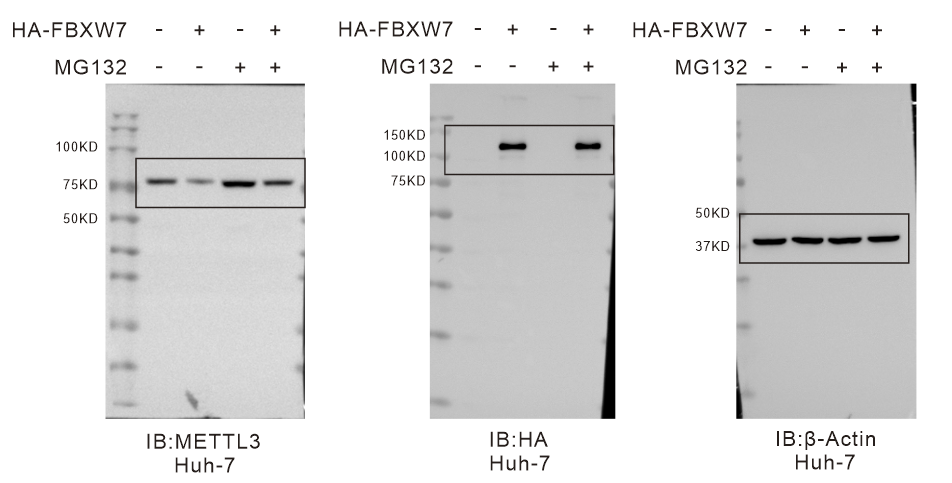


**Figure S4I**


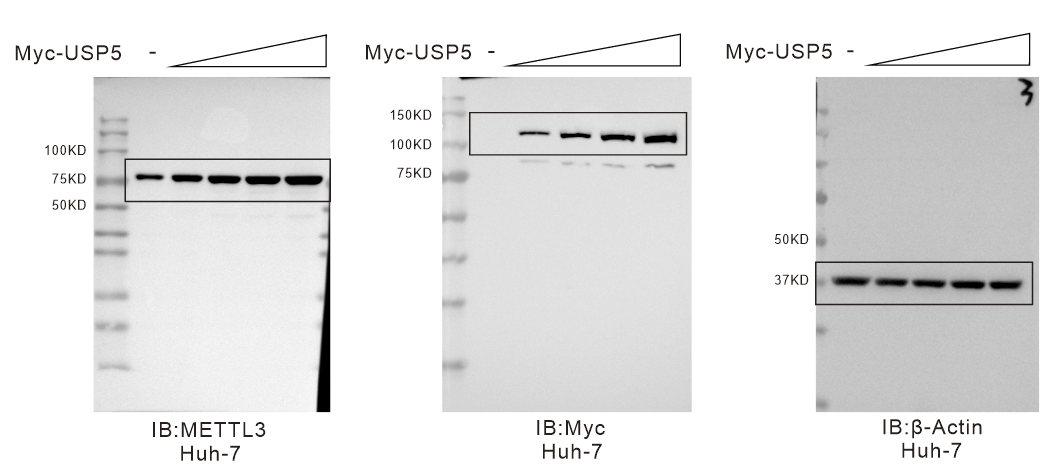


**Figure S4J**


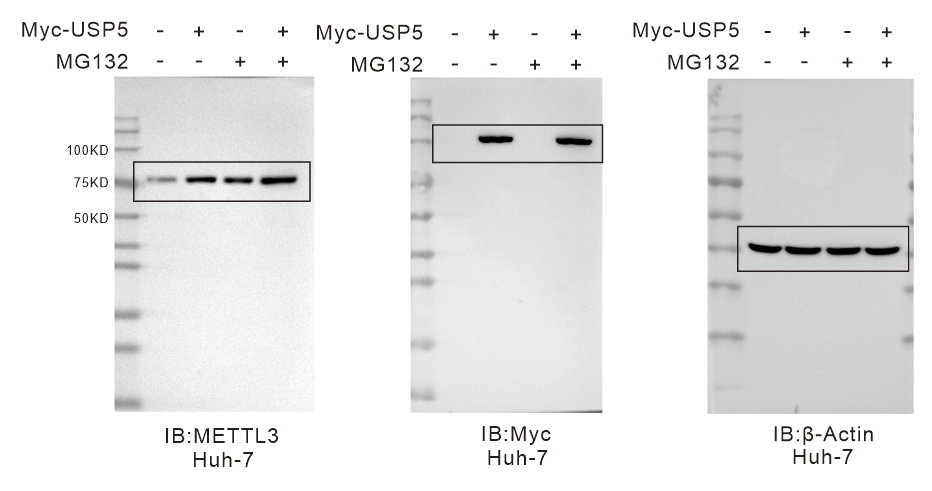


**Figure S4K**


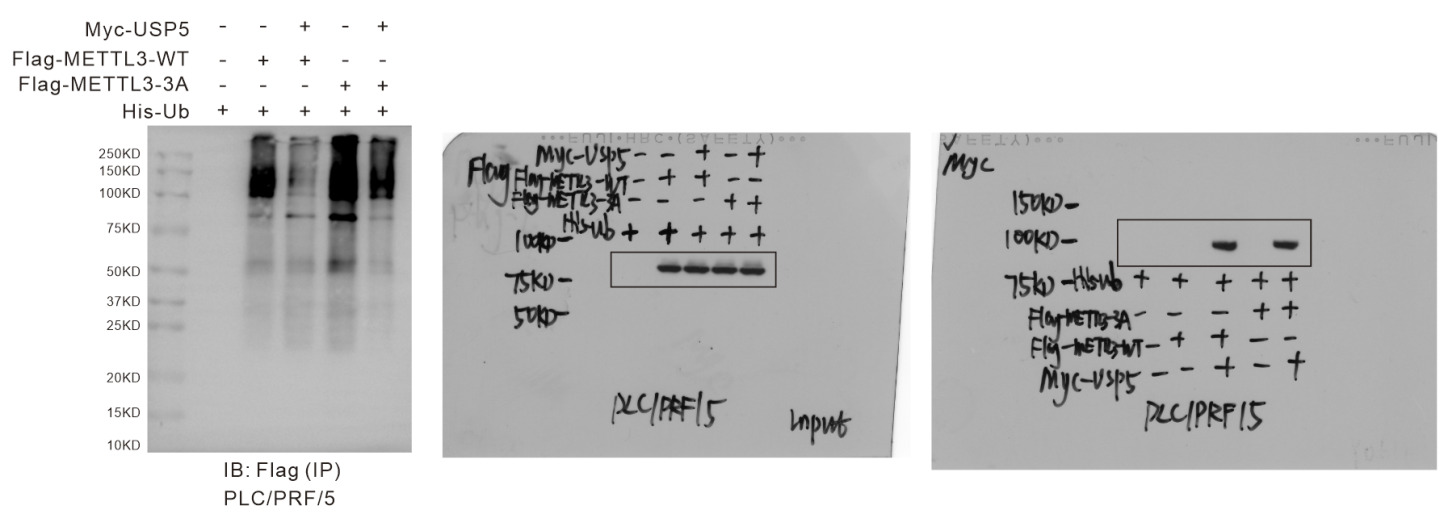


**Figure S5B**


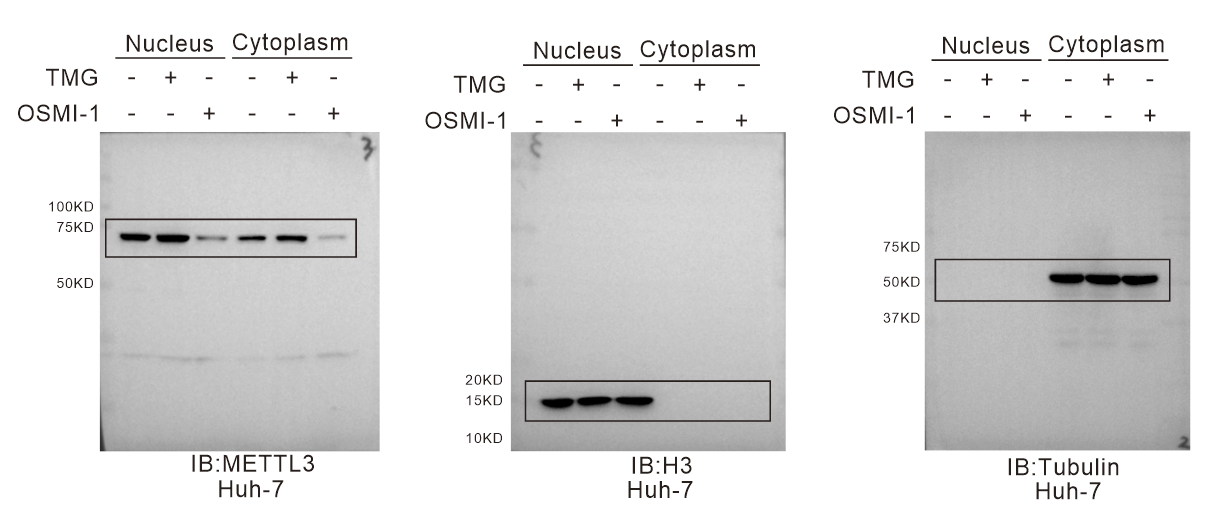


**Figure S5D**


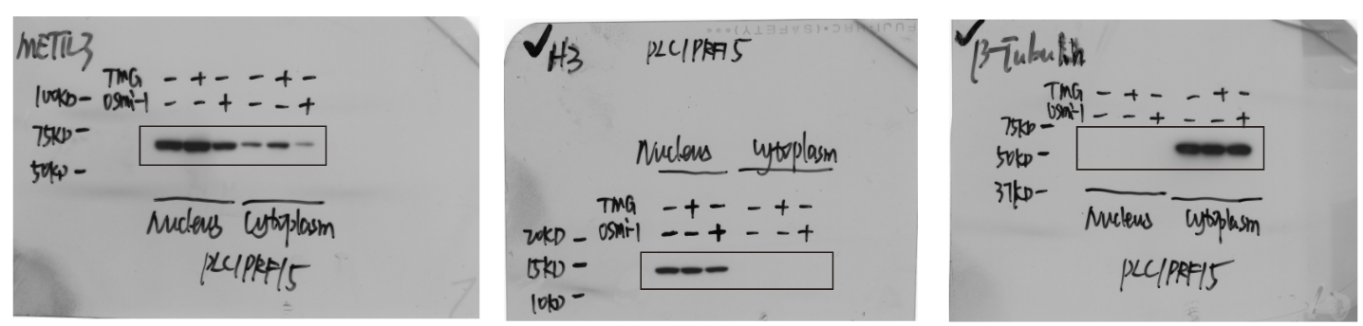


**Figure S5F**


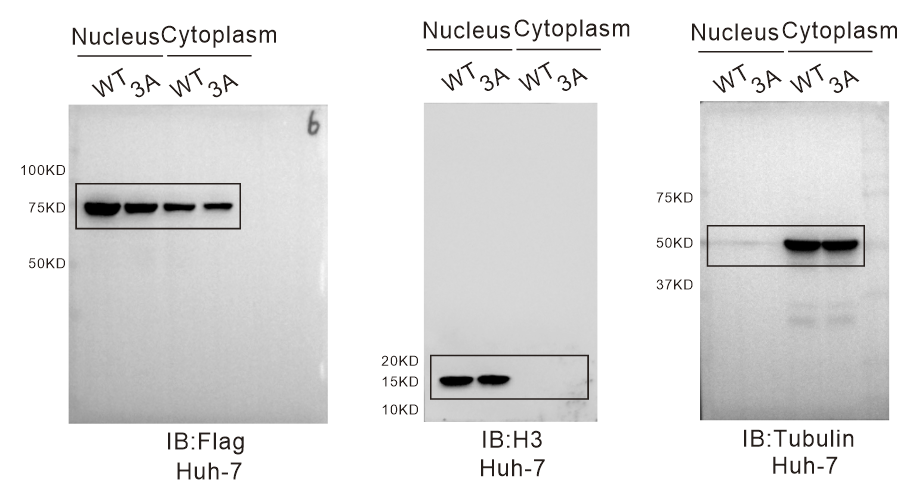


**Figure S5G**


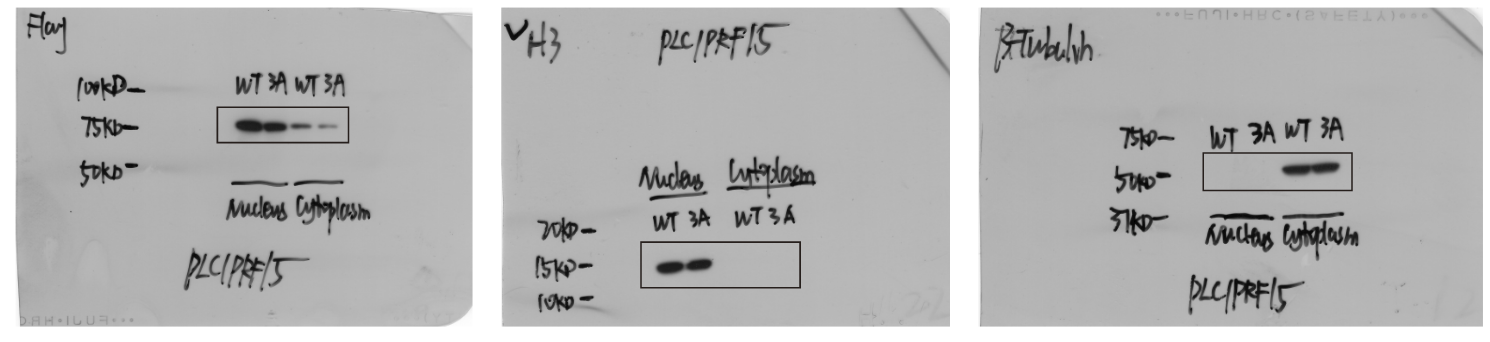


**Figure S6A**


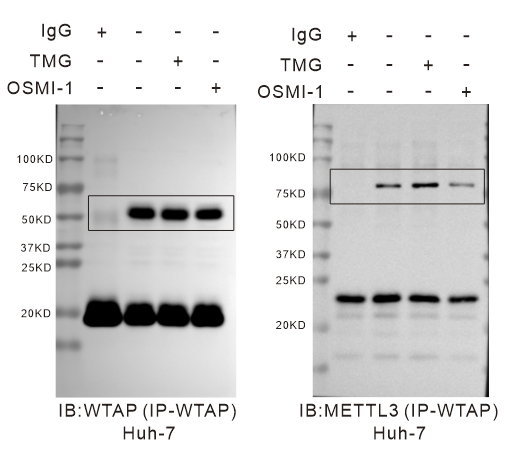


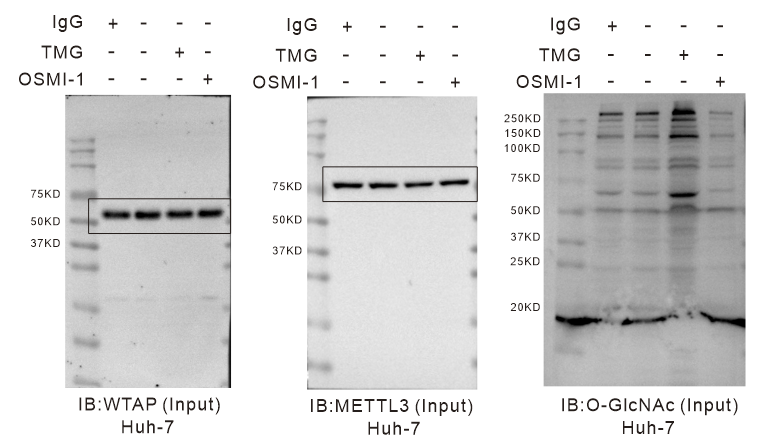


**Figure S6B**


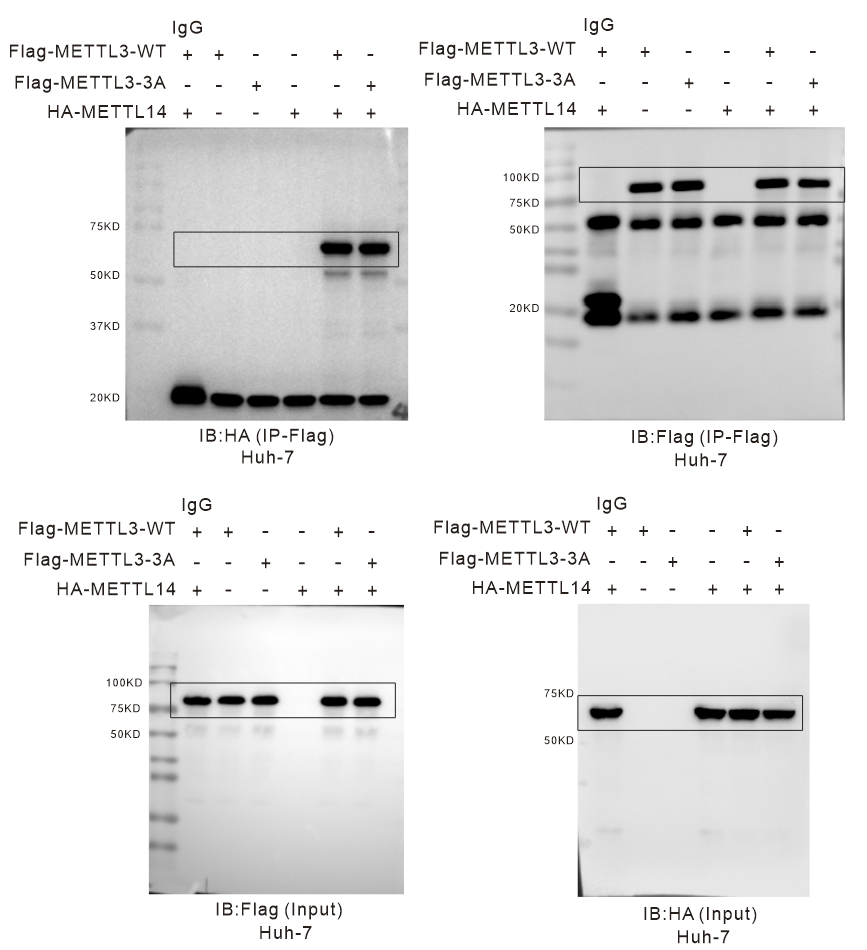


**Figure S6D**


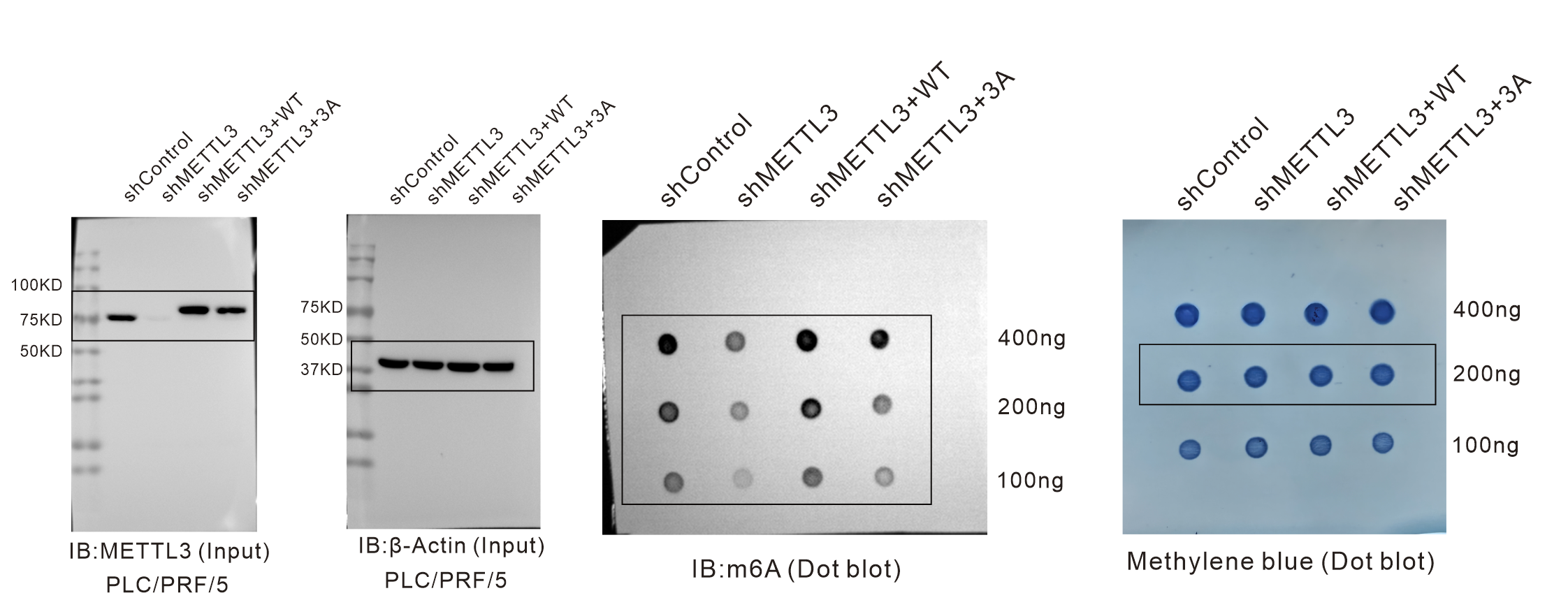


**Figure S6E**


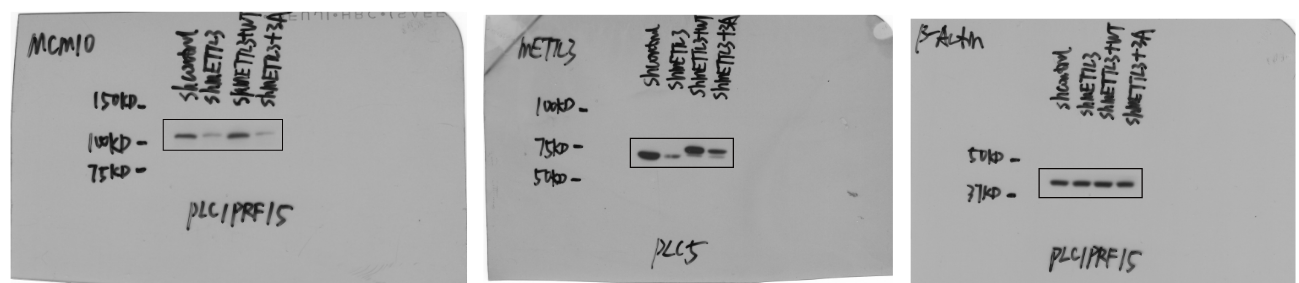


**Figure S7A**


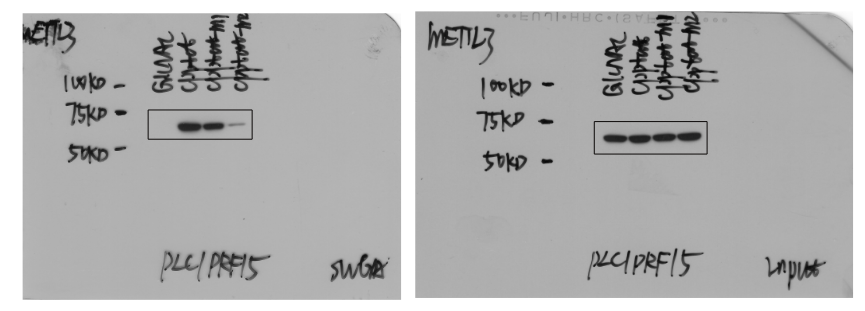


**Figure S7B**


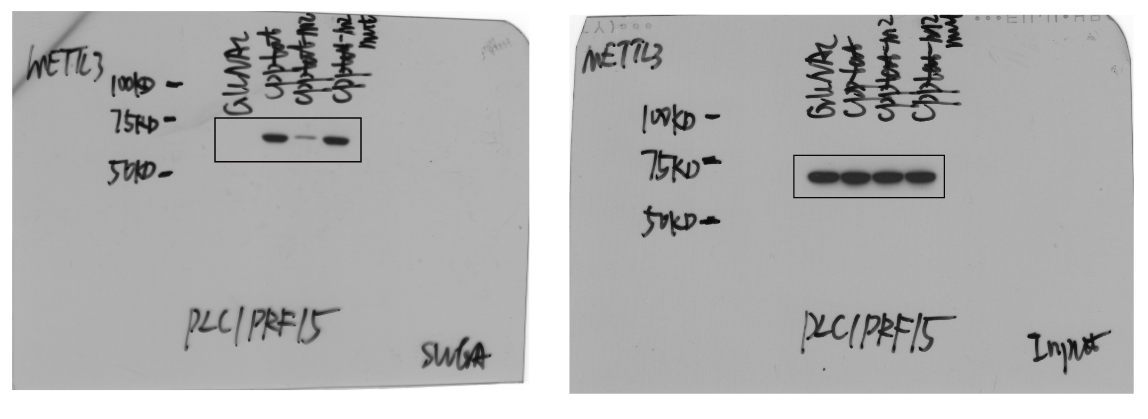

Supplement: Supplementary file 2 — Uncropped Western blots [file 41419_2025_7844_MOESM2_ESM.doc]
